# Virome Analysis of Normal and Growth Retardation Disease-Affected Macrobrachium rosenbergii

**DOI:** 10.1128/spectrum.01462-22

**Published:** 2022-11-29

**Authors:** Dandan Zhou, Shanshan Liu, Guangyu Guo, Xinyi He, Chengguang Xing, Qijin Miao, Gongrui Chen, Xiaolin Chen, Hongyu Yan, Jiamin Zeng, Zhenwen Zheng, Hengwei Deng, Shaoping Weng, Jianguo He

**Affiliations:** a State Key Laboratory of Biocontrol, Southern Marine Sciences and Engineering Guangdong Laboratory (Zhuhai), School of Marine Sciences, Sun Yat-sen University, Guangzhou, China; b School of Life Sciences, Sun Yat-sen University, Guangzhou, China; c School of Ecology, Sun Yat-sen University, Guangzhou, China; d State Key Laboratory of Marine Resource Utilization in South China Sea, Hainan University, Haikou, China; Changchun Veterinary Research Institute

**Keywords:** *Macrobrachium rosenbergii*, growth retardation disease, virome, *Picornavirales*, *Flaviviridae*, cross-species transmission

## Abstract

The giant freshwater prawn, Macrobrachium rosenbergii, is an important aquaculture species in China. Growth retardation disease (GRD) is a common contagious disease in *M. rosenbergii*, resulting in slow growth and precocious puberty in prawns, and has caused growing economic losses in the *M. rosenbergii* industry. To investigate the viral diversity of *M. rosenbergii* and identify potentially high-risk viruses linked to GRD, virome analysis of the GRD-affected and normal *M. rosenbergii* was carried out using next-generation sequencing (NGS). A total of 327 contigs (>500 bp) were related to viral sequences belonging to 23 families/orders and a group of unclassified viruses. The majority of the viral contigs in *M. rosenbergii* belonged to the order *Picornavirales*, with the *Solinviviridae* family being the most abundant in both the diseased and normal groups. Furthermore, 16 RNA viral sequences with nearly complete genomes were characterized and phylogenetically analyzed, belonging to the families *Solinviviridae*, *Flaviviridae*, *Polycipiviridae*, *Marnaviridae*, and *Dicistroviridae* as well as three new clades of the order *Picornavirales*. Notably, the cross-species transmission of a picorna-like virus was observed between *M. rosenbergii* and plants. The “core virome” seemed to be present in the diseased and normal prawns. Still, a clear difference in viral abundance was observed between the two groups. These results showed that the broad diversity of viruses is present in *M. rosenbergii* and that the association between viruses and disease of *M. rosenbergii* needs to be further investigated.

**IMPORTANCE** Growth retardation disease (GRD) has seriously affected the development and economic growth of the *M. rosenbergii* aquaculture industry. Our virome analysis showed that diverse viral sequences were present in *M. rosenbergii*, significantly expanding our knowledge of viral diversity in *M. rosenbergii*. Some differences in viral composition were noted between the diseased and normal prawns, indicating that some viruses become more abundant in occurrences or outbreaks of diseases. In the future, more research will be needed to determine which viruses pose a risk for *M. rosenbergii*. Our study provides important baseline information contributing to disease surveillance and risk assessment in *M. rosenbergii* aquaculture.

## INTRODUCTION

The virome has proven helpful for understanding viral diversity and describing novel viruses in new diseases (1). A viromic method based on metatranscriptomic sequencing not only could detect DNA and RNA viruses with excellent sensitivity but also could show high tolerance to stochastic errors to the relative abundance of viromic taxa (2, 3). This approach has played an important role in discovering novel viruses in various tissue and fecal samples from humans and animals (4–6). Furthermore, more surveys have been conducted to explore the viromes of economically important aquaculture species, including fish (7, 8), crustaceans (9–11), and mollusks (12). Although these studies have greatly expanded the knowledge of viral diversity of the aquatic species, relatively few studies have been conducted on the viral composition of aquatic species in different disease states (13–15). Comprehensive virome analysis of valuable aquaculture species will help to explore potential viral pathogens and further develop diagnostic tools to monitor these viruses, which will be of great help to avoid disease outbreaks resulting in substantial economic losses (9).

The giant freshwater prawn, Macrobrachium rosenbergii, is one of the most economically important aquaculture species and has been widely farmed in China and other Southeast Asian countries (16, 17). China is the largest producer of *M. rosenbergii* aquaculture, reaching 161,888 tonnes in 2020 (18). Despite the exponential growth of *M. rosenbergii* aquaculture in China, the incidence of diseases has increased in parallel (15, 19–22). Since 2010, many cultured giant freshwater prawns have been recorded with growth retardation disease (GRD) in China, characterized by growth retardation and precocious puberty (23, 24). The GRD-affected female prawns began to hold eggs when they were up to only 5 cm in body size, and the male prawns engaged in mating behaviors and grew two large blue claws when the body length was about 6 cm, while normal mature prawns are 8 to 10 cm long (25). More than 2,680 ha of farming ponds in Gaoyou City, Jiangsu Province, were affected to various degrees by GRD in 2014 (26). In recent years, GRD has continuously emerged in the main provinces of *M. rosenbergii* breeding, and the proportion of GRD-affected prawns in farming ponds reached 30 to 40% or even up to 80 to 90% in serious areas (27). Although mass mortalities have not occurred in GRD-affected ponds, GRD in *M. rosenbergii* has resulted in a production decline of more than 50%, causing substantial economic losses for *M. rosenbergii* aquaculture (28). Recently, a few studies have been performed to investigate the cause of GRD. The high prevalence of Enterobacter cloacae in slow-growing giant freshwater prawns observed by Gao et al. (29) suggested that the slow growth of *M. rosenbergii* is probably linked to E. cloacae. Dong et al. (28) detected eight major shrimp pathogens, but none were found in diseased prawns. Furthermore, they have also characterized a novel flavivirus associated with sexual precocity. In addition, some transcriptome analyses have been performed to explore the cause and mechanism of growth retardation of *M. rosenbergii* (25, 30). Other factors were reported to be associated with slow-growth *M. rosenbergii*, including antibiotics (31), long-term inbreeding, and deterioration of germ plasm resources (32). To date, the pathogens causing this disease are unknown. Given the powerful functions of the virome, there is a great necessity to explore the viral diversity of *M. rosenbergii* to screen the high-risk viruses linked to diseases.

In the present study, we investigated the viromes of GRD-affected and normal *M. rosenbergii* based on metatranscriptomic sequencing and revealed the broad diversity of viruses in *M. rosenbergii*. The nearly complete RNA viruses were further characterized and phylogenetically analyzed, expanding the virus diversity of the order *Picornavirales* and the family *Flaviviridae* in *M. rosenbergii*. Comparison of viromes between sick and healthy prawns could help to identify potential “high-risk” viruses. The sequences of these novel viruses could further serve as markers, which would be valuable for developing disease surveillance strategies and investigating their potential risks in disease outbreaks.

## RESULTS

### Overview of virome.

**FIG 1:**
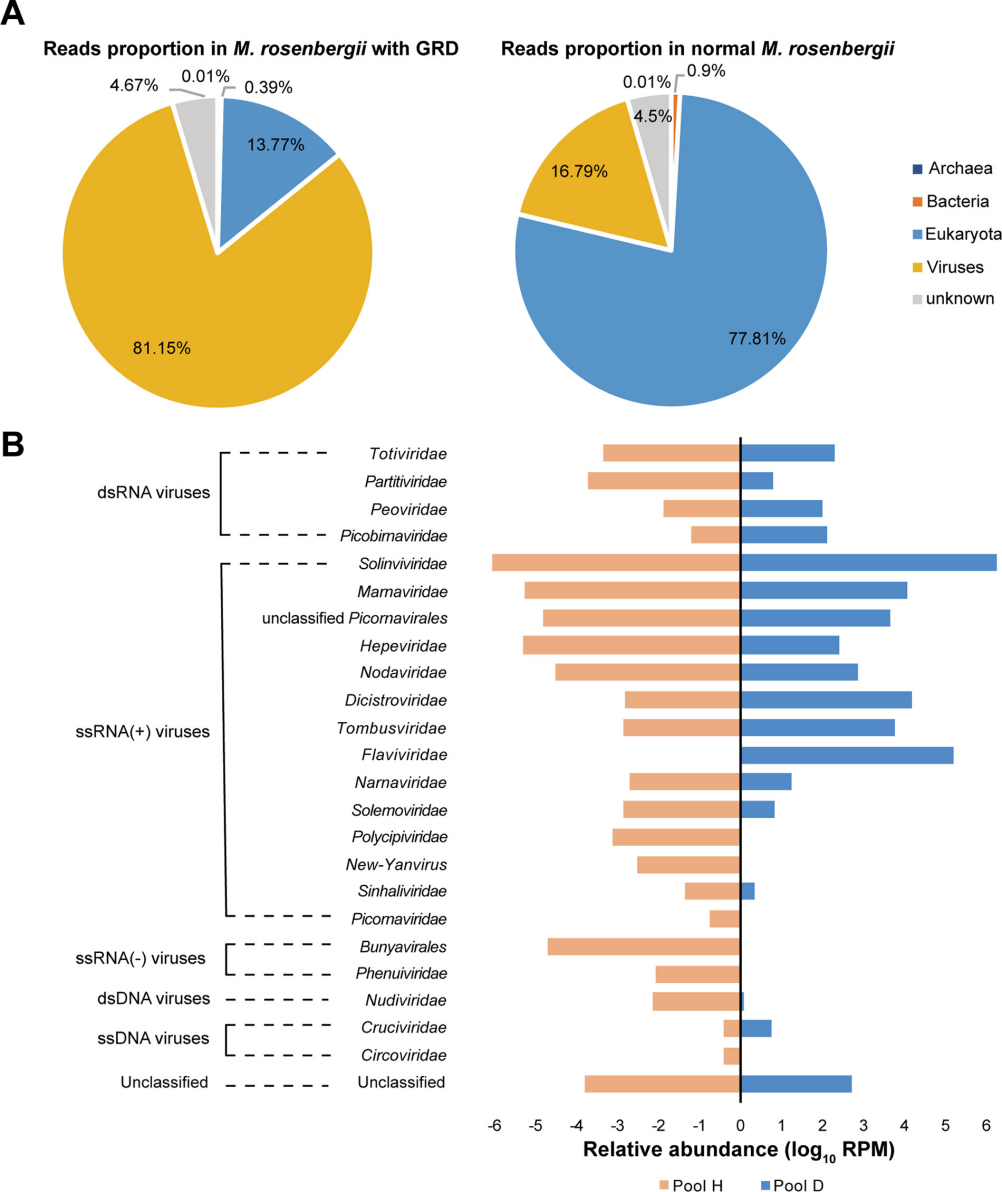
Virome comparison between GRD-affected (pool D) and normal *M. rosenbergii* (pool H). (A) Read proportions of four taxonomic categories (*Eukaryota*, *Bacteria*, *Archaea*, and Viruses) in GRD-affected and normal *M. rosenbergii*. “unknown” represents contigs that got no significant hits in the NCBI nr database. (B) A bar plot displays the relative abundance of viral orders and families in two *M. rosenbergii* groups based on a log_10_ RPM scale.

Fifteen diseased prawns (D) with the gross signs of GRD, including smaller size and precocious puberty, and another pool of 15 healthy prawns (H) without GRD were collected from farms in Jiashan County, Zhejiang Province, China, in 2019. To increase the viral abundance, these prawns were grouped into two pools: the diseased pool (pool D) and the healthy pool (pool H) (see Table S1 in the supplemental material). Overall, 65,370,202 and 33,836,190 trimmed reads were obtained from pools D and H and *de novo* assembled into 79,010 and 53,427 contigs, respectively (see Table S1 in the supplemental material). A total of 12,297 contigs (>500 bp) from two samples were clustered at 95% nucleotide identity over 80% of the length, resulting in 10,798 representative contigs. A total of 5,939 contigs (55%) were assigned to four categories (*Eukaryota*, *Bacteria*, *Archaea*, and Viruses) against the NCBI nonredundant protein (nr) database using DIAMOND. A total of 4,859 contigs got no significant hits, which were called “unknown” (Fig. 1A). The relative abundance of viruses in the diseased group was much higher than that in the normal group, which possessed 81.15% and 16.79% reads in pool D and pool H, respectively (Fig. 1A and Table S1). In contrast, the *Eukaryota* and *Bacteria* proportions (13.77% and 0.39%, respectively) were lower than those in pool H (77.81% and 0.90%, respectively) (Fig. 1A and Table S1).

A total of 327 contigs were invertebrate-associated viruses, 51 (15.6%) viral contigs were shared among D and H, 58 unique contigs were found from pool D, and 218 viral contigs were present only in pool H (Fig. S1). Rarefaction analysis of the viral contigs revealed the identified viral contigs for both GRD-affected and normal prawns were approaching the saturation plateau, suggesting the sampling depths of sequencing data were sufficient, and most viruses were detected in this experiment (Fig. S2). A total of 327 viral contigs were assigned into 23 families/orders under double-stranded RNA (dsRNA) viruses, positive-sense single-stranded RNA (+ssRNA) viruses, negative-sense single-stranded RNA (−ssRNA) viruses, double-stranded DNA (dsDNA) viruses, single-stranded DNA (ssDNA) viruses, and a group of unclassified viruses (Fig. 1B). Sixteen families/orders and some unclassified viruses were shared among pools D and H. A viral sequence of the family *Flaviviridae* was present in high abundance (91,141 mapped reads per million total reads [RPM]) only in pool D. In contrast, viral sequences from the order *Bunuyavirales*, including the families *Circoviridae*, *Phenuiviridae*, *Polycipiviridae*, *New-Yanvirus*, and *Picornaviridae*, were identified only from pool H (Fig. 1B and Table S3). The order *Picornavirales* (46.8% of the viral contigs) was the major viral group in *M. rosenbergii* (Table S2). The family *Solinviviridae* was most abundant in both the diseased and normal groups, and most of the other families were present at very low abundance (<1,000 RPM) (Fig. 1B and Table S3). Some differences in the abundance of viral families were noted between the diseased and normal groups. Specifically, pool D possessed a higher percentage of viral reads from the families *Solinviviridae*, *Dicistroviridae*, and *Tombusviridae* (883,839, 9,581, and 3,883 RPM, respectively), while pool H possessed such reads at lower abundances (643,510, 532, and 565 RPM, respectively) (Fig. 1B and Table S3 in the supplemental material). Reads of the families *Hepeviridae* and *Marnaviridae* and viral sequences belonging to the unclassified *Picornavirales* and *Nodaviridae* were abundant in pool H (127,492, 117,820, 43,694, and 21,156 RPM, respectively) (Fig. 1B and Table S3). In comparison, pool D had very low abundance (189, 7,224, 2,890, and 524 RPM, respectively) (Fig. 1B and Table S3).

Here, 16 RNA viral sequences with nearly complete genomes were identified from the GRD-affected prawns and normal ones (Table 1). These viral sequences belong to the order *Picornavirales* (*n* = 15) and family *Flaviviridae* (*n* = 1). As detailed below, the genome structures of these virus sequences were characterized, and phylogenetic analysis was performed using the amino acid sequences of RNA-dependent RNA polymerase (RdRp) protein.

**TABLE 1:** The nearly complete viral sequences identified in this study

Virus name	Abbreviation	Family or clade	Length (bp)	Closest blastx hit	blastx identity (%)	Viral abundance (RPM) in^*a*^:		Novel viral sequences
						Pool D	Pool H	
Macrobrachium rosenbergii								
Virus 1	MRV1	*Solinviviridae*	14462	Solenopsis invicta virus 7	21.6	883,839	643,510	Yes
Virus 2	MRV2	*Polycipiviridae*	11442	Linepithema humile polycipivirus 1	38.1	0	1,025	Yes
Virus 3	MRV3	*Marnaviridae*	9242	Bivalve RNA virus G3	29.7	285	0	Yes
Virus 4	MRV4	*Marnaviridae*	8887	Beihai picorna-like virus 35	98.3	101	1,434	No
Virus 5	MRV5	*Marnaviridae*	8548	Beihai picorna-like virus 6	64.9	57	946	Yes
Virus 6	MRV6	*Marnaviridae*	9153	Wenzhou picorna-like virus 50	81.4	22	360	Yes
Virus 7	MRV7	*Dicistroviridae*	11342	Beihai picorna-like virus 87	29.8	32	483	Yes
Virus 8	MRV8	*Dicistroviridae*	8587	Dicistroviridae sp. squirrel/UK/2011	28.1	22	6	Yes
Virus 9	MRV9	*Dicistroviridae*	8891	Dicistroviridae sp.	71.5	9,442	17	Yes
Virus 10	MRV10	New clade 1	8867	Changping earthworm virus 1	31.6	137	0	Yes
Virus 11	MRV11	New clade 1	8093	Corey virus	23.1	121	0	Yes
Virus 12	MRV12	New clade 1	9818	Changping earthworm virus 1	28.7	85	6	Yes
Virus 13	MRV13	New clade 1	9883	Changping earthworm virus 1	31.7	33	13	Yes
Virus 14	MRV14	New clade 2	8205	Wenzhou picorna-like virus 39	25.8	77	0	Yes
Virus 15	MRV15	New clade 3	9011	Trichosanthes kirilowii picorna-like virus	98.9	6,722	114,953	No

Infectious precocity virus strain ZJJS2019	IPV strain ZJJS2019	*Flaviviridae*	12594	Infectious precocity virus	99.62	91,142	0	No

a RPM, mapped reads per million total reads.

### Viruses in the order *Picornavirales*.

**FIG 2:**
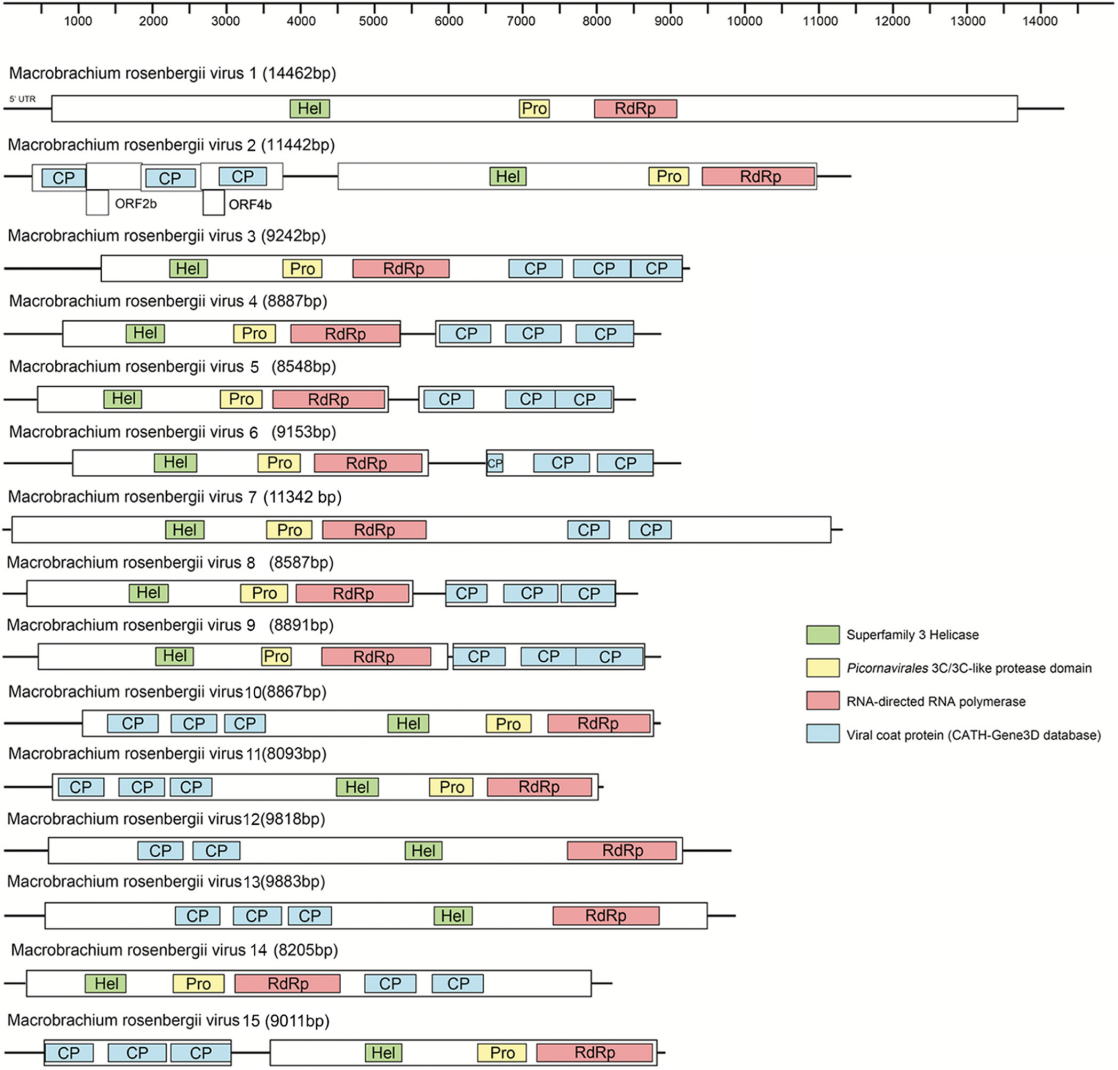
Genome organization of newly identified viral sequences in the order *Picornavirales*.

**FIG 3:**
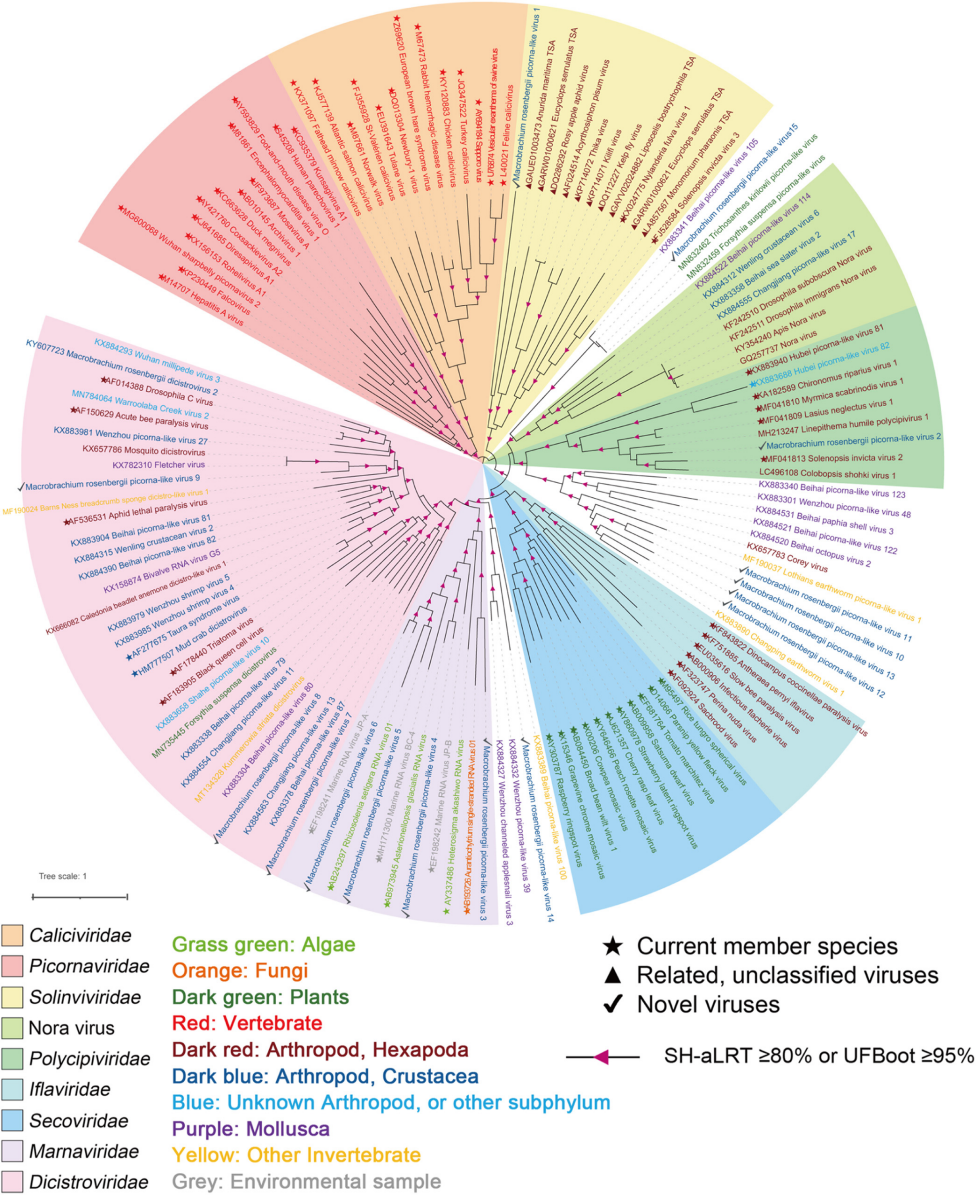
Midpoint-rooted phylogenetic tree of 15 picorna-like viruses, related viral sequences, and representative and unassigned members of the order *Picornavirales*. The tree was based on the amino acid sequence of RNA-directed RNA polymerase (RdRp) and inferred using the maximum likelihood approach implemented in IQ-TREE version 1.6.12. Branch support was evaluated with the Shimodaira-Hasegawa approximate-likelihood ratio test (SH-aLRT) and ultrafast bootstrap (UFBoot). The branches were indicated at the nodes when the SH-aLRT value was ≥80% or the UFBoot value was ≥95%. The background colors indicate different families of viruses. Virus names are marked in different colors based on their host taxonomy. The star indicates the current member species, the triangle indicates related unclassified viruses of families, and the check mark indicates novel picorna-like viruses. The scale bar represents the number of amino acid substitutions per site.

Fifteen picorna-like virus sequences with nearly complete genomes were identified from two pools, and 10 viruses shared a low (21.6% to 38.1%) amino acid identity with the most closely related viral sequences (Table 1). The helicase, protease, and RdRp replication module (Hel-Pro-RdRp) and at least two capsid protein domains were detected in most sequences (Fig. 2). Fifteen viral sequences were assigned to four current families (*Solinviviridae*, *Polycipiviridae*, *Marnaviridae*, and *Dicistroviridae*) and three new clades in the order *Picornavirales* (Fig. 3). Among these, 12 novel RNA viral sequences and three known viral sequences were discovered here.

**FIG 4:**
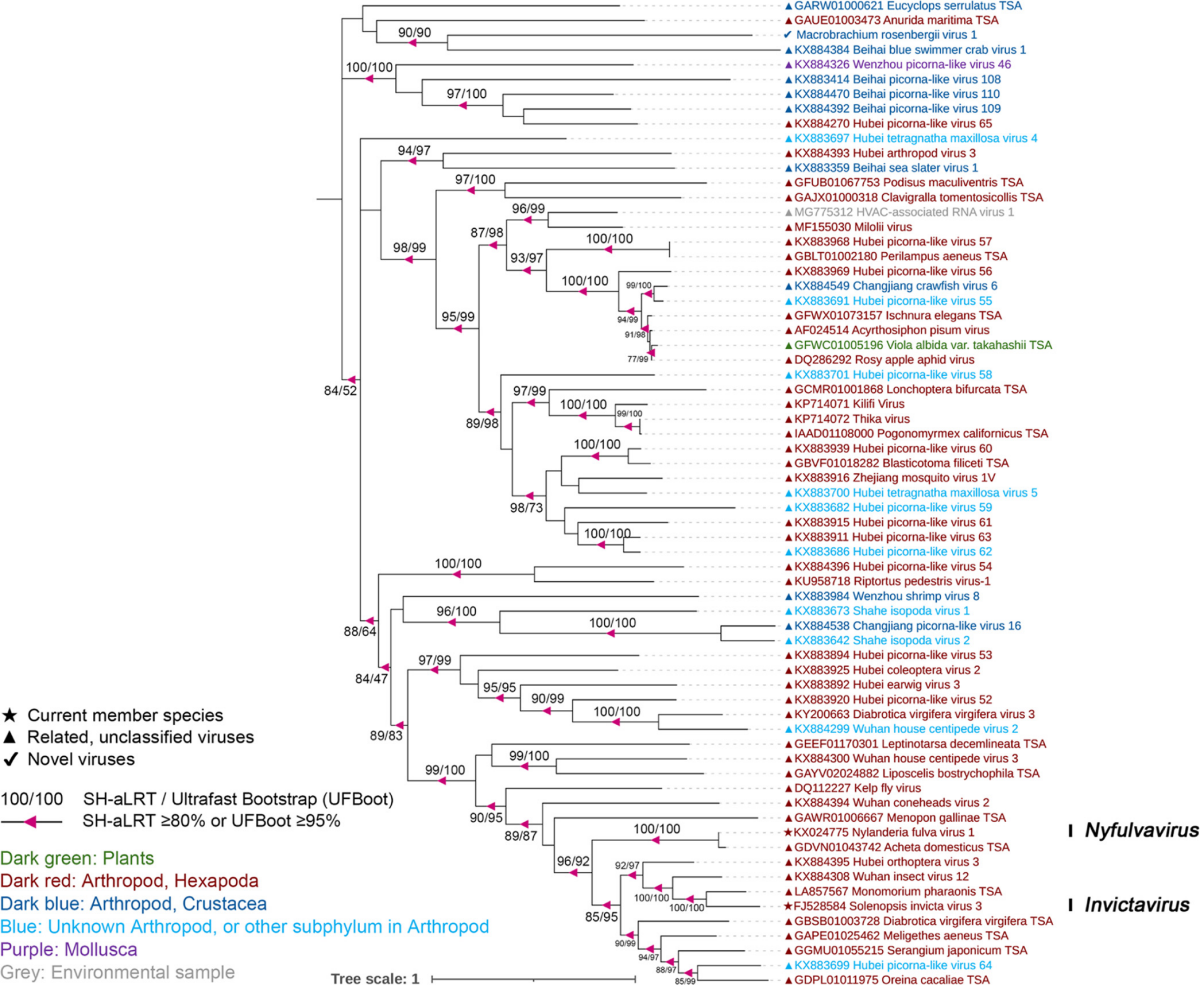
Midpoint-rooted phylogenetic tree of RdRp protein of MRV1, related viral sequences, and representative and unassigned members of *Solinviviridae*. Branch support was evaluated with SH-aLRT and UFBoot. The values are indicated at the nodes when the SH-aLRT value was ≥80% or the UFBoot value was ≥95%. The names of the viruses are marked in different colors based on their host taxonomy. The star indicates the current member species, the triangle indicates related unclassified viruses of families, and the check mark indicates novel picorna-like viruses. The scale bar represents the number of amino acid substitutions per site.

**(i) Viruses in the family *Solinvividae*.** *Solinviviridae*, established in 2017, is a relatively new family of picorna/calici-like viruses, with only two classified solinvivirus species in two genera infecting ants. Still, previous studies have obtained related unclassified virus sequences from various other insects and arthropods (33). One solinvi-like viral sequence, tentatively named Macrobrachium rosenbergii virus 1 (MRV1), was detected in two groups. MRV1 was the most abundant virus in pools D and H, reaching 888,839 and 643,510 RPM, respectively (Table 1). The genome size of MRV1 was 14,462 bp, unlike the typical virus genome length (10 to 11 kb), sharing just 21.6% amino acid identity with Solenopsis invicta virus 7 (SINV-7) across the polyprotein (Fig. 2; Table 1). Phylogenetic analysis using conserved RdRp domains revealed that MRV1 was clustered with other related unclassified viruses as described by Shi et al. (5) (Fig. 4), and a long branch length involved that MRV1 may reveal a novel virus in the family *Solinvivirdae*.

**FIG 5:**
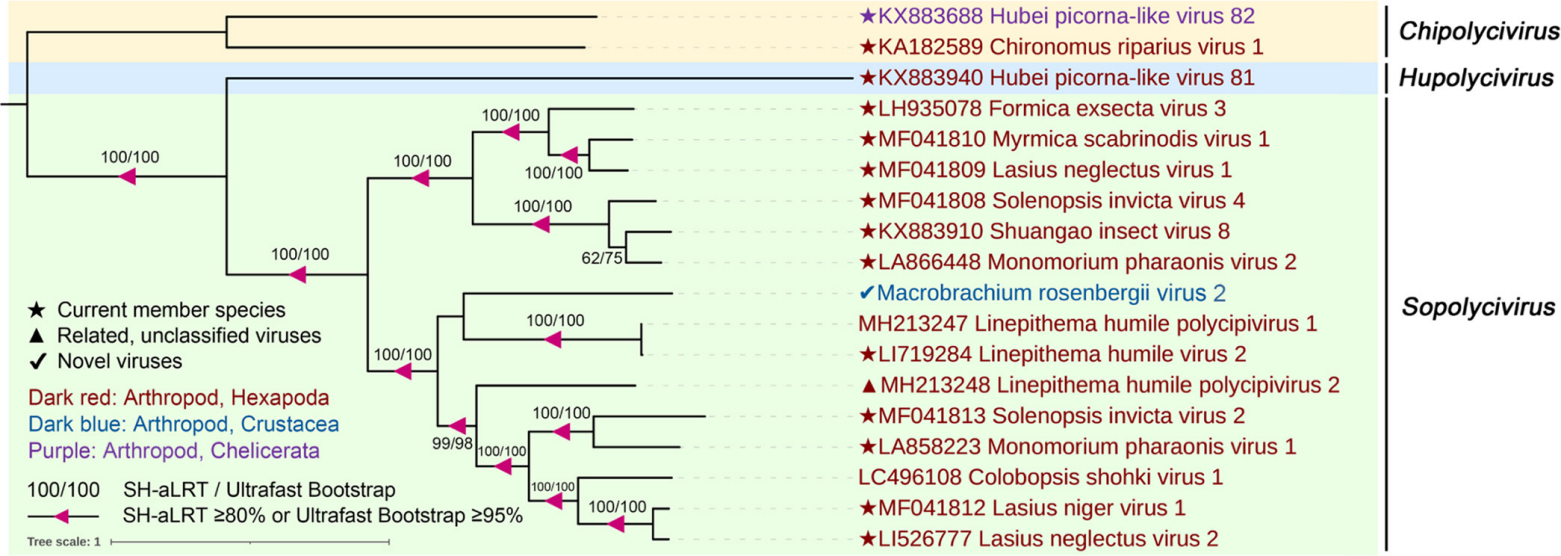
Midpoint-rooted phylogenetic tree of full-length ORF5 amino acid sequences of MRV6, related viral sequences, and representative and unassigned members of *Polycipiviridae*. Branch support was evaluated with SH-aLRT and UFBoot. The values are indicated at the nodes when the SH-aLRT value was ≥80% or the UFBoot value was ≥95%. The background colors show the different genera of *Polycipiviridae*. The names of the viruses are marked in different colors based on their host taxonomy. The star indicates the current member species, the triangle indicates related unclassified viruses of families, and the check mark indicates novel picorna-like viruses. The scale bar represents the number of amino acid substitutions per site.

**(ii) Viruses in the family *Polycipiviridae*.** A novel polycipivirus sequence, namely, Macrobrachium rosenbergii virus 2 (MRV2), was discovered only from pool H. The nearly complete genome of MRV2 was 11,442 bp in length and contained a typical polycipivirus genome organization with four overlapping 5′-proximal ORFs (ORFs 1 to 4) and one long 3′ ORF (ORF5) in the sense direction (Fig. 2). In addition, it had an additional ORF (ORF2b) overlapping ORF2, which is also found in all members of the genus *Sopolycivirus*, encoding a small protein containing a predicted transmembrane domain (34). Moreover, MRV2 also had a new small ORF (ORF4b) without any significant homology (Fig. 2). This was consistent with a previous report for Linepithema humile polycipivirus 1 (LhuPcV1) found in Linepithema humile (35). MRV2 was closely related to LhuPcV1, sharing 38.1% amino acid identity across ORF5 (RdRp) (Table 1). Phylogenetic analysis of the ORF5 nonstructural polyprotein placed MRV2 within the *Sopolycivirus* genus in a well-supported clade (100% bootstrap support) (Fig. 5). According to the International Committee on Taxonomy of Viruses (ICTV) guidelines, species demarcation within the *Sopolycivirus* genus requires >10% amino acid sequence divergence from other species across the ORF5 protein (34). These results support that MRV2 should be considered a novel virus of the genus *Sopolycivirus* in the family *Polycipiviridae*. It is interesting to note that nearly all members of the genus *Sopolycivirus* appear to be detected in ant species (family Formicidae), except for Shuangao insect virus 8 (ShiV8), which is an insect-associated virus from an insect mixed sample in the previous study (5). MRV2 is the first to identify a polycipivirus in the genus *Sopolycivirus* from the crustacean species *M. rosenbergii*, expanding the diversity and host range in the family *Polycipiviriade*.

**FIG 6:**
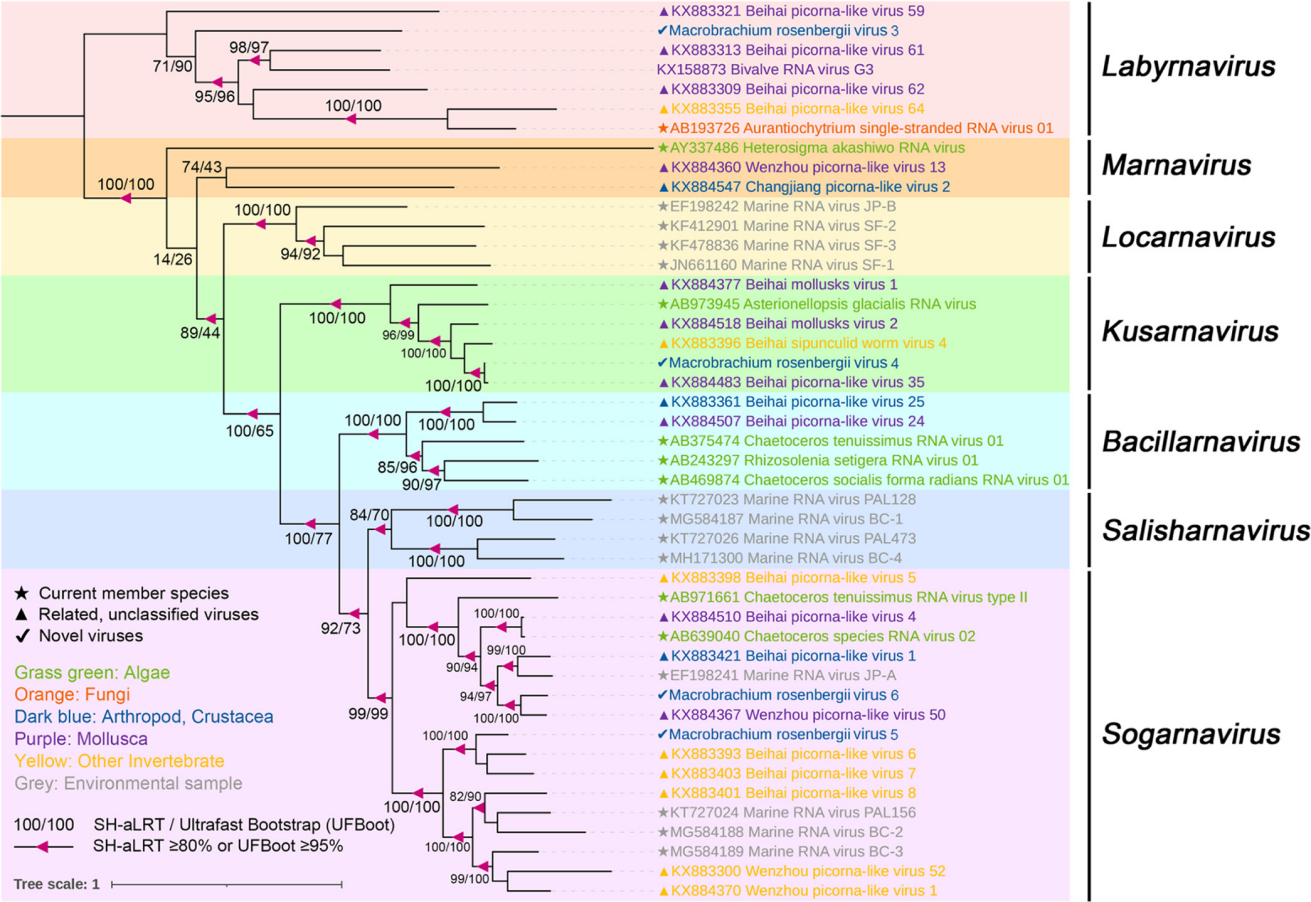
Midpoint-rooted phylogenetic tree of RdRp protein of the family *Marnaviridae*, including identified viruses in this study and related representative viruses. Branch support was evaluated with SH-aLRT and UFBoot. The values are indicated at the nodes when the SH-aLRT value was ≥80% or the UFBoot value was ≥95%. The background colors show the different genera of *Marnaviridae*. The names of the viruses are marked in different colors based on their host taxonomy. The star indicates the current member species, the triangle indicates related unclassified viruses of families, and the check mark indicates novel picorna-like viruses. The scale bar represents the number of amino acid substitutions per site.

**(iii) Viruses in the family *Marnaviridae*.** Four sequences of marna-like viruses were identified from two pools that fell within the recently established family *Marnaviridae*, which we named Macrobrachium rosenbergii virus 3 (MRV3), Macrobrachium rosenbergii virus 4 (MRV4), Macrobrachium rosenbergii virus 5 (MRV5), and Macrobrachium rosenbergii virus 6 (MRV6). The lengths of the four viral sequences were 9,242, 8,887, 8,548, and 9,153 bp, respectively. MRV4, MRV5, and MRV6 contained two nonoverlapping ORFs, while MRV3 contained a single ORF encoding the polyprotein (Fig. 2). Regardless of the fact that the genome structures of these viral sequences were different, the nonstructural proteins and structural proteins were encoded in the 5′ region and 3′ region, respectively (Fig. 2). MRV3 was highly divergent, sharing only 29.7% amino acid identity with bivalve RNA virus G3 found in bivalves (12). MRV4 shared 98.3% amino acid identity with Beihai picorna-like virus 35 strain BHBJDX17224 identified from penaeid shrimp (5) (Table 1). MRV5 and MRV6 shared 64.9% and 81.4% amino acid identities across the polyprotein to Beihai picorna-like virus 6 and Wenzhou picorna-like virus 50, respectively (5) (Table 1). MRV5 shared 33.27% and 31% amino acid similarities to MRV6 across the nonstructural and structural proteins, respectively. According to a recent proposal to the ICTV, species demarcation with the family *Marnaviridae* requires >10% and >25% amino acid divergence from other species across the RdRp and capsid protein, respectively (36). Phylogenetic analysis placed four viruses into three genera (*Labyrnavirus*, *Kusarnavirus*, and *Sogarnaviru*) of the family *Marnaviridae* (Fig. 6). MRV3 was a novel virus belonging to the genus *Labyrnavirus* with 70.3% divergence, whereas MRV4 and Beihai picorna-like virus 35 were the same virus within the genus *Kusarnavirus* (Fig. 6). MRV5 and MRV6 fell within the genus *Sogarnavirus* (100% bootstrap support), which suggested that MRV5 and MRV6 were two novel viruses of the genus *Sogarnavirus* in the family *Marnaviridae* (Fig. 6).

**FIG 7:**
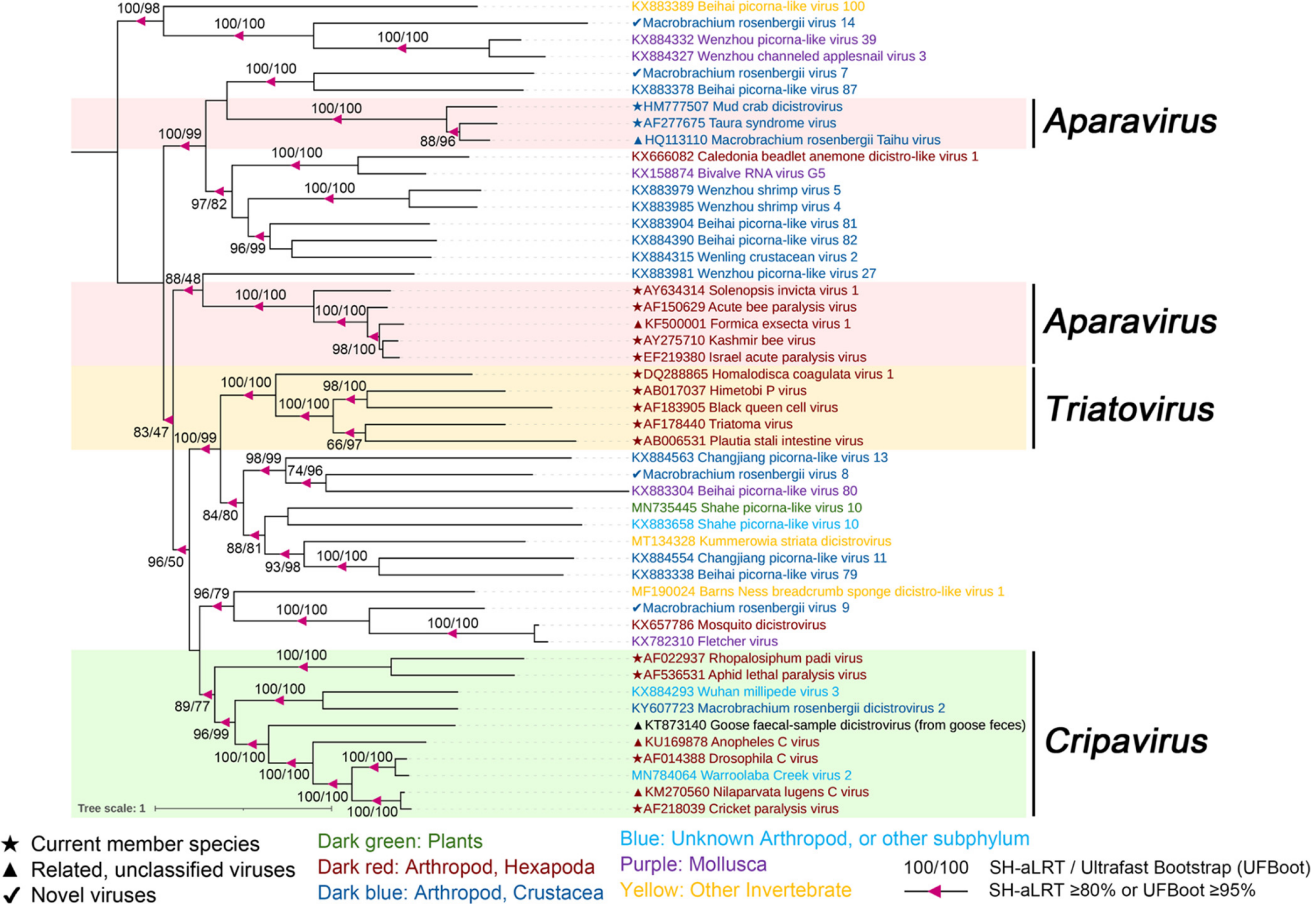
Midpoint-rooted phylogenetic tree of the nonstructural polyprotein, containing the RNA helicase, cysteine protease, and RdRp, including the viruses identified in this study and related representative viruses. Branch support was evaluated with SH-aLRT and UFBoot. The values are indicated at the nodes when the SH-aLRT value was ≥80% or the UFBoot value was ≥95%. The background colors show the different genera of *Dicistroviridae*. The names of the viruses are marked in different colors based on their host taxonomy. The star indicates the current member species, the triangle indicates related unclassified viruses of families, and the check mark indicates novel picorna-like viruses. The scale bar represents the number of amino acid substitutions per site.

**(iv) Viruses in the family *Dicistroviridae*.** Here, we obtained three dicistrovirus sequences from pool D and pool H. Macrobrachium rosenbergii virus 7 (MRV7) was 11,342 bp long, encoding only one large polyprotein, unlike the typical dicistroviruses genome organization containing two nonoverlapping ORFs (Fig. 2). MRV7 shared an amino acid identity of only 29.8% with Beihai picorna-like virus 87 (BPV87) (Table 1), which in turn was predicted to have only one ORF and was assigned to the “*Dicistroviridae* cluster” within the “Picorna-Calici” clade (5). The genome sizes of Macrobrachium rosenbergii virus 8 (MRV8) and Macrobrachium rosenbergii virus 9 (MRV9) were 8,587 and 8,891 bp, respectively, and the viruses displayed typical dicistrovirus genome structures, with two nonoverlapping ORFs encoding the replication enzyme polyprotein and the capsid protein (Fig. 2). They all showed limited sequence identity with unclassified dicistroviruses: 28.1% and 71.5%, respectively (Table 1). Current ICTV-recognized species in the family *Dicistroviridae* were separated by greater than 10% amino acid distance in the capsid protein, suggesting that these three viruses found in this study were all novel viruses (37). Phylogenetic analysis showed that these viruses were clustered into three separate clades: MRV7 was clustered with the crustacean-infecting members of genus *Aparavirus*, whereas MRV8 and MRV9 formed two new sister clades to the genera *Triatovirus* and *Cripavirus* within other unclassified dicistro-like viruses, respectively (Fig. 7), and might represent two new genera in the family *Dicistroviridae*.

**(v) Viruses in the unclassified *Picornavirales*.** Six highly divergent viral sequences were found in two groups, namely, Macrobrachium rosenbergii virus 10 (MRV10), Macrobrachium rosenbergii virus 11 (MRV11), Macrobrachium rosenbergii virus 12 (MRV12), Macrobrachium rosenbergii virus 13 (MRV13), Macrobrachium rosenbergii virus 14 (MRV14), and Macrobrachium rosenbergii virus 15 (MRV15), which fell outside any well-defined family in the order *Picornavirales* (Fig. 3). MRV10, MRV11, MRV12, and MRV13 had a higher abundance in pool D than H (Table 1). They had similar genome organizations with a single ORF containing the structural proteins at the 5′ end and the nonstructural proteins at the 3′ end (Fig. 2). For MRV10 and MRV11, the polyprotein contained six conversed domains, including RNA helicase (Hel), cysteine protease (Pro), RdRP, and three capsid proteins (Fig. 2). In the case of MRV12 and MRV13, the cysteine protease domain was not identified, and two capsid proteins were predicted in MRV12 (Fig. 2). MRV11 shared 23.1% amino acid identity with Corey virus, whereas MRV10, MRV12, and MRV13 were all closely related to Changping earthworm virus 1 (5), with 31.6%, 28.7%, and 31.7% amino acid identity, respectively (Table 1). These four viruses exhibited slow polyprotein amino acid identity (27.49% to 38.8%), suggesting that they were four different viruses. Phylogenetic analysis showed that these four viruses clustered together and formed a high divergent clade, sister to the family *Polycipiviridae* (Fig. 3 and Fig. S5), indicating that the four novel viral sequences might potentially represent a novel viral family in the order *Picornavirales*. MRV14 was present only in pool D and shared an amino acid identity of 25.8% with the Wenzhou picorna-like virus 39 (5) (Table 1). Phylogenetic analysis showed that MRV14 was located on a sister branch of the *Dicistroviridae* and *Marnaviridae* families (Fig. 3) and formed an outgroup with three relatives inside the order *Picornavirales*, likely to represent a novel family.

Instead, the abundance of MRV15 was higher in pool H than in D (Table 1). MRV15 exhibited 99% amino acid identity over 100% coverage to Trichosanthes kirilowii picorna-like virus (TKPV) and Forsythia suspensa picorna-like virus (FSPV) identified from the plants Trichosanthes kirilowii and Forsythia suspensa, respectively (38) (Table 1). MRV15, TKPV, and FSPV formed a monophyletic cluster, sister to the Nora viruses in the phylogeny of the order *Picornavales* (Fig. 3). This indicated that they were the same virus at the level of species. Notably, we confirmed the presence of MRV15 in our samples using reverse transcription-PCR (RT-PCR) followed by Sanger sequencing, and 73.9% (17/23) of GRD-affected prawns collected from Huzhou Province in 2020 were positive in the prevalence investigation of MRV15 (Fig. S3), likely indicating cross-species transmission among *M. rosenbergii* and plants.

### A virus in the family *Flaviviridae*.

**FIG 8:**
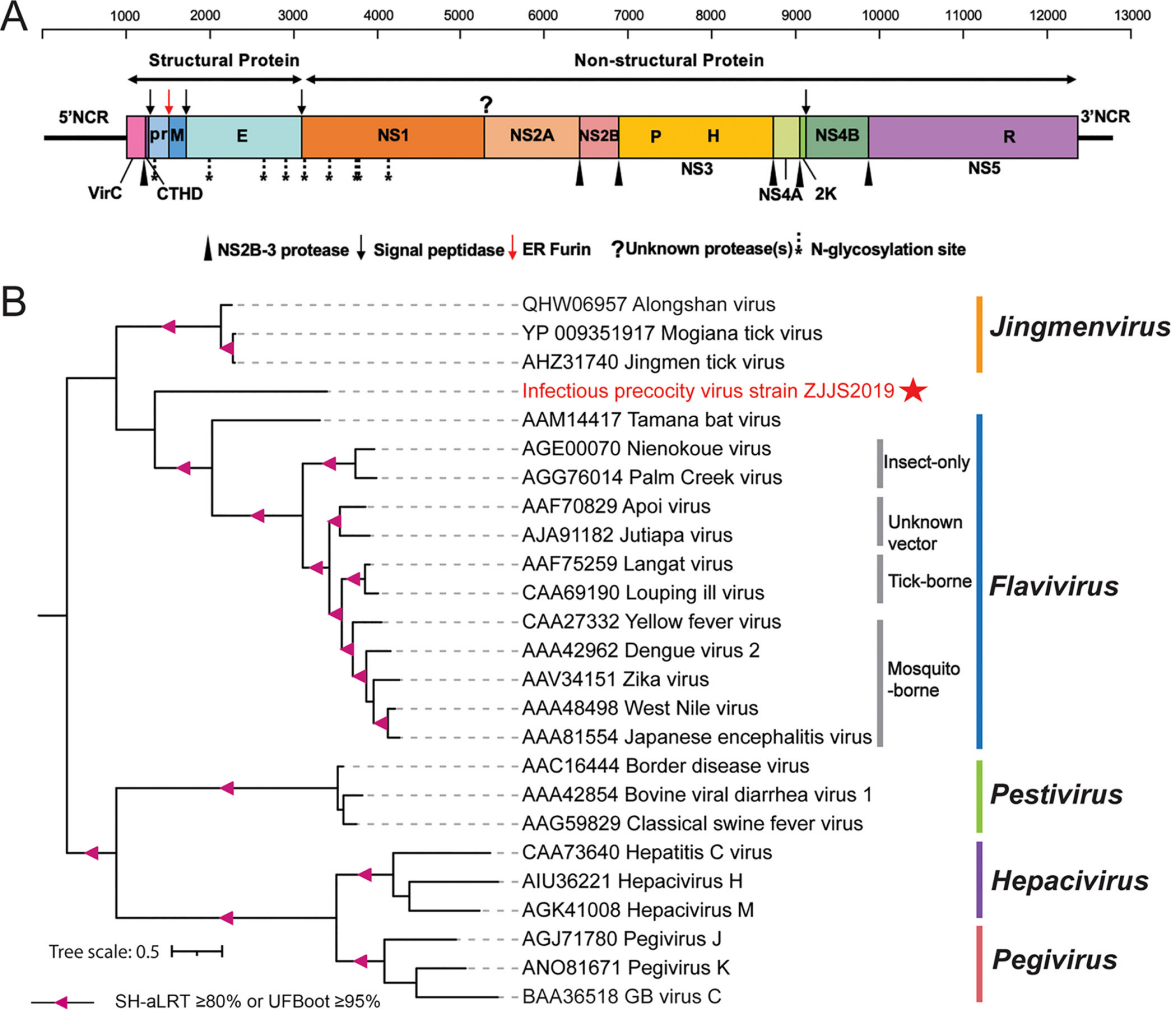
Genome structure and phylogenetic analysis of a novel flavivirus discovered. (A) Genome architecture of IPV strain ZJJS2019. P, H, and R indicate the localization of the NS3 protease, the NS3 RNA helicase, and the NS5 RdRP domains, respectively. (B) Phylogenetic tree of NS5 protein (~420 amino acids) of the family *Flaviviridae*. The triangle indicates a node SH-aLRT value of ≥80% or UFBoot value of ≥95%. The virus described in this study is marked with a solid red asterisk. The scale bar represents 0.5 amino acid substitution per site.

The *Flaviviridae* are a family of small enveloped viruses with positive-sense RNA genomes of ~9 to 13 kb that encode a large polyprotein precursor. For the maturation of flavivirus particles, the polyprotein precursor is cleaved into structural proteins (capsid protein [C], premembrane protein [prM], and envelope protein [E]) and also nonstructural (NS) proteins (NS1, NS2A, NS2B, NS3, NS4A, 2K, NS4B, and NS5) (39). Here, one 12,594-bp viral contig had 98.8% nucleotide identity with the previously identified infectious precocity virus (IPV) (28), tentatively named Infectious precocity virus strain ZJJS2019 (IPV strain ZJJS2019). IPV strain ZJJS2019 was obtained only from GRD-affected prawns, with high relative abundance (up to 91,141 RPM) (Table 1). The genome of IPV strain ZJJS2019 presented the same site of programmed −1 ribosomal frameshifting (−1PRF) to produce a single polyprotein (3,707 amino acids) (28), sharing 99.62% amino acid identity across the polyprotein with IPV strain ZJJS2019 (Fig. 8A and Table 1). Based on the sequence alignment of homologous sequences by MUSCLE, potential polyprotein cleavage sites of the IPV strain ZJJS2019 were identified (Fig. 8A and Table S4). The order of proteins encoded in the IPV strain ZJJS2019 polyprotein is 5′-VirC-CTHD-pr-M-E-NS1-NS2A-NS2B-NS3-NS4A-2K-NS2B-NS5-3′, which is typical of flaviviruses (Fig. 8A) (39). Phylogenetic analysis of conserved amino acid sequences in the RdRp (NS5) demonstrated that IPV strain ZJJS2019 has the closest evolutionary relationship to a group of Jingmenvirus and Tamana bat virus (TABV) isolates belonging to the genus *Flavivirus* (Fig. 8B). TABV is currently listed as a potential member of the genus *Flavivirus* but sufficiently distinct to potentially merit assignment into a new genus (40). Therefore, we propose that IPV strain ZJJS2019 could be considered a new genus in the family *Flaviviridae* as described by Dong et al. (28).

## DISCUSSION

Our findings support previous observations that crustaceans’ and other invertebrates’ viromes have harbored a broad diversity of viruses of the order *Picornavirales*. Shi et al. (5) found that numerous and divergent picorna-like viruses were ubiquitous in invertebrate species. In addition, previous virome analyses of honeybees and other insect pollinators also observed that *Picornavirale*s was the dominant group (41–43). Furthermore, 46 picorna-like virus sequences were recently identified in multiple marine invertebrate species from three different seas (the South China Sea, the East China Sea, and the Yellow Sea) in China (44). Although the pathogenicity of picorna-like viruses that were mostly discovered from invertebrate species without any obvious clinical symptoms is still unclear, several picornaviruses are known to be highly pathogenic to beneficial arthropods. For instance, a variety of important honeybee pathogenic viruses, such as deformed wing virus (DWV) (45, 46), acute bee paralysis virus (ABPV) (47), and black queen cell virus (BQCV) (48), caused widespread disappearance and death of honeybee populations (49). Also, three dicistroviruses were implicated as the causative pathogens of disease outbreaks among some crustaceans. Taura syndrome virus (TSV), infecting several penaeid shrimp species, has ranged from 40% to >90% in cultured populations of postlarval (PL), juvenile, and subadult *P. vannamei* (50, 51). Mud crab dicistrovirus (MCDV) is known to cause sudden and massive mortalities in crabs (52). Furthermore, Macrobrachium rosenbergii Taihu virus (MrTV) was confirmed as the causative agent of the larval mortality syndrome in *M. rosenbergii* (22). A shred of growing evidence has indicated that viruses of the order *Picornavirales* might pose high risks to many hosts. The potential risks of these picorna-like viruses in *M. rosenbergii* should be investigated further.

Virome comparison between diseased and healthy prawns could help to find potential pathogens for hosts. Fifty-one viral contigs were shared among two groups from different ponds, likely indicating the core virome of *M. rosenbergii*, as was recently observed for brown shrimp (9), rodents (6), and mosquitoes (53–55). However, the differences in the viral relative abundance were noted between diseased and normal prawns, indicating that some viruses become more abundant in occurrences or outbreaks of diseases, and they might be potential high-risk viruses for *M. rosenbergii*. MRV1, a novel Solinvi-like virus, was the most abundant virus in both diseased and normal prawns. Some members of *Solinviviridae* are pathogenic viruses for ants and some arthropods, like Solenopsis invicta virus 3 (SINV-3)-infected Solenopsis invicta queens, resulting in significant reductions in fecundity and body weight and slower growth of ant colonies (56). Acyrthosiphon pisum virus (APV) has been reported to reduce the growth of pea aphids and increase the time to reach maturity (57). So, this indicated that MRV1 might pose a potential risk to *M. rosenbergii* due to the high concentrations. It would be necessary to further investigate the pathogenicity of MRV1 in *M. rosenbergii*.

The second potentially dangerous virus could be the IPV strain ZJJS2019, belonging to the family *Flaviviridae*, which was the next most abundant virus and was present only in the diseased pool. Many flaviviruses are known to be important human and veterinary pathogens, including yellow fever virus, dengue virus, hepatitis C virus, Japanese encephalitis virus, and classical swine fever virus, causing fatal hemorrhagic fever, chronic liver or neurological disease in humans, and economically important diseases in domestic or wild animals (39). IPV strain ZJJS2019 is the first flavivirus associated with *M. rosenbergii*, expanding the host range infecting the invertebrate species, especially the crustaceans, over what is currently reported (58). A previous study indicated that IPV strain ZJJS2019 was associated with sexual precocity in *M. rosenbergii* (28). Furthermore, Parry and Asgari demonstrated that the horizontal transmission of dual host invertebrate-vertebrate flaviviruses was present between crabs and sharks and offered the potential insights to explore the origin or emergence of terrestrial vector-borne flaviviruses (58). Thus, future studies need to investigate the pathogenicity and host range of IPV strain ZJJS2019 from invertebrates to vertebrates to reveal the epidemiology of IPV strain ZJJS2019 and elucidate potential cross-species transmission threats.

Furthermore, some novel viral sequences related to already known crustacean viruses in the phylogenetic trees might pose potential risks to *M. rosenbergii* aquaculture. For instance, MRV9 was assigned to the same family, *Dicistroviridae*, as TSV, MCDV, and MrTV, which were already known to cause massive mortalities in crustacean species. MRV9 was the third most abundant virus in pool D and at much higher abundance than in pool H, suggesting the potential threat for *M. rosenbergii*. Although Dong et al. (28) showed that the healthy *M. rosenbergii* prawns infected by a viral preparation extracted from “iron prawn syndrome” (IPS)-affected prawns could show clinical signs of growth cessation and sexual precocity, and all infected prawns were positive for IPV, molecular diagnostics were not performed to detect other potentially high-risk viruses in the infected prawns. Except for IPV, MRV1 and MRV9 also might be associated with GRD and were more abundant in diseased prawns than normal ones, and relatedness to the known pathogenic viruses. The potential roles of the high-risk viruses in different disease states need to be confirmed by more molecular techniques and further monitoring of their prevalence in emerging infectious diseases in *M. rosenbergii*.

Cross-species transmission (i.e., horizontal virus transfer) in animal and plant populations may lead to emerging infections in the recipient host (38, 59). Here, the nearly complete genome of MRV15 shared >99% amino acid identity with two viruses found in the plants Trichosanthes kirilowii and Forsythia suspensa (38). The two plants are important medicinal plants, widely distributed in China (60). Forsythia suspensa plant extracts have proved to be potential substitutes for antibiotics due to their anti-inflammatory antioxidant and intestinal barrier function in aquatic animals, which are as feed additives used in aquaculture (61, 62). We detected MRV15 in *M. rosenbergii* with high prevalence, which was consistent with a high viral abundance in metatranscriptome sequencing. In addition, in the *Picornavales* phylogenetic tree, MRV15 formed a sister clade with unclassified picorna-like viruses infecting arthropods in a previous study (5). These results indicated that *M. rosenbergii* was the host of MRV15, and cross-species transmission might have occurred between crustaceans and plants. Previous studies also reported examples of viruses with host ranges spanning both the plant and animal kingdoms. Li et al. (63) proved that a plant-pathogenic virus, tobacco ringspot virus (TRSV) of the *Secoviridae* family, could replicate and produce virions in honeybees, (Apis mellifera), resulting in systemic infections throughout the body. Furthermore, some viruses known to infect arthropods can also infect plants. For instance, Flock House virus (FHV), an insect virus belonging to the *Nodaviridae* family, has been shown to replicate in plants and mammalian and yeast cells (64). Emerging infectious diseases resulting from cross-species transmission often threaten host health. These viruses should be of concern due to their invasiveness and ability to spread among different species (65). Thus, the pathogenicity and transmissibility of MRV15 in *M. rosenbergii* deserve further investigation.

Different environments might affect the viral composition of hosts. Zhang et al. found that multiple marine invertebrate species from three seas in China seemingly contained sea-specific virus groups (44). In addition, some differences in the viral composition and abundance in sediments were noted with the culture species added to different mariculture systems (66). A large-scale virome project involving more extensive ranges and more organisms would more significantly capture the viral diversity of hosts and provide more detailed information about the virome heterogeneity and connectivity of different environments. The GRD-affected prawns contained a higher viral abundance than the normal groups, while the normal group exhibited higher viral diversity. This might be explained by the replications of some high-risk viruses being enhanced in the diseased state of *M. rosenbergii*, resulting in a decrease in viral diversity. However, our study remains preliminary due to the limitation of sample size, and the topic still needs to be further explored with a greater diversity of viromes in *M. rosenbergii* within a larger geographic range and a longer time.

In conclusion, this study has significantly expanded our understanding of the viral diversity of *M. rosenbergii*, especially numerous viruses from the order *Picornavirales*. The characterization of viruses in *M. rosenbergii* will facilitate our knowledge of virus evolution and cross-species transmission and reduce the potential threat of cross-species infections. Comparison of the viral composition and abundance in the GRD-affected prawns with those in normal prawns will help to identify potentially high-risk viruses and provide valuable information for future disease surveillance and prevention of *M. rosenbergii*.

## MATERIALS AND METHODS

### Sample collection and preparation.

In August 2019, giant freshwater prawns (Macrobrachium rosenbergii) stocked for 70 to 80 days were collected from farming ponds in Jiashan County, Zhejiang Province, China. The diseased and normal prawns collected from different ponds in the study originated from the same hatchery with the same genetic background. Fifteen GRD-affected giant freshwater prawns, with body lengths ranging from 6.0 to 6.8 cm, were collected from diseased farm ponds with typical GRD. The clinical signs of prawns with GRD included smaller body size, hard shell, and precocious puberty. Fifteen healthy ones were acquired from the farm ponds without GRD, with similar seedling release times, appropriate feeding, and management methods. The body lengths of healthy ones were 8.2 to 9.0 cm. The gill, hepatopancreases, and muscle tissues of all prawns were immediately dissected and stored separately in RNAlater before transferring them to a freezer set at −80°C.

### Virus enrichment, RNA library construction, and sequencing.

To increase the likelihood of virus discovery, the gill, hepatopancreases, and muscle tissues from 15 prawns with the same health condition were pooled in equal weight (~50 mg), resulting in two pools—the diseased (D) pool and the healthy (H) pool, respectively. The sample pools were homogenized with phosphate-buffered saline (PBS) (10% mass/vol) in a Lu kα sample freezing grinder (LUKYM-I) for 3 min at 60 Hz and 4°C, frozen, and thawed three times on dry ice. The suspension of each pool was subjected to centrifugation (10 min at 12,000 × *g*). The supernatant was filtered through a 0.45-μm-pore filter (Millipore) to remove eukaryotic and bacterial cell-sized particles. All filtrates were concentrated using ultracentrifugation to enrich the viral particles. RNA was extracted using the QiaAmp mini-viral RNA kit (Qiagen, Germany) and quantified using Qubit (Thermo Fisher, USA). To eliminate possible reagent contaminants, we included sterile water as a control (67). The rRNA was removed for library construction by the NEBNext rRNA depletion kit (NEB, USA). Sequencing libraries were generated using RNA library prep kit for Illumina (NEB, USA) following the manufacturer’s recommendations. Then, the 150-bp paired-end sequencing of the RNA libraries was performed using the Illumina NovaSeq platform (Illumina, USA) at Novogene (Beijing, China).

### Sequence assembly and virus discovery.

For each library, raw reads were filtered and quality-trimmed with fastp (version 0.21.0) (68), and clean reads were *de novo* assembled by using Trinity (version 2.12.0) (69) with default settings. Contigs longer than 500 bp from two pools were clustered for redundancy at 95% nucleotide identity over 80% of the length using CD-HIT (version 4.8.1) (54, 70). Representative contigs were annotated against the NCBI nonredundant protein (nr) database using DIAMOND (blastx, version 2.0.11) (71), setting an E value of 1E−5 to remove false-positive results. We filtered the blastx results with the keyword “Viruses.” To remove false positives (e.g., endogenous viral elements and nonviral host sequences), all putative viral contigs were compared against the host reference genome of *M. rosenbergii* (an in-house *M. rosenbergii* genome) and nonredundant nucleotide (nt) database with an E value threshold of 1E−5 using the blastn algorithm. The taxonomic lineage information of viral contigs was collected based on the blastx results; viral contigs that are related to virus families well known to infect plants were excluded (72).

To confirm the relative abundance of viral contigs, clean reads were mapped back to the nonredundant contigs using Bowtie2 (version 2.4.2) (73) and inspected using Integrative Genomics Viewer (IGV [version 2.5.0]) (74). To identify and eliminate possible contamination, we used the following steps to exclude false positives of these viral contigs suggested by previous study (75). The relative abundance of each viral contig was quantified as the number of mapped reads per million total reads (RPM), and we set RPM at ≥1 and the number of identified reads at ≥10 for a positive virus hit. To reduce the cross-contamination due to index hopping, the reads of viral contigs in the low-abundance library were removed if the abundance of a viral contig in one library was <0.1% of the higher abundance library. Rarefaction analyses were conducted to visualize the difference in the viral contigs composition (76).

### PCR confirmation and prevalence investigation.

To confirm the presence of a subset of the newly identified viruses (see Table S2 in the supplemental material), nested reverse transcription-PCR (RT-PCR) was performed (Fig. S3) using specific primer pairs designed according to assembled viral contigs (Table S4). The target PCR products were visualized on 1.5% agarose gel and were validated by Sanger sequencing (Fig. S4). In order to investigate the prevalence of MRV15, 23 prawns with typical GRD status collected from Huzhou, Zhejiang Province, in 2020 were tested for MRV15 by nested RT-PCR assay, and RNase-free water was used as a negative control. PCR products were confirmed using Sanger sequencing.

### Virus genome annotation.

The potential open reading frames of the obtained viral contigs were predicted with the NCBI Open Reading Frame Finder (https://www.ncbi.nlm.nih.gov/orffinder/), based on two criteria: (i) the minimal ORF length was longer than 100 amino acids, and (ii) a nested ORF was ignored unless it had a homolog in the closely related virus. To characterize the functional domains, predicted viral ORFs were analyzed using the NCBI Conserved Domain Search (https://www.ncbi.nlm.nih.gov/Structure/cdd/wrpsb.cgi/) (77) together with InterPro (http://www.ebi.ac.uk/interpro/) (78) with default settings. Prediction of putative polyprotein cleavage sites of the flaviviruses was conducted as previously described (58). Briefly, the prediction of transmembrane helices of the polyprotein was performed using TMHMM version 2.0 (https://services.healthtech.dtu.dk/service.php?TMHMM-2.0/). To identify signal peptides, a 70-amino-acid sliding window of the polyprotein was assessed with the SignalP version 6.0 webserver (https://services.healthtech.dtu.dk/service.php?SignalP-6.0/). In addition, the cleavage sites were predicted using the NetChop 3.1 server (https://services.healthtech.dtu.dk/service.php?NetChop-3.1/). The furin cleavage sites were identified using the ProP 1.0 webserver (https://services.healthtech.dtu.dk/service.php?ProP-1.0/). Putative N-glycosylation sites were predicted using the NetNGlyc 1.0 server (https://services.healthtech.dtu.dk/service.php?NetNGlyc-1.0/).

### Phylogenetic analysis.

We selected representative viral sequences proposed by the International Committee on Taxonomy of Viruses (ICTV [https://ictv.global/report/]) to determine the evolutionary history of 16 nearly complete RNA viruses. Their closest viral relatives are based on the search in blastp (https://blast.ncbi.nlm.nih.gov/Blast.cgi/). The viral amino acid sequences were then aligned using MEGA-X version 10.2.6, employing the MUSCLE algorithm (79). All alignments were trimmed with TBtools version 1.09852 (80) utilizing the TrimAL program (81). The trimmed alignments were then checked manually. Maximum likelihood phylogenetic trees were reconstructed with the best-fit model of amino acid substitution determined using IQ-TREE version 1.6.12 (82). Branch support was accessed using the Shimodaira-Hasegawa-like approximate likelihood ratio test (SH-aLRT) and ultrafast bootstrap (UFBoot). The trees were visualized with iTOL (https://itol.embl.de/) (83) and refined by Adobe Photoshop CC version 19.1.6.

### Data availability.

The raw sequence reads we analyzed in this study have been deposited in the SRA databases under the project accession no. PRJNA832012. All viral sequences with nearly complete genomes were deposited in the GenBank database under accession no. ON382564 to ON382579.

## References

[1] Zhang W, Yang S, Shan T, Hou R, Liu Z, Li W, Guo L, Wang Y, Chen P, Wang X, Feng F, Wang H, Chen C, Shen Q, Zhou C, Hua X, Cui L, Deng X, Zhang Z, Qi D, Delwart E. 2017. Virome comparisons in wild-diseased and healthy captive giant pandas. Microbiome 5:90. doi:10.1186/s40168-017-0308-0. 28780905 PMC5545856

[2] Sun Y, Qu Y, Yan X, Yan G, Chen J, Wang G, Zhao Z, Liu Y, Tu C, He B. 2022. Comprehensive evaluation of RNA and DNA viromic methods based on species richness and abundance analyses using marmot rectal samples. mSystems 7:e00430-22. doi:10.1128/msystems.00430-22. 35862817 PMC9426427

[3] Chong R, Shi M, Grueber CE, Holmes EC, Hogg CJ, Belov K, Barrs VR. 2019. Fecal viral diversity of captive and wild Tasmanian devils characterized using virion-enriched metagenomics and metatranscriptomics. J Virol 93:e00205-19. doi:10.1128/JVI.00205-19. 30867308 PMC6532096

[4] He WT, Hou X, Zhao J, Sun J, He H, Si W, Wang J, Jiang Z, Yan Z, Xing G, Lu M, Suchard MA, Ji X, Gong W, He B, Li J, Lemey P, Guo D, Tu C, Holmes EC, Shi M, Su S. 2022. Virome characterization of game animals in China reveals a spectrum of emerging pathogens. Cell 185:1117–1129.e8. doi:10.1016/j.cell.2022.02.014. 35298912 PMC9942426

[5] Shi M, Lin XD, Tian JH, Chen LJ, Chen X, Li CX, Qin XC, Li J, Cao JP, Eden JS, Buchmann J, Wang W, Xu J, Holmes EC, Zhang YZ. 2016. Redefining the invertebrate RNA virosphere. Nature 540:539–543. doi:10.1038/nature20167. 27880757

[6] Wu ZQ, Han YL, Liu B, Li HY, Zhu GJ, Latinne A, Dong J, Sun LL, Su HX, Liu LG, Du J, Zhou SY, Chen MX, Kritiyakan A, Jittapalapong S, Chaisiri K, Buchy P, Duong V, Yang JA, Jiang JY, Xu X, Zhou HN, Yang F, Irwin DM, Morand S, Daszak P, Wang JW, Jin Q. 2021. Decoding the RNA viromes in rodent lungs provides new insight into the origin and evolutionary patterns of rodent-borne pathogens in mainland Southeast Asia. Microbiome 9:18. doi:10.1186/s40168-020-00965-z. 33478588 PMC7818139

[7] Geoghegan JL, Di Giallonardo F, Wille M, Ortiz-Baez AS, Costa VA, Ghaly T, Mifsud JCO, Turnbull OMH, Bellwood DR, Williamson JE, Holmes EC. 2021. Virome composition in marine fish revealed by meta-transcriptomics. Virus Evol 7:veab005. doi:10.1093/ve/veab005. 33623709 PMC7887440

[8] Costa VA, Mifsud JCO, Gilligan D, Williamson JE, Holmes EC, Geoghegan JL. 2021. Metagenomic sequencing reveals a lack of virus exchange between native and invasive freshwater fish across the Murray-Darling Basin, Australia. Virus Evol 7:veab034. doi:10.1093/ve/veab034. 34017611 PMC8121191

[9] Van Eynde B, Christiaens O, Delbare D, Shi C, Vanhulle E, Yinda CK, Matthijnssens J, Smagghe G. 2020. Exploration of the virome of the European brown shrimp (*Crangon crangon*). J Gen Virol 101:651–666. doi:10.1099/jgv.0.001412. 32391748

[10] Bačnik K, Kutnjak D, Černi S, Bielen A, Hudina S. 2021. Virome analysis of signal crayfish (*Pacifastacus leniusculus*) along its invasion range reveals diverse and divergent RNA viruses. Viruses 13:2259. doi:10.3390/v13112259. 34835065 PMC8624288

[11] Orosco FL, Lluisma AO. 2017. Variation in virome diversity in wild populations of *Penaeus monodon* (Fabricius 1798) with emphasis on pathogenic viruses. Virusdisease 28:262–271. doi:10.1007/s13337-017-0389-1. 29291212 PMC5685000

[12] Rosani U, Gerdol M. 2017. A bioinformatics approach reveals seven nearly-complete RNA-virus genomes in bivalve RNA-seq data. Virus Res 239:33–42. doi:10.1016/j.virusres.2016.10.009. 27769778

[13] Hewson I, Aquino CA, DeRito CM. 2020. Virome variation during sea star wasting disease progression in *Pisaster ochraceus* (Asteroidea, Echinodermata). Viruses 12:1332. doi:10.3390/v12111332. 33233680 PMC7699681

[14] Fu X, Luo M, Zheng G, Liang H, Liu L, Lin Q, Niu Y, Luo X, Li N. 2022. Determination and characterization of a novel birnavirus associated with massive mortality in largemouth bass. Microbiol Spectr 10:e01716-21. doi:10.1128/spectrum.01716-21. 35319246 PMC9045220

[15] Hooper C, Debnath PP, Biswas S, van Aerle R, Bateman KS, Basak SK, Rahman MM, Mohan CV, Islam HMR, Ross S, Stentiford GD, Currie D, Bass D. 2020. A novel RNA virus, *Macrobrachium rosenbergii* golda virus (MrGV), linked to mass mortalities of the larval giant freshwater prawn in Bangladesh. Viruses 12:1120. doi:10.3390/v12101120. 33023199 PMC7601004

[16] Naveen Kumar S, Rai P, Karunasagar I, Karunasagar I. 2020. Genomic and antibody-based assays for the detection of Indian strains of *Macrobrachium rosenbergii* nodavirus and extra small virus associated with white tail disease of *Macrobrachium rosenbergii*. Virusdisease 31:459–469. doi:10.1007/s13337-020-00641-8. 33381620 PMC7749019

[17] Yang GL, Frinsko M, Chen XF, Wang JY, Hu G, Gao Q. 2012. Current status of the giant freshwater prawn (*Macrobrachium rosenbergii*) industry in China, with special reference to live transportation. Aquac Res 43:1049–1055. doi:10.1111/j.1365-2109.2011.03009.x.

[18] BOF, NFTEC, CSF. 2021. China fishery statistical yearbook 2021, p 24. China Agriculture Press, Beijing, China.

[19] Pillai D, Bonami JR. 2012. A review on the diseases of freshwater prawns with special focus on white tail disease of *Macrobrachium rosenbergii*. Aquac Res 43:1029–1037. doi:10.1111/j.1365-2109.2011.03061.x.

[20] Arcier JM, Herman F, Lightner DV, Redman RM, Mari J, Bonami JR. 1999. A viral disease associated with mortalities in hatchery-reared postlarvae of the giant freshwater prawn *Macrobrachium rosenbergii*. Dis Aquat Org 38:177–181. doi:10.3354/dao038177.

[21] Hsieh CY, Chuang PC, Chen LC, Tu C, Chien MS, Huang KC, Kao HF, Tung MC, Tsai SS. 2006. Infectious hypodermal and haematopoietic necrosis virus (IHHNV) infections in giant freshwater prawn, *Macrobrachium rosenbergii*. Aquaculture 258:73–79. doi:10.1016/j.aquaculture.2006.04.007.

[22] Pan X, Cao Z, Yuan J, Shi Z, Yuan X, Lin L, Xu Y, Yao J, Hao G, Shen J. 2016. Isolation and characterization of a novel dicistrovirus associated with mortalities of the great freshwater prawn, *Macrobrachium rosenbergii*. Int J Mol Sci 17:204. doi:10.3390/ijms17020204. 26848659 PMC4783938

[23] An ZH, Sun LS, Chen JY. 2014. Investigation on the causes of the phenomenon of “iron shell” of *Macrobrachium rosenbergii*. Sci Fish Farming 1:56–58. doi:10.14184/j.cnki.issn1004-843x.2014.01.034.

[24] Sun H. 2017. The contents of relative hormones and their gene expressions for growth retardant *Macrobrachium rosenbergii*. Yangzhou University, Yangzhou, China.

[25] Ying N, Wang Y, Song X, Qin B, Wu Y, Yang L, Fang W. 2022. Transcriptome analysis of *Macrobrachium rosenbergii*: identification of precocious puberty and slow-growing information. J Invertebr Pathol 190:107752. doi:10.1016/j.jip.2022.107752. 35367462

[26] Dong XH, Ma JS, CHen ZX. 2015. Comprehensive prevention and control measures for “slow growth” of *Macrobrachium rosenbergii*. Sci Fish Farming 3:61. doi:10.14184/j.cnki.issn1004-843x.2015.04.037.

[27] Zhou JM, Dai XL, Jiang F, Ding FJ. 2017. The preliminary analysis of the reasons for the poor growth of *Macrobrachium rosenbergii* in pond. J Shanghai Ocean Univ 26:854–861.

[28] Dong X, Wang G, Hu T, Li J, Li C, Cao Z, Shi M, Wang Y, Zou P, Song J, Gao W, Meng F, Yang G, Tang KFJ, Li C, Shi W, Huang J. 2021. A novel virus of *Flaviviridae* associated with sexual precocity in *Macrobrachium rosenbergii*. mSystems 6:e00003-21. doi:10.1128/mSystems.00003-21. 34100644 PMC8269200

[29] Gao XJ, Zhou YF, Zhu XH, Tang HY, Li XX, Jiang Q, Wei WH, Zhang XJ. 2021. Enterobacter cloacae: a probable etiological agent associated with slow growth in the giant freshwater prawn *Macrobrachium rosenbergii*. Aquaculture 530:735826. doi:10.1016/j.aquaculture.2020.735826.

[30] Wang G, Dong X, Wang Y, Guo X, Wang D, Meng F, Huang J. 2021. Study on the eyestalk transcriptome of *Macrobrachium rosenbergii* suffering from iron prawn syndrome. Prog Fishery Sci 42:1–12.

[31] Zhang YY, Wang LF, Zhuang H, Li XX, Gao XJ, An ZH, Liu XD, Yang H, Wei WZ, Zhang XJ. 2019. Excessive use of enrofloxacin leads to growth inhibition of juvenile giant freshwater prawn *Macrobrachium rosenbergii*. Ecotoxicol Environ Saf 169:344–352. doi:10.1016/j.ecoenv.2018.11.042. 30458401

[32] Yuan R, ZHang ZH, Chen H, Fang P, Chen J, Liu XM, Wu YF, Wang JJ. 2017. Phenomenon and research progress on prevention and control of “Iron Shell” in giant freshwater prawn *Macrobrachium rosenbergii*. Fish Sci 36:383–390.

[33] Brown K, Olendraite I, Valles SM, Firth AE, Chen Y, Guérin DMA, Hashimoto Y, Herrero S, de Miranda JR, Ryabov E, ICTV Report Consortium. 2019. ICTV virus taxonomy profile: *Solinviviridae*. J Gen Virol 100:736–737. doi:10.1099/jgv.0.001242. 30835197 PMC12662029

[34] Olendraite I, Brown K, Valles SM, Firth AE, Chen Y, Guérin DMA, Hashimoto Y, Herrero S, de Miranda JR, Ryabov E, ICTV Report Consortium. 2019. ICTV virus taxonomy profile: *Polycipiviridae*. J Gen Virol 100:554–555. doi:10.1099/jgv.0.001241. 30835199 PMC7011767

[35] Viljakainen L, Holmberg I, Abril S, Jurvansuu J. 2018. Viruses of invasive Argentine ants from the European Main supercolony: characterization, interactions and evolution. J Gen Virol 99:1129–1140. doi:10.1099/jgv.0.001104. 29939128

[36] Lang AS, Vlok M, Culley AI, Suttle CA, Takao Y, Tomaru Y, ICTV Report Consortium. 2021. ICTV virus taxonomy profile: *Marnaviridae* 2021. J Gen Virol 102:001633. doi:10.1099/jgv.0.001633. 34356002 PMC8513639

[37] Valles SM, Chen Y, Firth AE, Guérin DMA, Hashimoto Y, Herrero S, de Miranda JR, Ryabov E, ICTV Report Consortium. 2017. ICTV virus taxonomy profile: *Dicistroviridae*. J Gen Virol 98:355–356. doi:10.1099/jgv.0.000756. 28366189 PMC5797946

[38] Yang S, Shan T, Wang Y, Yang J, Chen X, Xiao Y, You Z, He Y, Zhao M, Lu J, Yang Z, Dai Z, Liu Q, Yao Y, Lu X, Li H, Zhou R, Li W, Zhou C, Wang X, Shen Q, Xu H, Deng X, Delwart E, Zhang W. 2020. Virome of riverside phytocommunity ecosystem of an ancient canal. Research Square https://www.researchsquare.com/article/rs-25620/v1.

[39] Simmonds P, Becher P, Bukh J, Gould EA, Meyers G, Monath T, Muerhoff S, Pletnev A, Rico-Hesse R, Smith DB, Stapleton JT, ICTV Report Consortium. 2017. ICTV virus taxonomy profile: *Flaviviridae*. J Gen Virol 98:2–3. doi:10.1099/jgv.0.000672. 28218572 PMC5370391

[40] de Lamballerie X, Crochu S, Billoir F, Neyts J, de Micco P, Holmes EC, Gould EA. 2002. Genome sequence analysis of Tamana bat virus and its relationship with the genus *Flavivirus*. J Gen Virol 83:2443–2454. doi:10.1099/0022-1317-83-10-2443. 12237426

[41] Li NN, Huang YZ, Li W, Xu SF. 2022. Virome analysis reveals diverse and divergent RNA viruses in wild insect pollinators in Beijing, China. Viruses 14:227. doi:10.3390/v14020227. 35215821 PMC8877953

[42] Remnant EJ, Baty JW, Bulgarella M, Dobelmann J, Quinn O, Gruber MAM, Lester PJ. 2021. A diverse viral community from predatory wasps in their native and invaded range, with a new virus infectious to honey bees. Viruses 13:1431. doi:10.3390/v13081431. 34452301 PMC8402789

[43] Roberts JMK, Anderson DL, Durr PA. 2018. Metagenomic analysis of Varroa-free Australian honey bees (*Apis mellifera)* shows a diverse *Picornavirales v*irome. J Gen Virol 99:818–826. doi:10.1099/jgv.0.001073. 29749926

[44] Zhang YY, Chen YC, Wei XM, Cui J. 2022. Viromes in marine ecosystems reveal remarkable invertebrate RNA virus diversity. Sci China Life Sci 65:426–437. doi:10.1007/s11427-020-1936-2. 34156600

[45] Bowen-Walker PL, Martin SJ, Gunn A. 1999. The transmission of deformed wing virus between honeybees (*Apis mellifera L.*) by the ectoparasitic mite Varroa jacobsoni Oud. J Invertebr Pathol 73:101–106. doi:10.1006/jipa.1998.4807. 9878295

[46] Martin SJ, Brettell LE. 2019. Deformed wing virus in honeybees and other insects. Annu Rev Virol 6:49–69. doi:10.1146/annurev-virology-092818-015700. 31185188

[47] Chanpanitkitchote P, Chen YP, Evans JD, Li WF, Li JH, Hamilton M, Chantawannakul P. 2018. Acute bee paralysis virus occurs in the Asian honey bee Apis cerana and parasitic mite Tropilaelaps mercedesae. J Invertebr Pathol 151:131–136. doi:10.1016/j.jip.2017.11.009. 29158015

[48] Yang Q, Zhang J, Song Z, Zheng Y, Wang X, Sui J, Wang Z, Mou J. 2015. Research progress in black queen cell virus causing disease. Bing Du Xue Bao 31:318–325. 26470541

[49] Ullah A, Tlak Gajger I, Majoros A, Dar SA, Khan S, Haleem Shah A, Nasir Khabir M, Hussain R, Khan HU, Hameed M, Anjum SI, Kalimullah. 2021. Viral impacts on honey bee populations: a review. Saudi J Biol Sci 28:523–530. doi:10.1016/j.sjbs.2020.10.037. 33424335 PMC7783639

[50] Hasson KW, Lightner DV, Mohney LL, Redman RM, Poulos BT, White BM. 1999. Taura syndrome virus (TSV) lesion development and the disease cycle in the Pacific white shrimp *Penaeus vannamei*. Dis Aquat Org 36:81–93. doi:10.3354/dao036081.

[51] Lightner DV. 2011. Virus diseases of farmed shrimp in the Western Hemisphere (the Americas): a review. J Invertebr Pathol 106:110–130. doi:10.1016/j.jip.2010.09.012. 21215359 PMC7172539

[52] Guo ZX, He JG, Xu HD, Weng SP. 2013. Pathogenicity and complete genome sequence analysis of the mud crab dicistrovirus-1. Virus Res 171:8–14. doi:10.1016/j.virusres.2012.10.002. 23073178

[53] Coatsworth H, Bozic J, Carrillo J, Buckner EA, Rivers AR, Dinglasan RR, Mathias DK. 2022. Intrinsic variation in the vertically transmitted core virome of the mosquito *Aedes aegypti*. Mol Ecol 31:2545–2561. doi:10.1111/mec.16412. 35229389

[54] Shi C, Beller L, Deboutte W, Yinda KC, Delang L, Vega-Rúa A, Failloux A-B, Matthijnssens J. 2019. Stable distinct core eukaryotic viromes in different mosquito species from Guadeloupe, using single mosquito viral metagenomics. Microbiome 7:121. doi:10.1186/s40168-019-0734-2. 31462331 PMC6714450

[55] Shi CY, Zhao L, Atoni E, Zeng WF, Hu XM, Matthijnssens J, Yuan ZM, Xia H. 2020. Stability of the virome in lab- and field-collected Aedes albopictus mosquitoes across different developmental stages and possible core viruses in the publicly available virome data of Aedes mosquitoes. mSystems 5:e00640-20. doi:10.1128/mSystems.00640-20. 32994288 PMC7527137

[56] Valles SM, Porter SD, Firth AE. 2014. Solenopsis invicta virus 3: pathogenesis and stage specificity in red imported fire ants. Virology 460–461:66–71. doi:10.1016/j.virol.2014.04.026. 25010271

[57] van den Heuvel JF, Hummelen H, Verbeek M, Dullemans AM, van der Wilk F. 1997. Characteristics of Acyrthosiphon pisum virus, a newly identified virus infecting the pea aphid. J Invertebr Pathol 70:169–176. doi:10.1006/jipa.1997.4691. 9367722

[58] Parry R, Asgari S. 2019. Discovery of novel crustacean and cephalopod flaviviruses: insights into the evolution and circulation of flaviviruses between marine invertebrate and vertebrate hosts. J Virol 93:e00432-19. doi:10.1128/JVI.00432-19. 31068424 PMC6600200

[59] Dolja VV, Koonin EV. 2018. Metagenomics reshapes the concepts of RNA virus evolution by revealing extensive horizontal virus transfer. Virus Res 244:36–52. doi:10.1016/j.virusres.2017.10.020. 29103997 PMC5801114

[60] NPC. 2020. Pharmacopoeia of People's Republic of China herbal medicine. Chemical Industry Press, Beijing, China.

[61] Xie JJ, Chen X, Guo TY, Xie SW, Fang HH, Liu ZL, Zhang YM, Tian LX, Liu YJ, Niu J. 2018. Dietary values of *Forsythia suspensa* extract in *Penaeus monodon* under normal rearing and *Vibrio parahaemolyticus* 3HP (VP(3HP)) challenge conditions: effect on growth, intestinal barrier function, immune response and immune related gene expression. Fish Shellfish Immunol 75:316–326. doi:10.1016/j.fsi.2018.02.030. 29454898

[62] Lu XP, Zhang XX, Wang L, Li JT, Zhang ZL, Zheng PH, Xian JA. 2020. Advances in the application of plant extracts in aquatic animals. Feed Res 5:134–138.

[63] Li JL, Cornman RS, Evans JD, Pettis JS, Zhao Y, Murphy C, Peng WJ, Wu J, Hamilton M, Boncristiani HF, Jr, Zhou L, Hammond J, Chen YP. 2014. Systemic spread and propagation of a plant-pathogenic virus in European honeybees, Apis mellifera. mBio 5:e00898-13. 24449751 10.1128/mBio.00898-13 PMC3903276

[64] Selling BH, Allison RF, Kaesberg P. 1990. Genomic RNA of an insect virus directs synthesis of infectious virions in plants. Proc Natl Acad Sci USA 87:434–438. doi:10.1073/pnas.87.1.434. 2296598 PMC53278

[65] Parrish CR, Holmes EC, Morens DM, Park EC, Burke DS, Calisher CH, Laughlin CA, Saif LJ, Daszak P. 2008. Cross-species virus transmission and the emergence of new epidemic diseases. Microbiol Mol Biol Rev 72:457–470. doi:10.1128/MMBR.00004-08. 18772285 PMC2546865

[66] Chu Y, Zhao Z, Cai L, Zhang G. 2022. Viral diversity and biogeochemical potential revealed in different prawn-culture sediments by virus-enriched metagenome analysis. Environ Res 210:112901. doi:10.1016/j.envres.2022.112901. 35227678

[67] Cobbin JC, Charon J, Harvey E, Holmes EC, Mahar JE. 2021. Current challenges to virus discovery by meta-transcriptomics. Curr Opin Virol 51:48–55. doi:10.1016/j.coviro.2021.09.007. 34592710

[68] Chen S, Zhou Y, Chen Y, Gu J. 2018. fastp: an ultra-fast all-in-one FASTQ preprocessor. Bioinformatics 34:i884–i890. doi:10.1093/bioinformatics/bty560. 30423086 PMC6129281

[69] Haas BJ, Papanicolaou A, Yassour M, Grabherr M, Blood PD, Bowden J, Couger MB, Eccles D, Li B, Lieber M, MacManes MD, Ott M, Orvis J, Pochet N, Strozzi F, Weeks N, Westerman R, William T, Dewey CN, Henschel R, LeDuc RD, Friedman N, Regev A. 2013. De novo transcript sequence reconstruction from RNA-seq using the Trinity platform for reference generation and analysis. Nat Protoc 8:1494–1512. doi:10.1038/nprot.2013.084. 23845962 PMC3875132

[70] Li W, Godzik A. 2006. cd-hit: a fast program for clustering and comparing large sets of protein or nucleotide sequences. Bioinformatics 22:1658–1659. doi:10.1093/bioinformatics/btl158. 16731699

[71] Buchfink B, Reuter K, Drost HG. 2021. Sensitive protein alignments at tree-of-life scale using DIAMOND. Nat Methods 18:366–368. doi:10.1038/s41592-021-01101-x. 33828273 PMC8026399

[72] Chen XX, Wu WC, Shi M. 2021. Discovery and characterization of actively replicating DNA and retro-transcribing viruses in lower vertebrate hosts based on RNA sequencing. Viruses 13:1042. doi:10.3390/v13061042. 34072878 PMC8227577

[73] Langmead B, Salzberg SL. 2012. Fast gapped-read alignment with Bowtie 2. Nat Methods 9:357–359. doi:10.1038/nmeth.1923. 22388286 PMC3322381

[74] Thorvaldsdóttir H, Robinson JT, Mesirov JP. 2013. Integrative Genomics Viewer (IGV): high-performance genomics data visualization and exploration. Brief Bioinform 14:178–192. doi:10.1093/bib/bbs017. 22517427 PMC3603213

[75] Shi W, Shi M, Que TC, Cui XM, Ye RZ, Xia LY, Hou X, Zheng JJ, Jia N, Xie X, Wu WC, He MH, Wang HF, Wei YJ, Wu AQ, Zhang SF, Pan YS, Chen PY, Wang Q, Li SS, Zhong YL, Li YJ, Tan LH, Zhao L, Jiang JF, Hu YL, Cao WC. 2022. Trafficked Malayan pangolins contain viral pathogens of humans. Nat Microbiol 7:1259–1269. doi:10.1038/s41564-022-01181-1. 35918420 PMC9352580

[76] Shan T, Yang S, Wang H, Wang H, Zhang J, Gong G, Xiao Y, Yang J, Wang X, Lu J, Zhao M, Yang Z, Lu X, Dai Z, He Y, Chen X, Zhou R, Yao Y, Kong N, Zeng J, Ullah K, Wang X, Shen Q, Deng X, Zhang J, Delwart E, Tong G, Zhang W. 2022. Virome in the cloaca of wild and breeding birds revealed a diversity of significant viruses. Microbiome 10:60. doi:10.1186/s40168-022-01246-7. 35413940 PMC9001828

[77] Lu S, Wang J, Chitsaz F, Derbyshire MK, Geer RC, Gonzales NR, Gwadz M, Hurwitz DI, Marchler GH, Song JS, Thanki N, Yamashita RA, Yang M, Zhang D, Zheng C, Lanczycki CJ, Marchler-Bauer A. 2020. CDD/SPARCLE: the conserved domain database in 2020. Nucleic Acids Res 48:D265–D268. doi:10.1093/nar/gkz991. 31777944 PMC6943070

[78] Blum M, Chang HY, Chuguransky S, Grego T, Kandasaamy S, Mitchell A, Nuka G, Paysan-Lafosse T, Qureshi M, Raj S, Richardson L, Salazar GA, Williams L, Bork P, Bridge A, Gough J, Haft DH, Letunic I, Marchler-Bauer A, Mi H, Natale DA, Necci M, Orengo CA, Pandurangan AP, Rivoire C, Sigrist CJA, Sillitoe I, Thanki N, Thomas PD, Tosatto SCE, Wu CH, Bateman A, Finn RD. 2021. The InterPro protein families and domains database: 20 years on. Nucleic Acids Res 49:D344–D354. doi:10.1093/nar/gkaa977. 33156333 PMC7778928

[79] Kumar S, Stecher G, Li M, Knyaz C, Tamura K. 2018. MEGA X: Molecular Evolutionary Genetics Analysis across computing platforms. Mol Biol Evol 35:1547–1549. doi:10.1093/molbev/msy096. 29722887 PMC5967553

[80] Chen C, Chen H, Zhang Y, Thomas HR, Frank MH, He Y, Xia R. 2020. TBtools: an integrative toolkit developed for interactive analyses of big biological data. Mol Plant 13:1194–1202. doi:10.1016/j.molp.2020.06.009. 32585190

[81] Capella-Gutiérrez S, Silla-Martínez JM, Gabaldón T. 2009. trimAl: a tool for automated alignment trimming in large-scale phylogenetic analyses. Bioinformatics 25:1972–1973. doi:10.1093/bioinformatics/btp348. 19505945 PMC2712344

[82] Nguyen LT, Schmidt HA, von Haeseler A, Minh BQ. 2015. IQ-TREE: a fast and effective stochastic algorithm for estimating maximum-likelihood phylogenies. Mol Biol Evol 32:268–274. doi:10.1093/molbev/msu300. 25371430 PMC4271533

[83] Letunic I, Bork P. 2021. Interactive Tree Of Life (iTOL) v5: an online tool for phylogenetic tree display and annotation. Nucleic Acids Res 49:W293–W296. 10.1093/nar/gkab301. doi:10.1093/nar/gkab301. 33885785 PMC8265157

