# Supplementary material for: Virome Analysis of Normal and Growth Retardation Disease-Affected Macrobrachium rosenbergii
Source: Microbiol Spectr. 2022 Nov 29;10(6):e01462-22. doi: 10.1128/spectrum.01462-22 (PMC9769563; doi:10.1128/spectrum.01462-22)
Supplement: Supplemental file 1 — Fig. S1 to S5. Download spectrum.01462-22-s0001.pdf, PDF file, 3.3 MB [file spectrum.01462-22-s0001.pdf]

Supplementary Information for

Virome analysis reveals the viral spectrum of Growth Retardation Disease and normal *Macrobrachium rosenbergii* in China

Dandan Zhou,[a#] Shanshan Liu,[c#] Guangyu Guo,[a] Xinyi He,[a,b] Chengguang Xing,[c] Qijin Miao,[a] Gongrui Chen,[a,b] Xiaolin Chen,[a,b] Hongyu Yan,[a,b] Jiamin Zeng,[a,b] Zhenwen Zheng,[a,b] Hengwei Deng,[a,b,d] Shaoping Weng,[a,b*] Jianguo He[a,b*]

[a] State Key Laboratory of Biocontrol, Southern Marine Sciences and Engineering Guangdong Laboratory (Zhuhai), School of Marine Sciences, Sun Yat-sen University, Guangzhou, China.

[b] School of Life Sciences, Sun Yat-sen University, Guangzhou, China.

[c] School of Ecology, Sun Yat-sen University, Guangzhou, China.

[d] State Key Laboratory of Marine Resource Utilization in South China Sea, Hainan University, Haikou, China

[#]Dandan Zhou and Shanshan Liu contributed equally to this article.

[*]Corresponding author: Jianguo He, lsshjg@mail.sysu.edu.cn. and Shaoping Weng, lsswsp@mail.sysu.edu.cn

Supplementary Material

FIG S1 Venn diagram shows the unique and shared viral contigs in pool D and H.

FIG S2 Rarefaction curve of identified viral contigs and viral sequences reads in Pool D H.

FIG S3 The electrophoretograms of RT-PCR products for PCR confirmation and prevalence investigation amplified from GRD-affected *M. rosenbergii* samples.

FIG S4 The diagrams of RT-PCR products sequenced by the Sanger sequencing.

FIG S5 Mid-point rooted phylogenetic tree of members of new clade 1 in *Picornavirales*

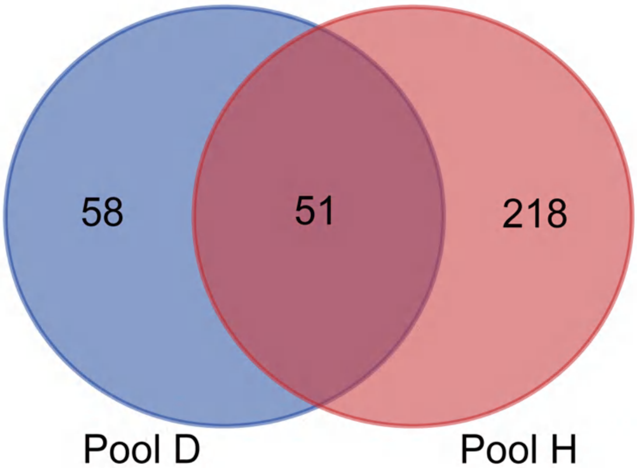

FIG S1 Venn diagram shows the unique and shared viral contigs in pool D and H.

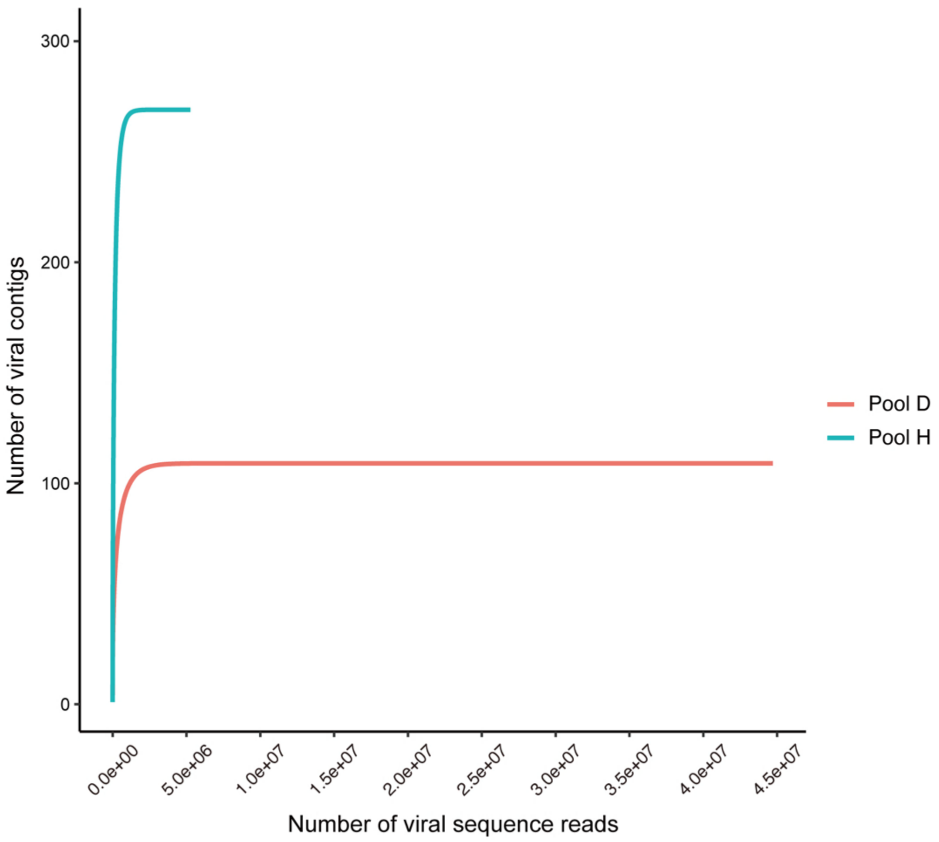

FIG S2 Rarefaction curve of identified viral contigs and viral sequences reads in Pool D H. The horizontal ordinate represents the number of viral sequence reads obtained from the clean data , the longitudinal axis represents the number of viral contigs observed from Pool D and Pool H.

A

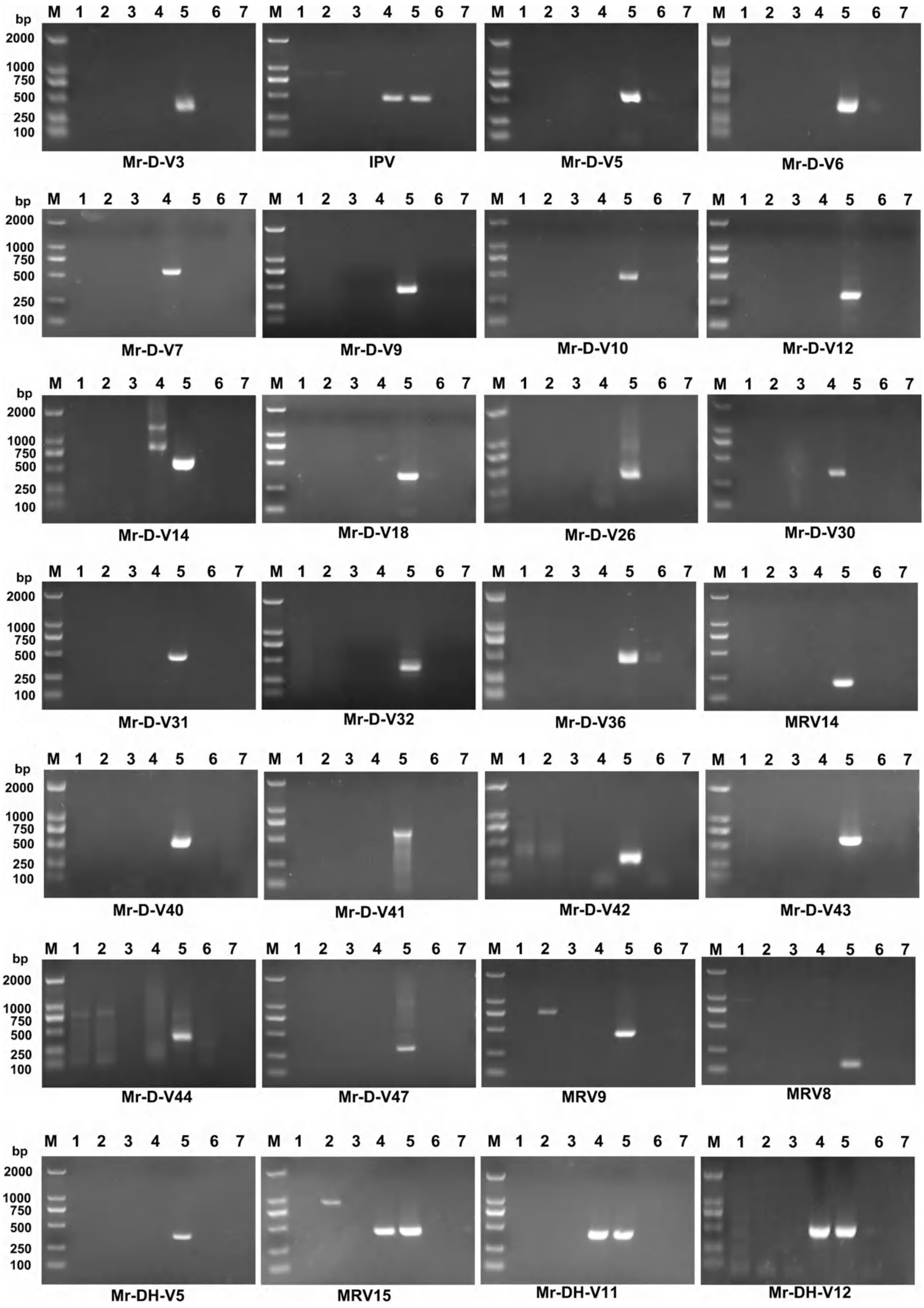
bp
2000
1000
750
500
250
100
M 1 2 3 4 5 6 7
Mr-D-V3
IPV
Mr-D-V5
Mr-D-V6
Mr-D-V7
Mr-D-V9
Mr-D-V10
Mr-D-V12
Mr-D-V14
Mr-D-V18
Mr-D-V26
Mr-D-V30
Mr-D-V31
Mr-D-V32
Mr-D-V36
MRV14
Mr-D-V40
Mr-D-V41
Mr-D-V42
Mr-D-V43
Mr-D-V44
Mr-D-V47
MRV9
MRV8
Mr-DH-V5
MRV15
Mr-DH-V11
Mr-DH-V12

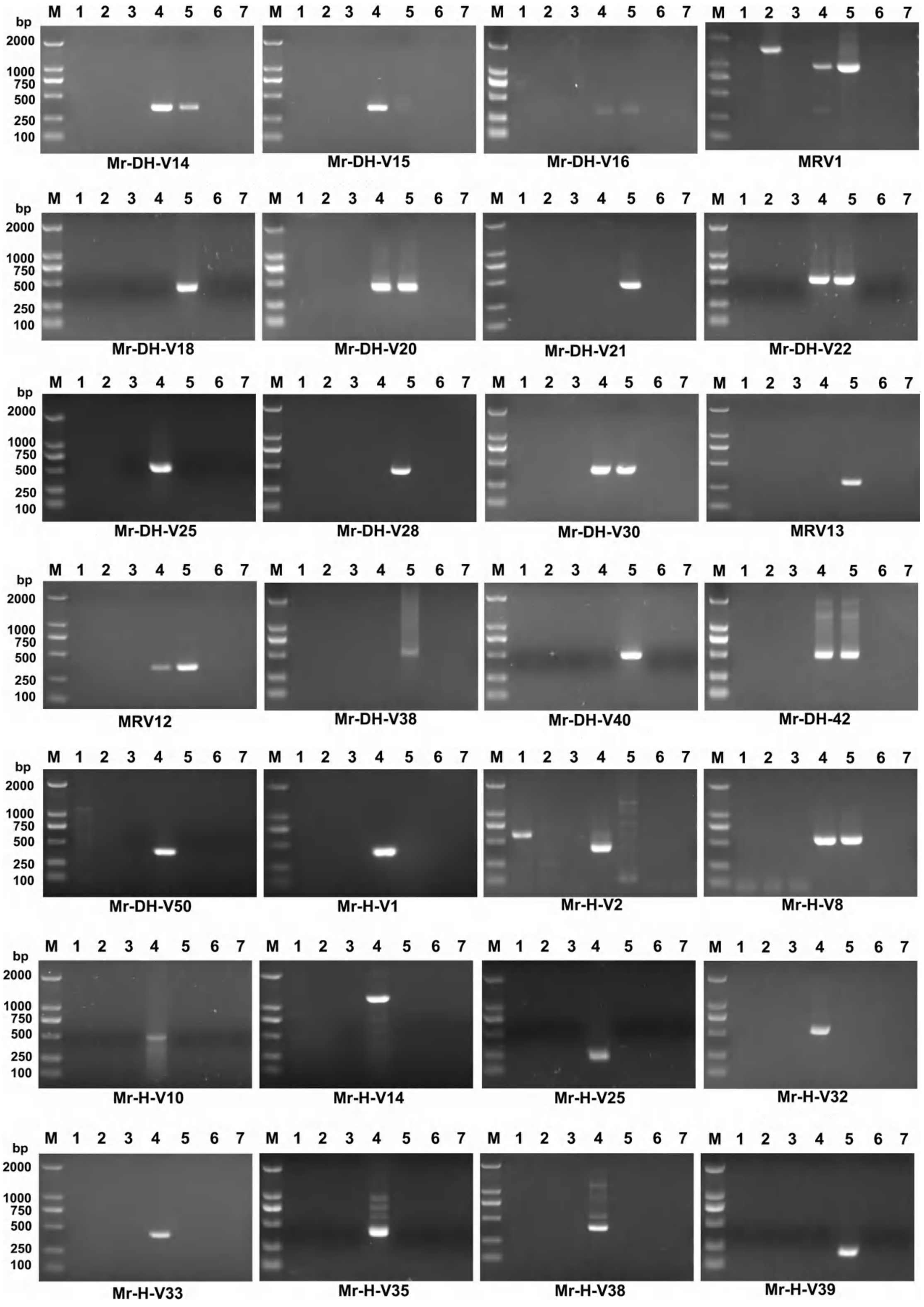
M 1 2 3 4 5 6 7
bp
2000
1000
750
500
250
100
Mr-DH-V14
Mr-DH-V15
Mr-DH-V16
MRV1
Mr-DH-V18
Mr-DH-V20
Mr-DH-V21
Mr-DH-V22
Mr-DH-V25
Mr-DH-V28
Mr-DH-V30
MRV13
MRV12
Mr-DH-V38
Mr-DH-V40
Mr-DH-42
Mr-DH-V50
Mr-H-V1
Mr-H-V2
Mr-H-V8
Mr-H-V10
Mr-H-V14
Mr-H-V25
Mr-H-V32
Mr-H-V33
Mr-H-V35
Mr-H-V38
Mr-H-V39

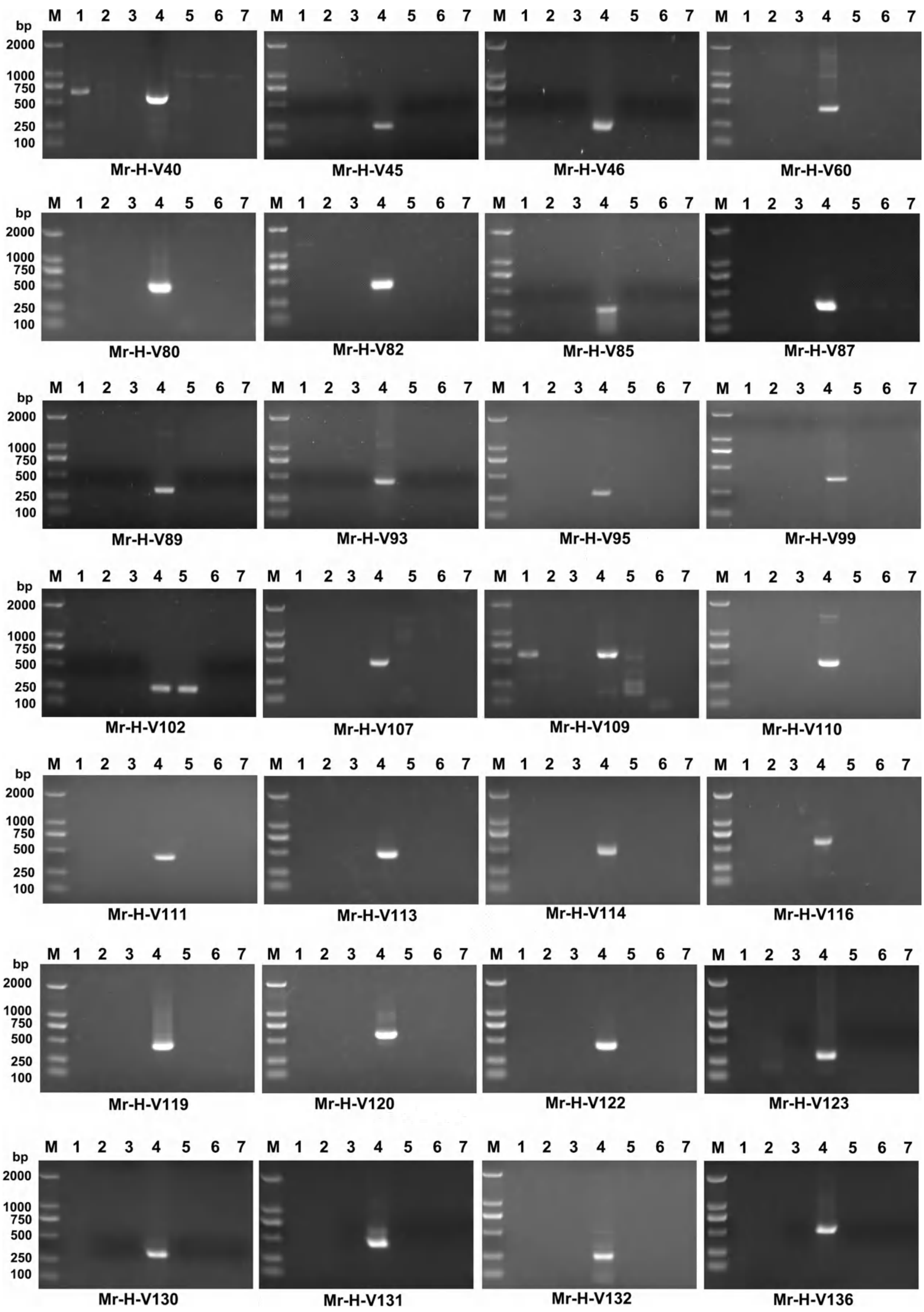
M 1 2 3 4 5 6 7
bp
2000
1000
750
500
250
100
Mr-H-V40
Mr-H-V45
Mr-H-V46
Mr-H-V60
Mr-H-V80
Mr-H-V82
Mr-H-V85
Mr-H-V87
Mr-H-V89
Mr-H-V93
Mr-H-V95
Mr-H-V99
Mr-H-V102
Mr-H-V107
Mr-H-V109
Mr-H-V110
Mr-H-V111
Mr-H-V113
Mr-H-V114
Mr-H-V116
Mr-H-V119
Mr-H-V120
Mr-H-V122
Mr-H-V123
Mr-H-V130
Mr-H-V131
Mr-H-V132
Mr-H-V136

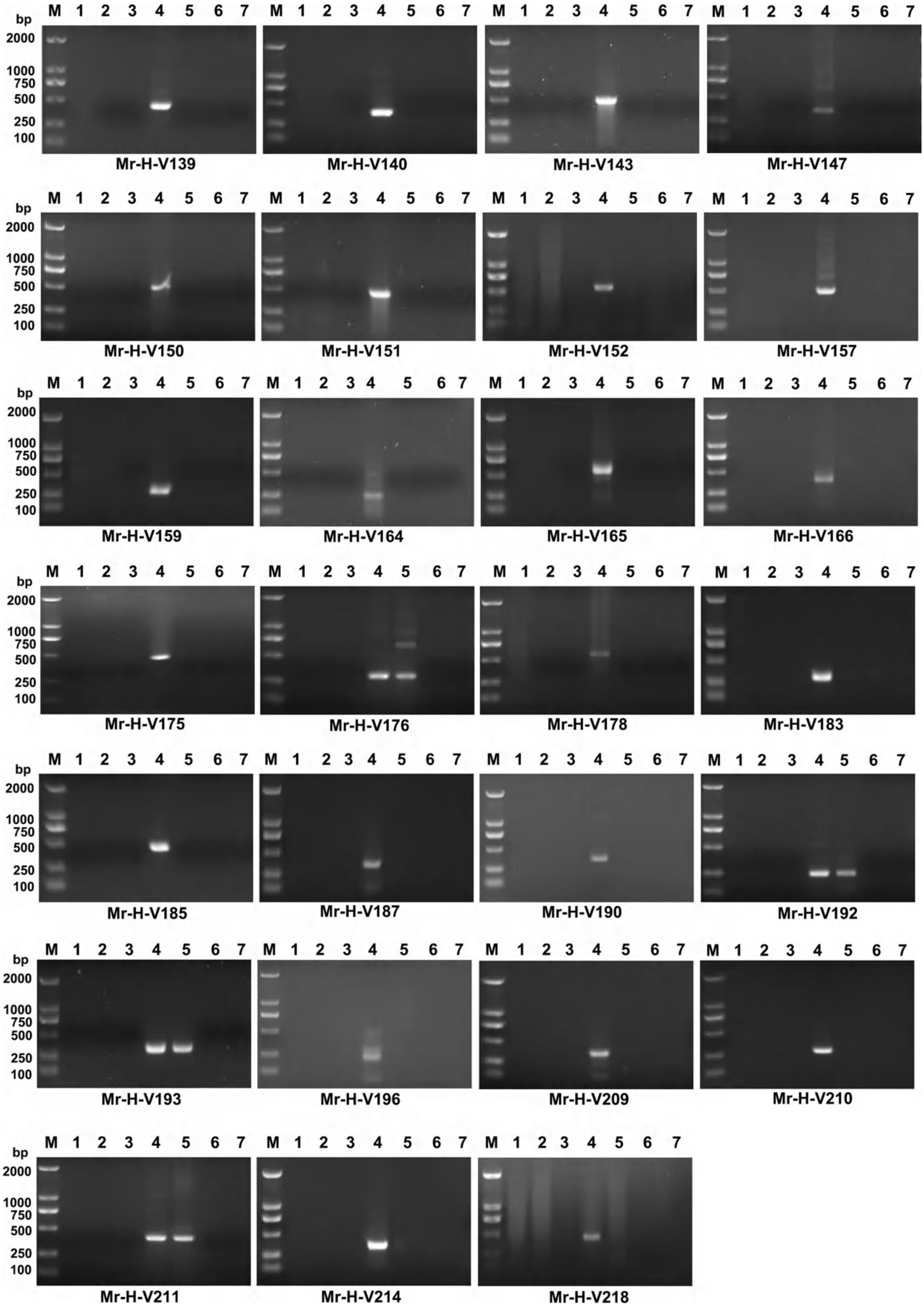
M 1 2 3 4 5 6 7
bp
2000
1000
750
500
250
100
Mr-H-V139
Mr-H-V140
Mr-H-V143
Mr-H-V147
Mr-H-V150
Mr-H-V151
Mr-H-V152
Mr-H-V157
Mr-H-V159
Mr-H-V164
Mr-H-V165
Mr-H-V166
Mr-H-V175
Mr-H-V176
Mr-H-V178
Mr-H-V183
Mr-H-V185
Mr-H-V187
Mr-H-V190
Mr-H-V192
Mr-H-V193
Mr-H-V196
Mr-H-V209
Mr-H-V210
Mr-H-V211
Mr-H-V214
Mr-H-V218

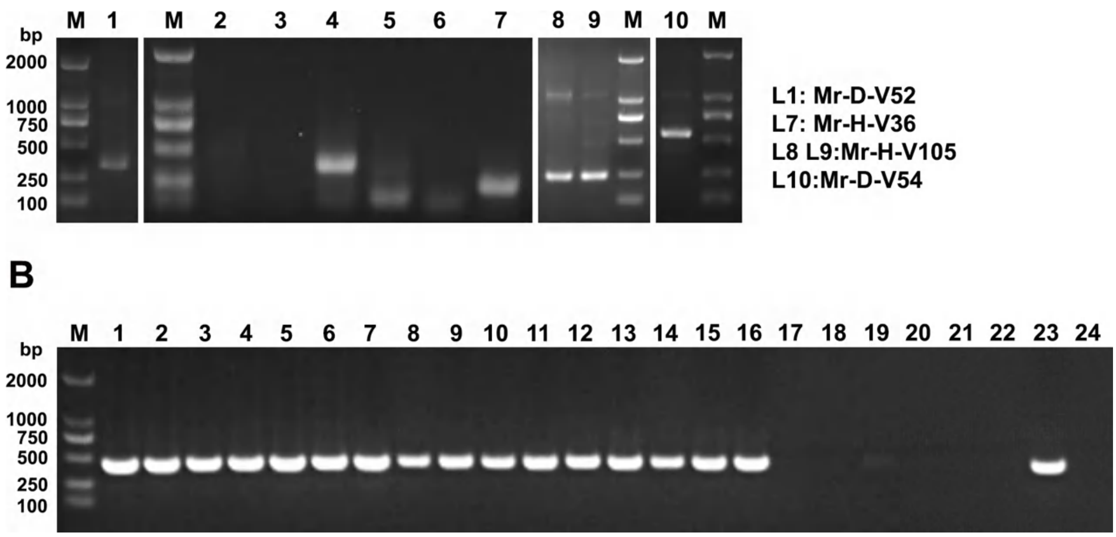

FIG S3 The electrophoretograms of RT-PCR products for PCR confirmation and prevalence investigation amplified from GRD-affected *M. rosenbergii* samples. (A) The electrophoretograms of 115 RT-PCR products of the 116 viral sequences in GRD-affected *M. rosenbergii* by nested RT-PCR. Mr-DH-V1 was not shown here, because of a lack of molecular maker. Each lane 1 and lane 2 were PCR products of sample H and D in the first PCR step, and Each lane 4 and line 5 were PCR products of sample H and D in the second PCR step. Each lane 3, 6, and 7 were negative control (RNase-free water) in two PCR steps. A molecular marker is in lane M. (B) The prevalence investigation of MRV15 in GRD-affected *M. rosenbergii* collected from Huzhou, in 2020. Twenty-three GRD-affected prawns were tested by a specific nested RT-PCR assay of MRV15. Lanes 1-23 were the PCR products of the second PCR step. Lane 24 was negative control (RNase-free water). A molecular marker is in lane M.

Mr-D-V3 (291 bp)

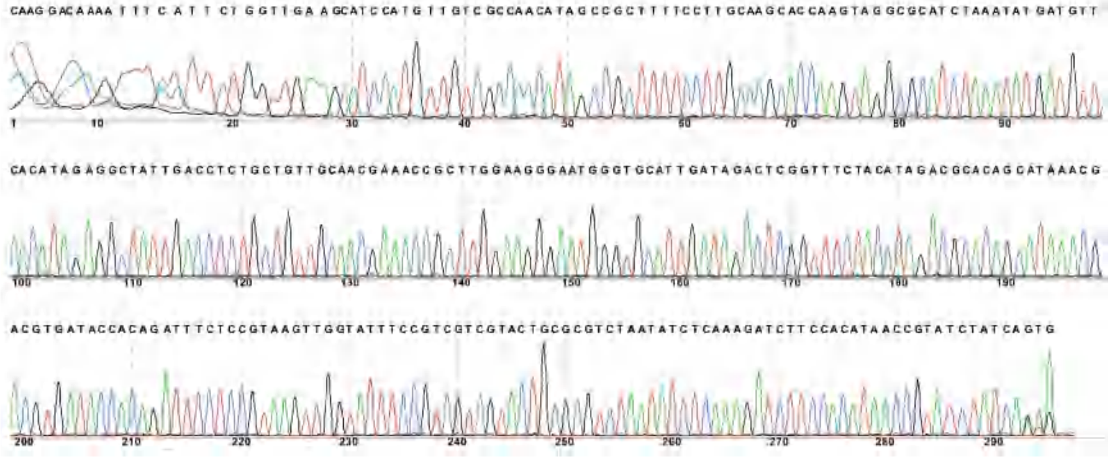

Infectious precocity virus strain ZJJS2019, IPV (862 bp)

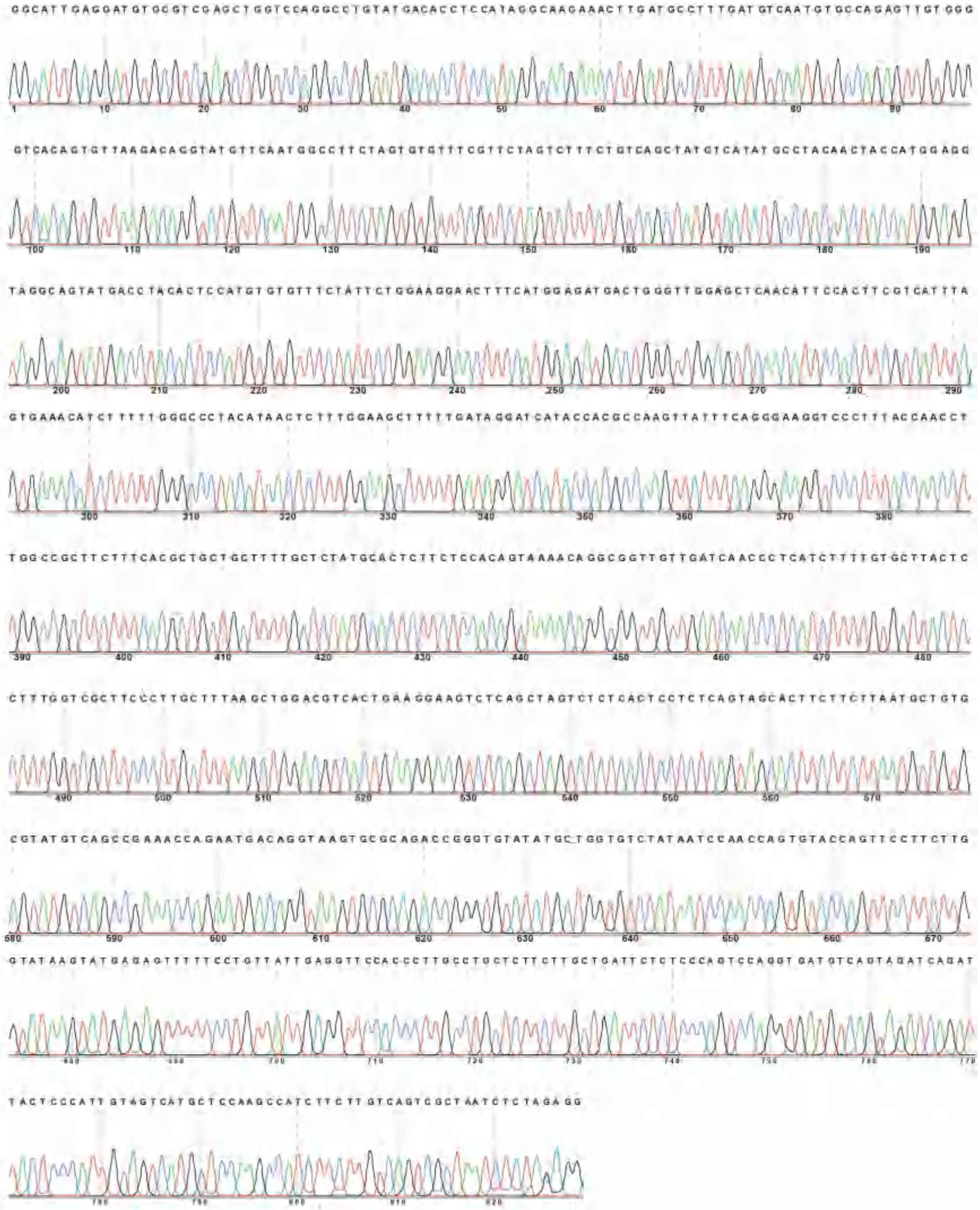

Mr-D-V5 (541 bp)

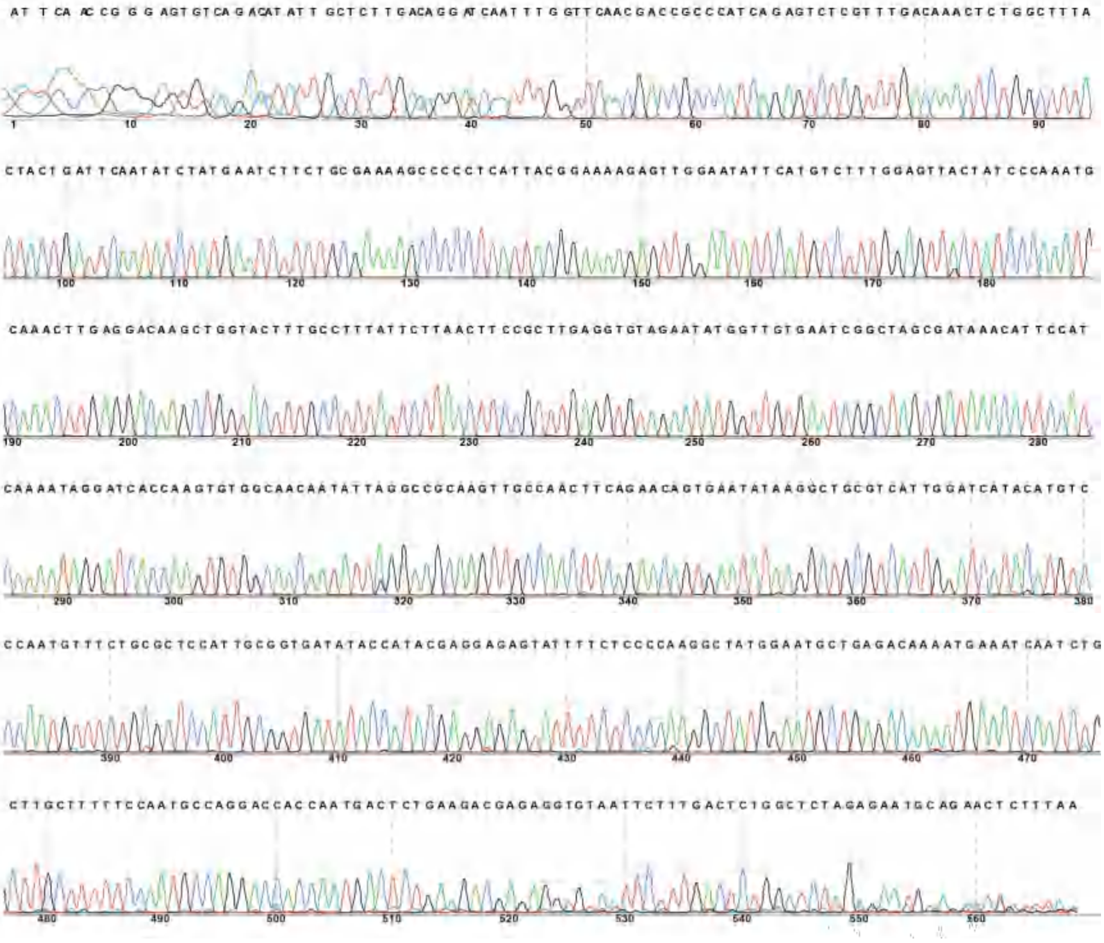

Mr-D-V6 (479 bp)

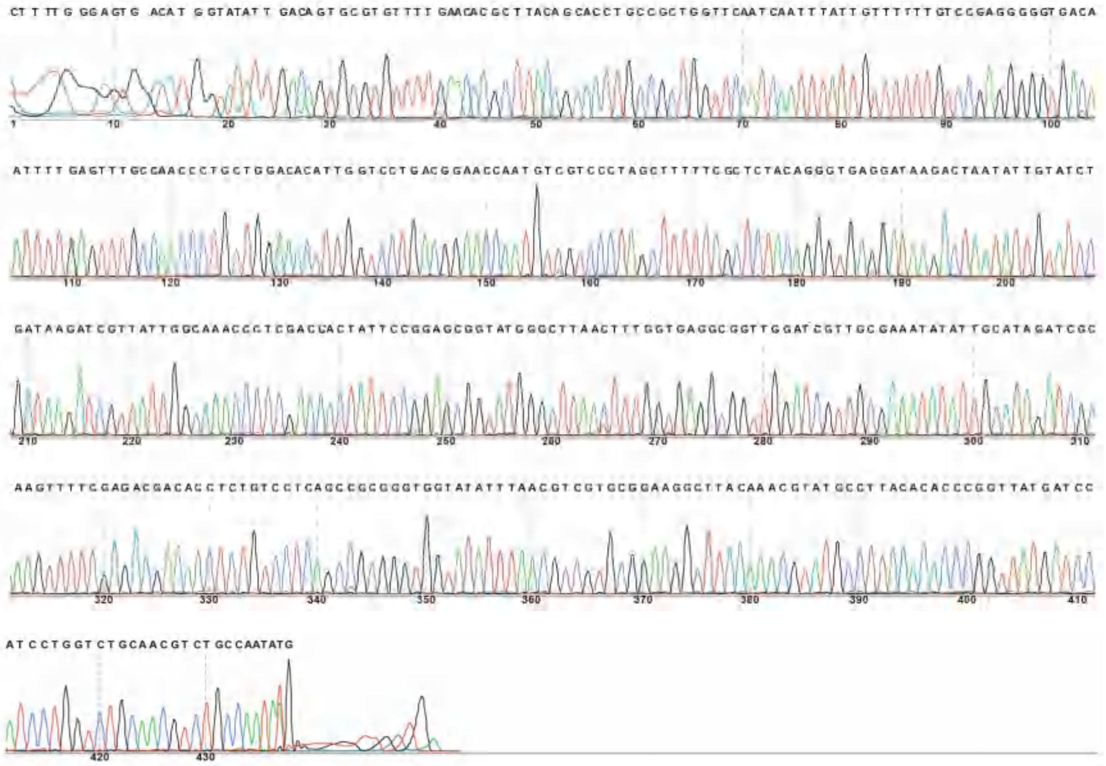

Mr-D-V7 (555 bp)

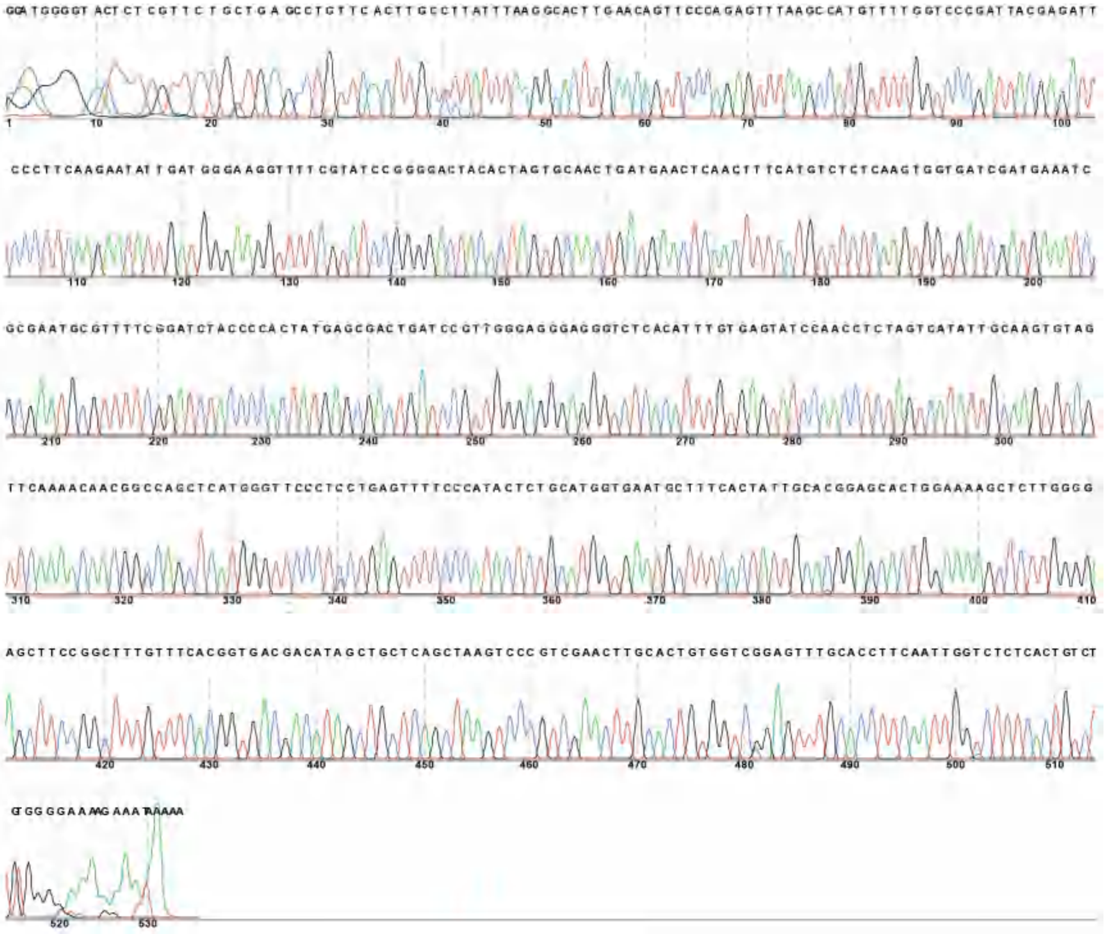

Mr-D-V9 (434 bp)

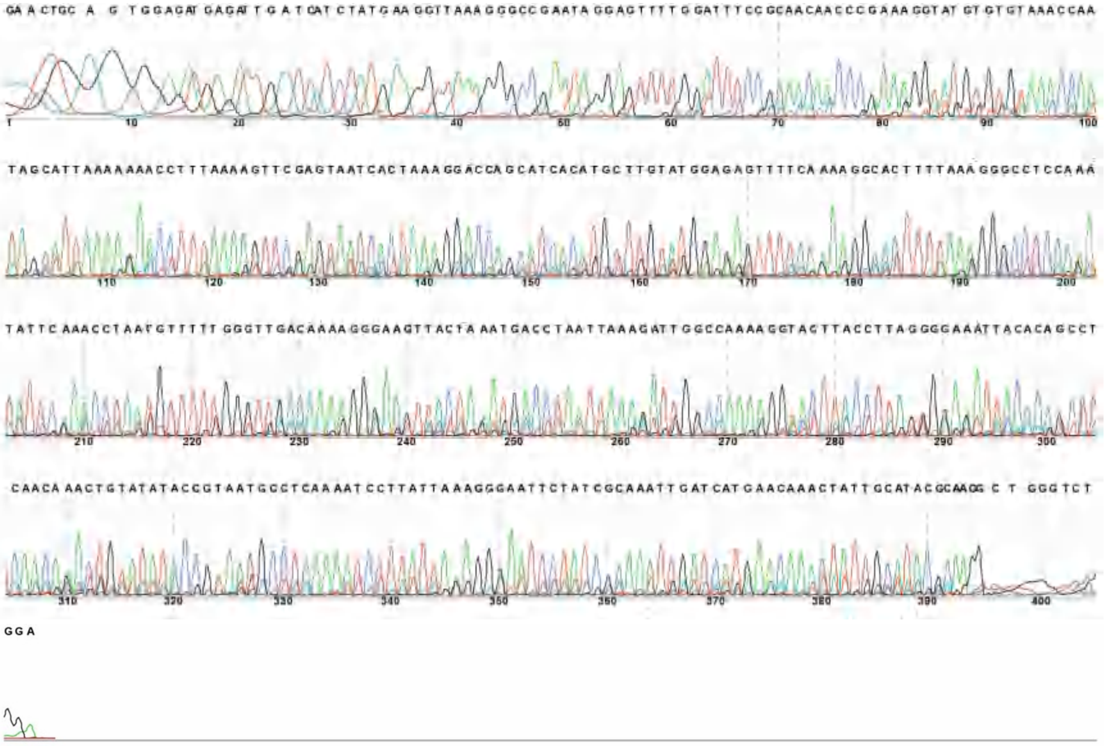

Mr-D-V10 (474 bp)

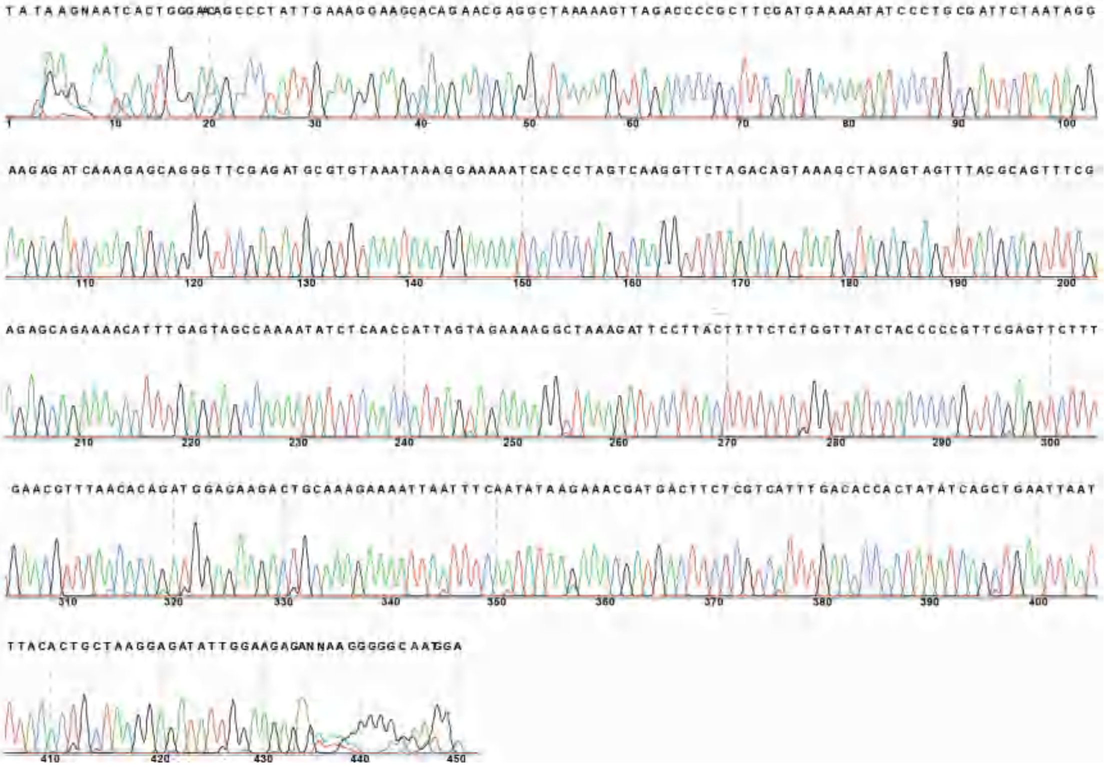

Mr-D-V12 (326 bp)

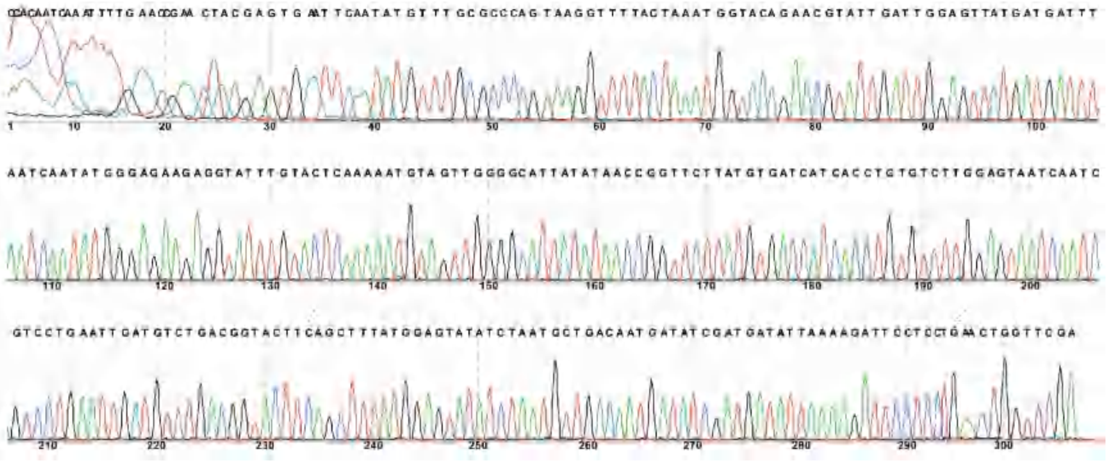

Mr-D-V14 (549bp)

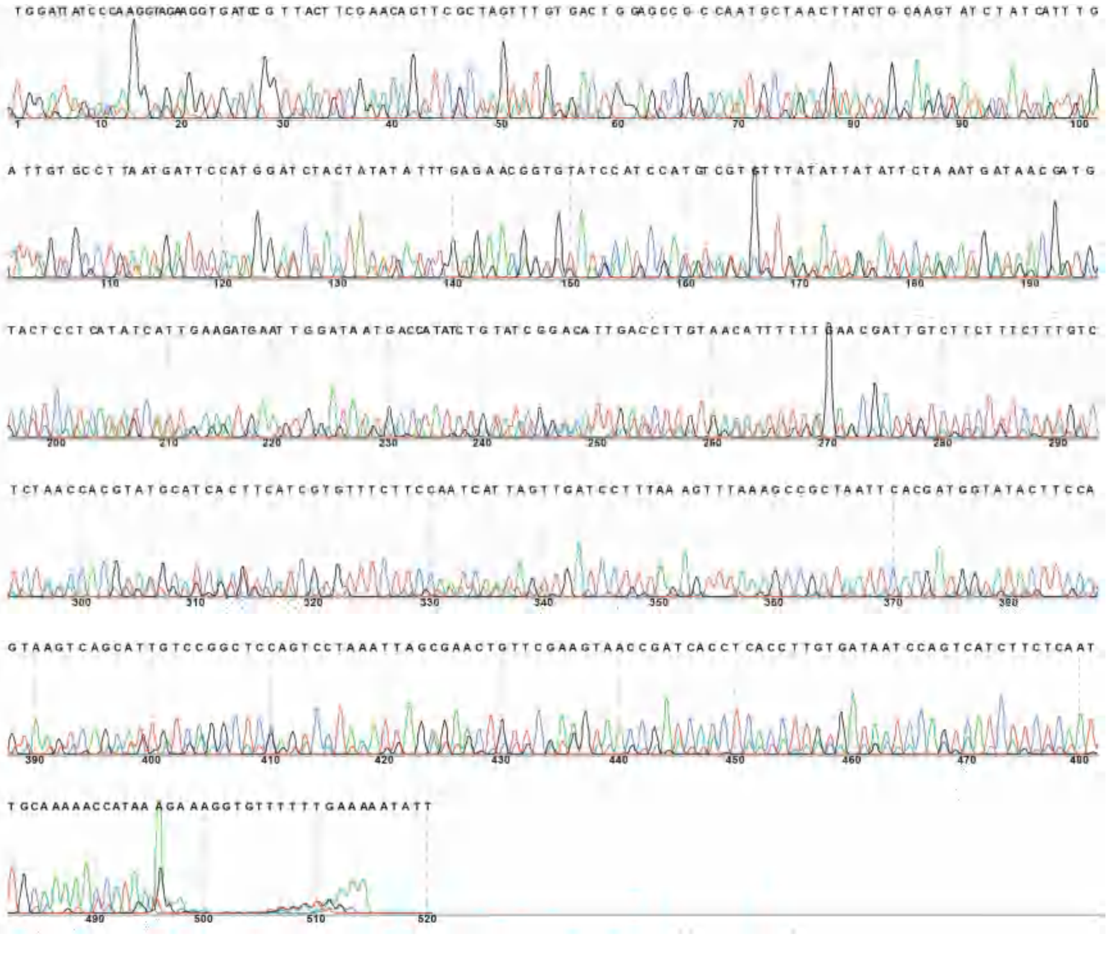

Mr-D-V18 (367 bp)

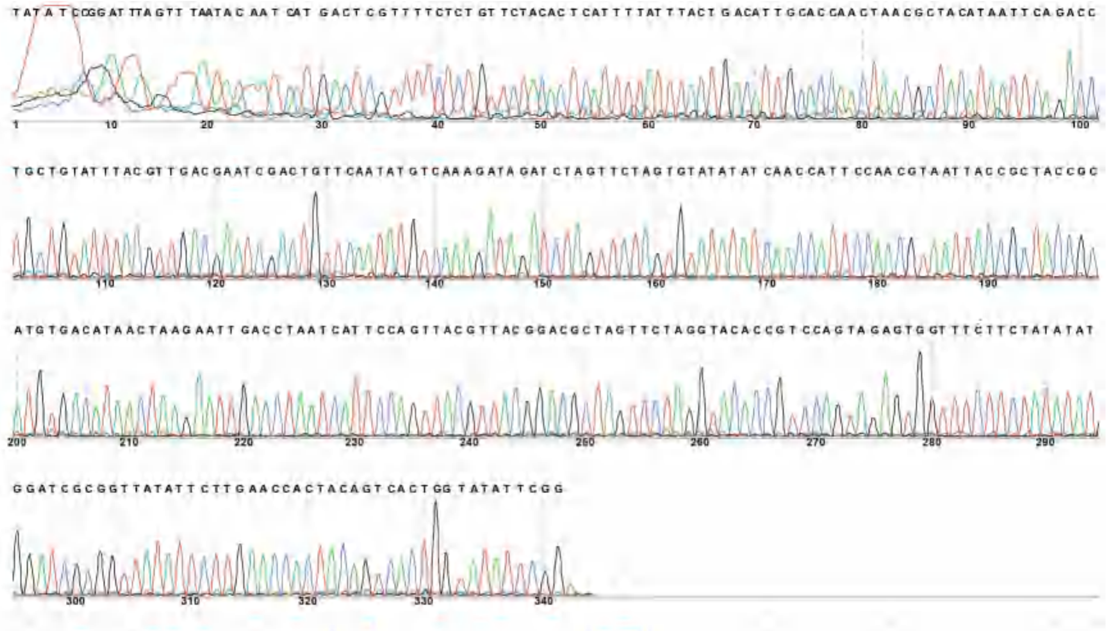

Mr-D-V26 (426 bp)

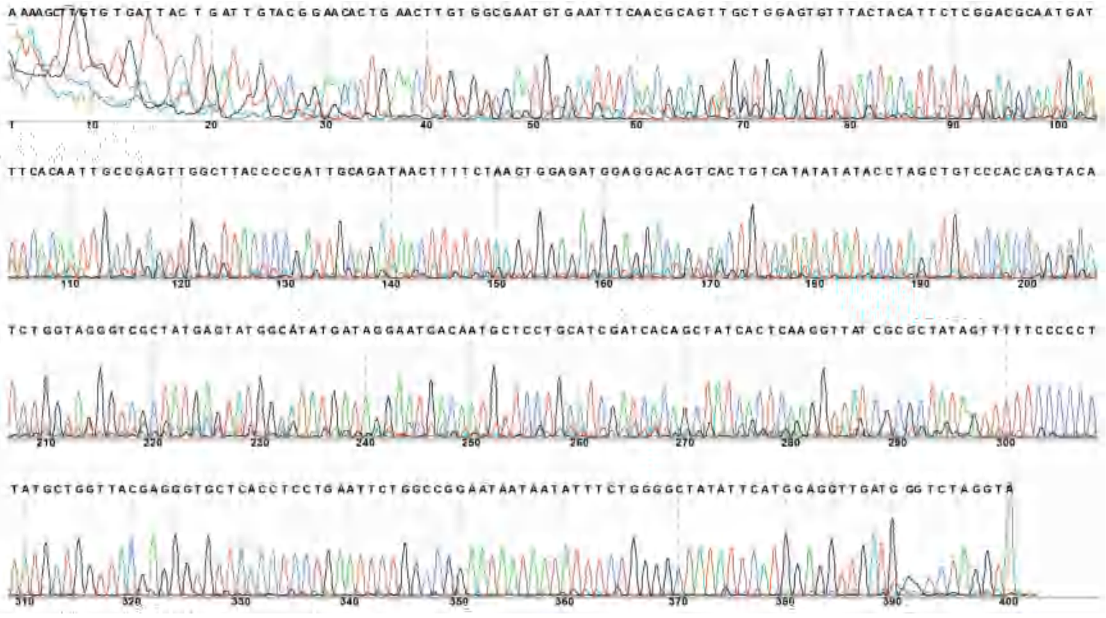

Mr-D-V30 (301 bp)

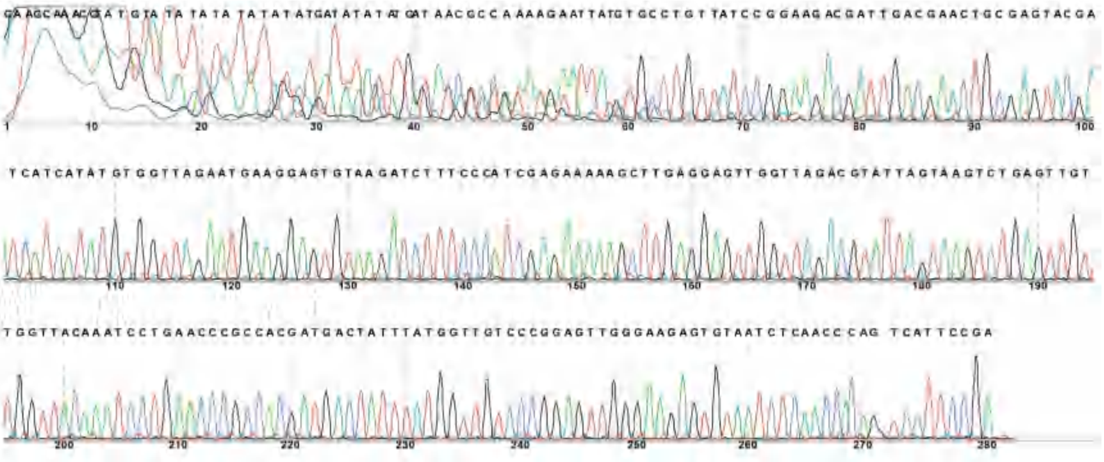

Mr-D-V31 (462 bp)

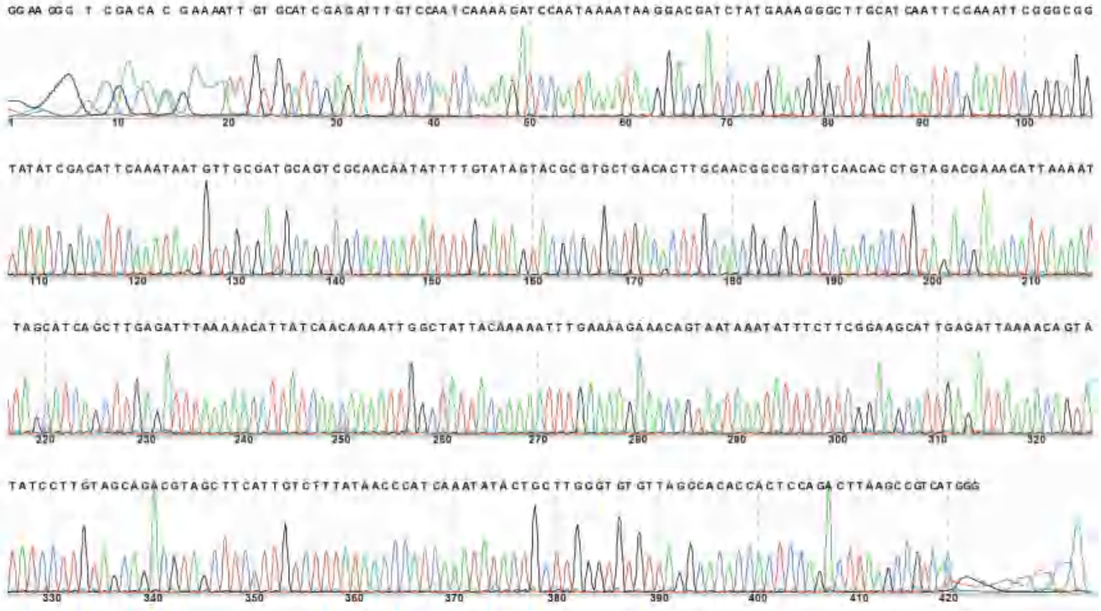

Mr-D-V32 (382 bp)

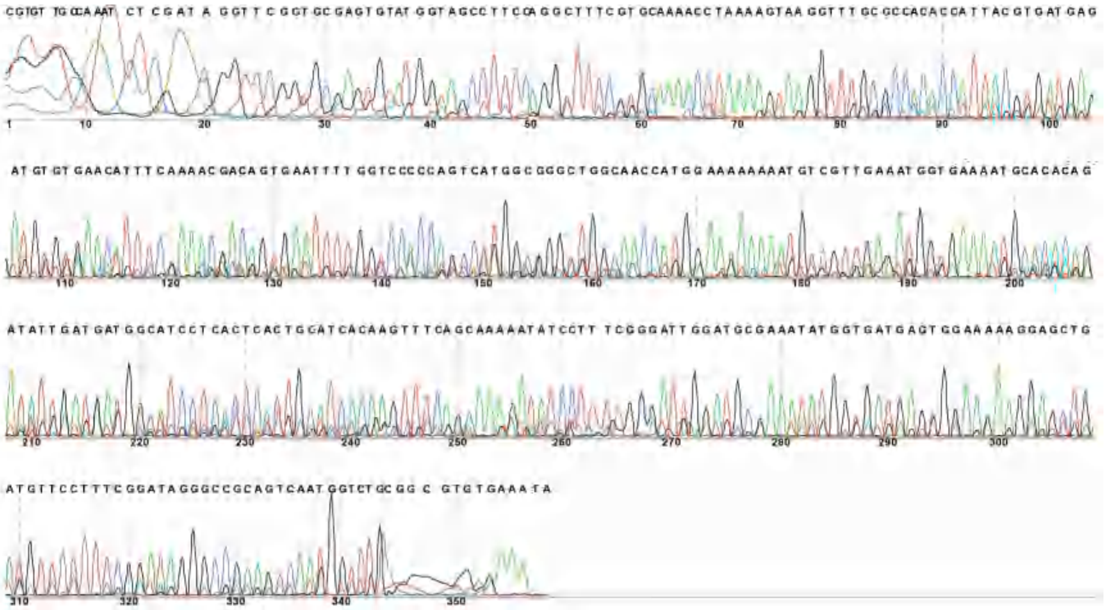

Mr-D-V36 (545 bp)

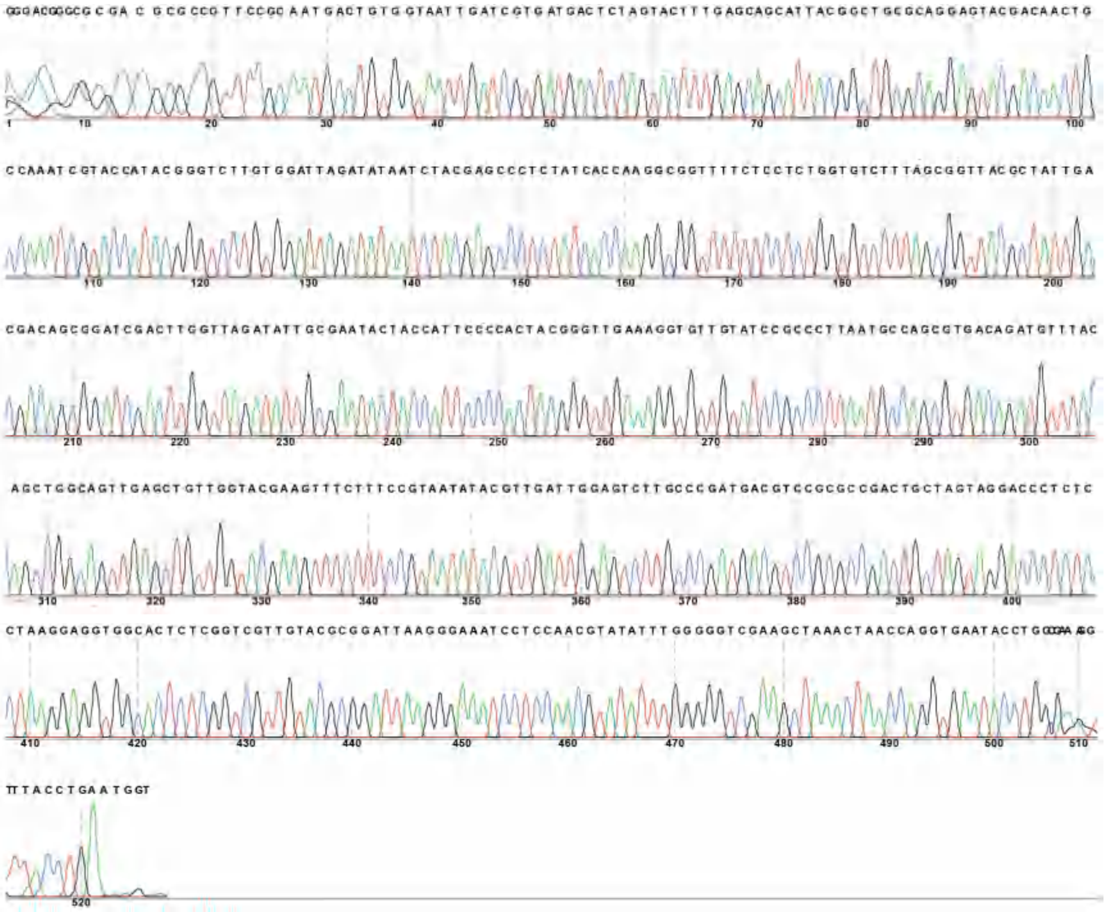

Macrobrachium rosenbergii virus 14, MRV14 (203 bp)

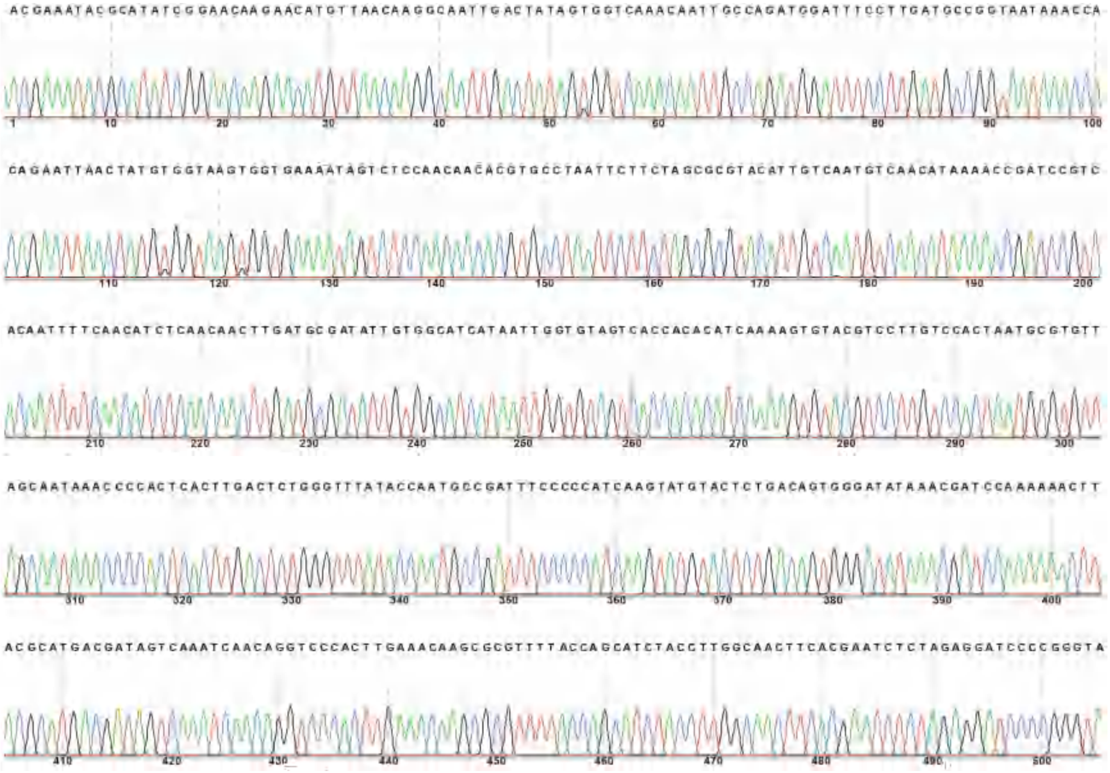

Mr-D-V40 (540 bp)

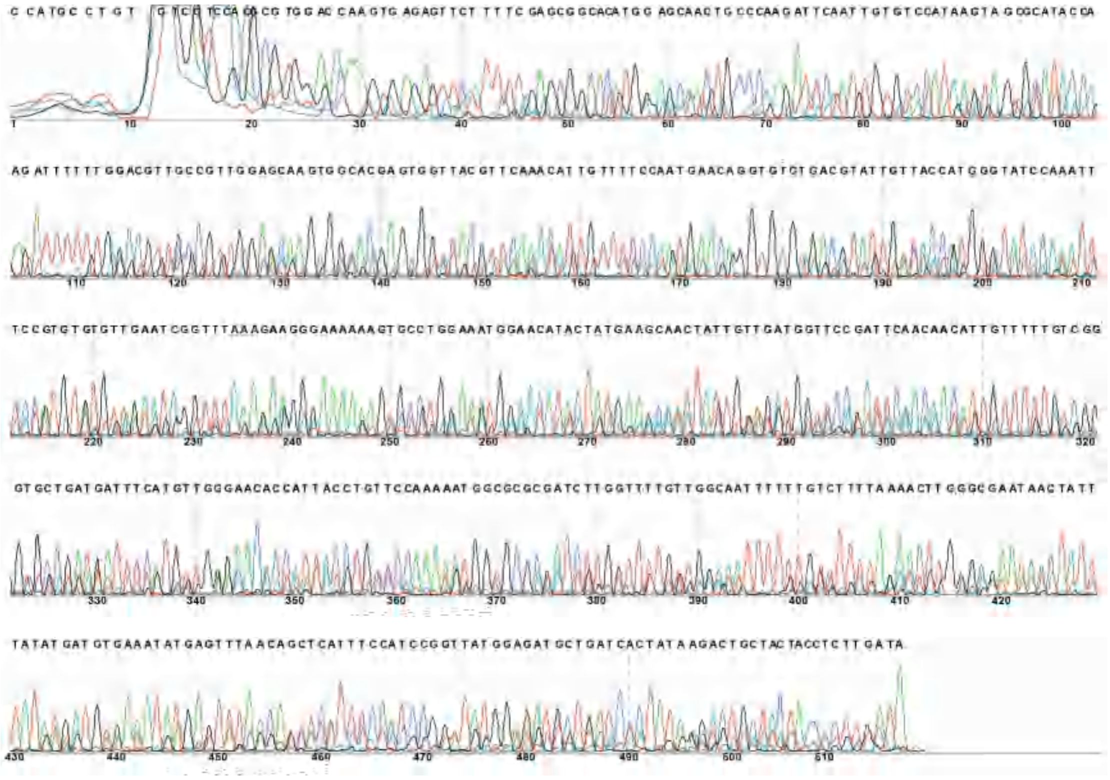

Mr-D-V41 (574 bp)

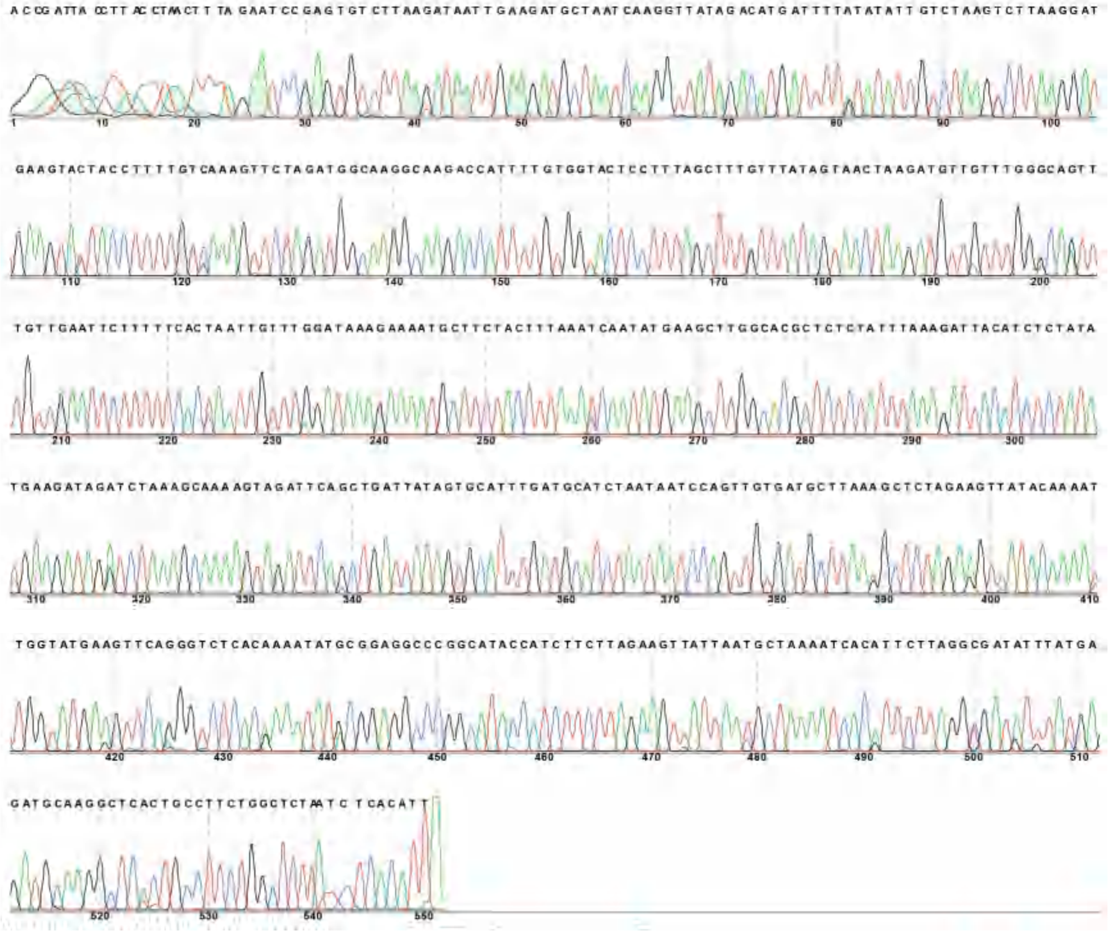

Mr-D-V42 (302 bp)

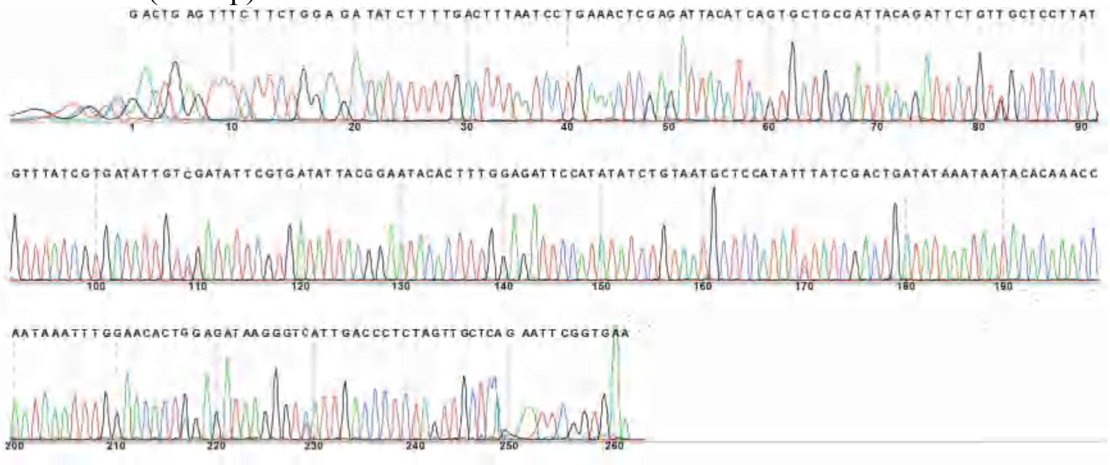

Mr-D-V43 (587 bp)

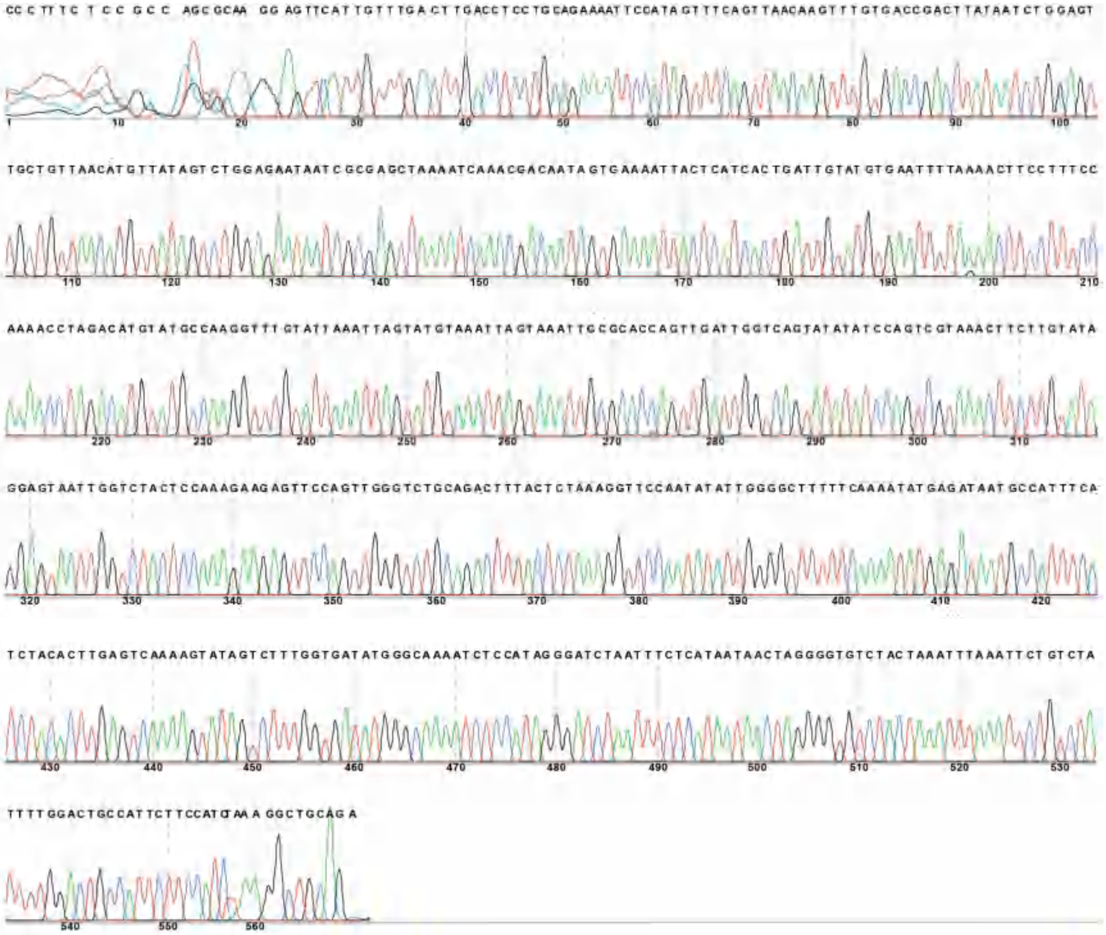

Mr-D-V44 (400 bp)

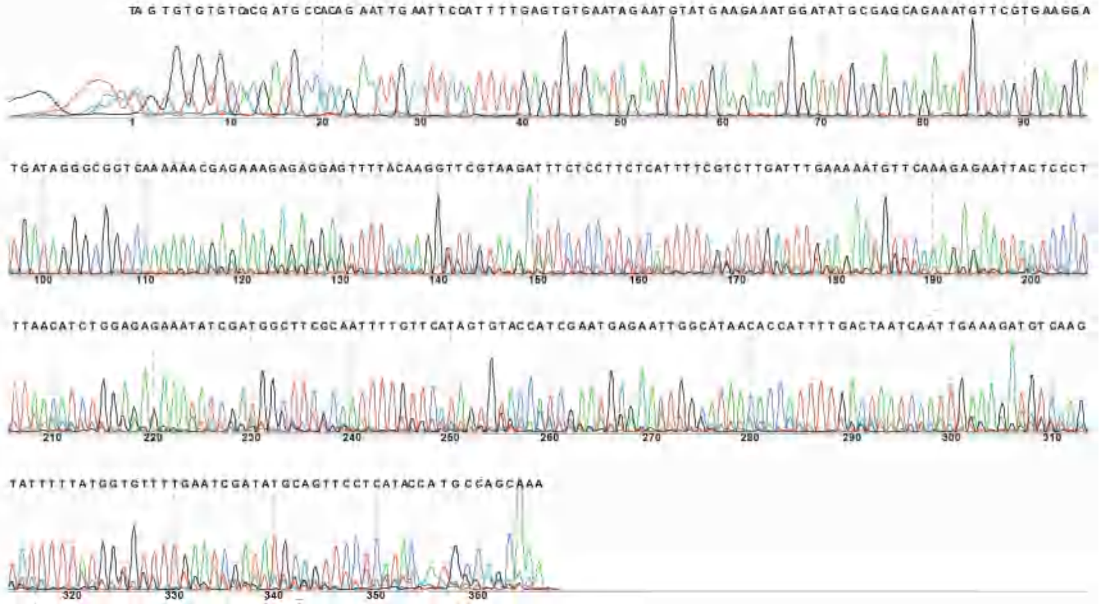

Mr-D-V47 (308 bp)

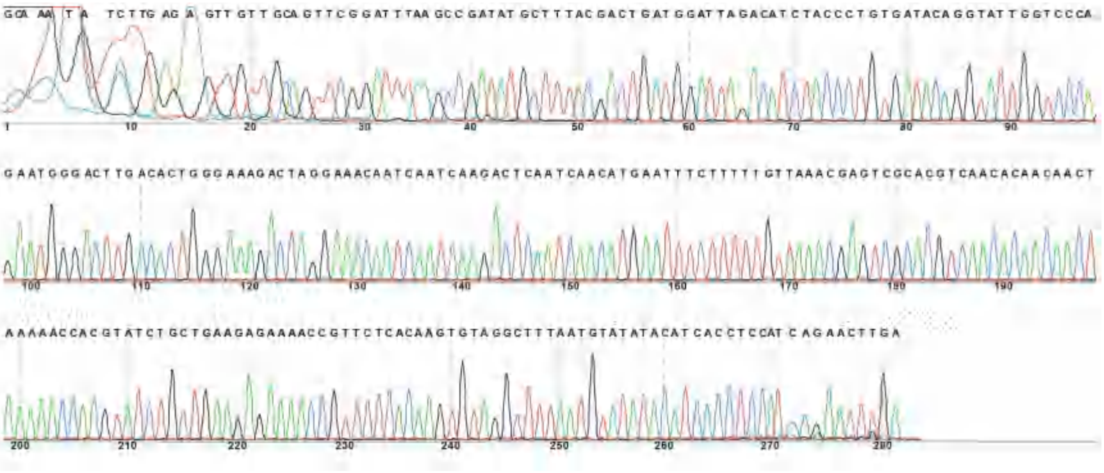

Mr-D-V52 (333 bp)

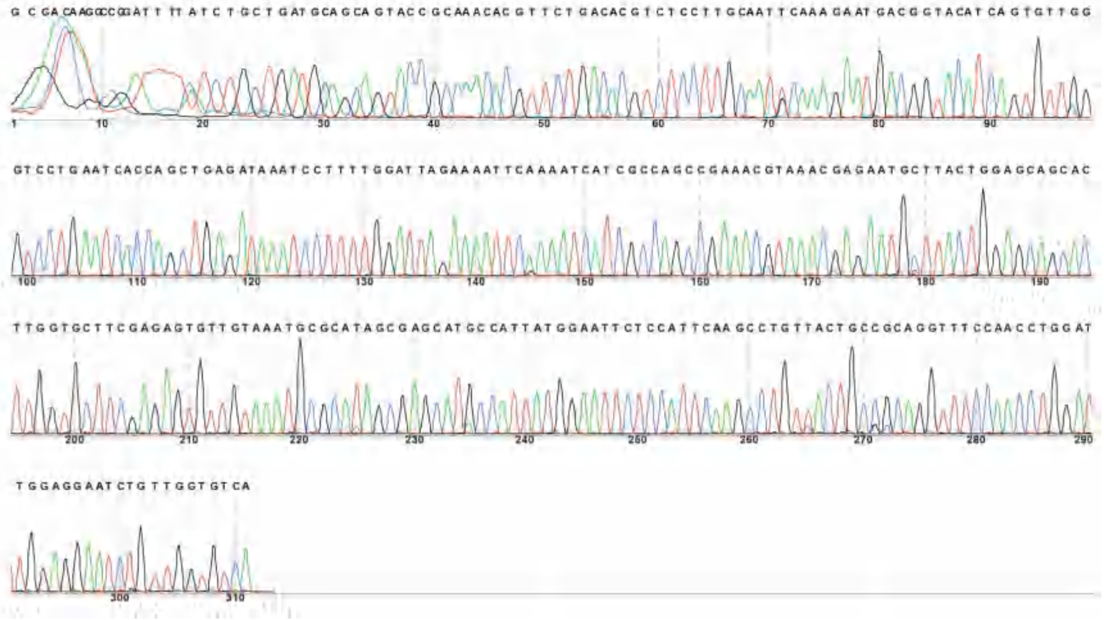

Mr-D-V54 (569 bp)

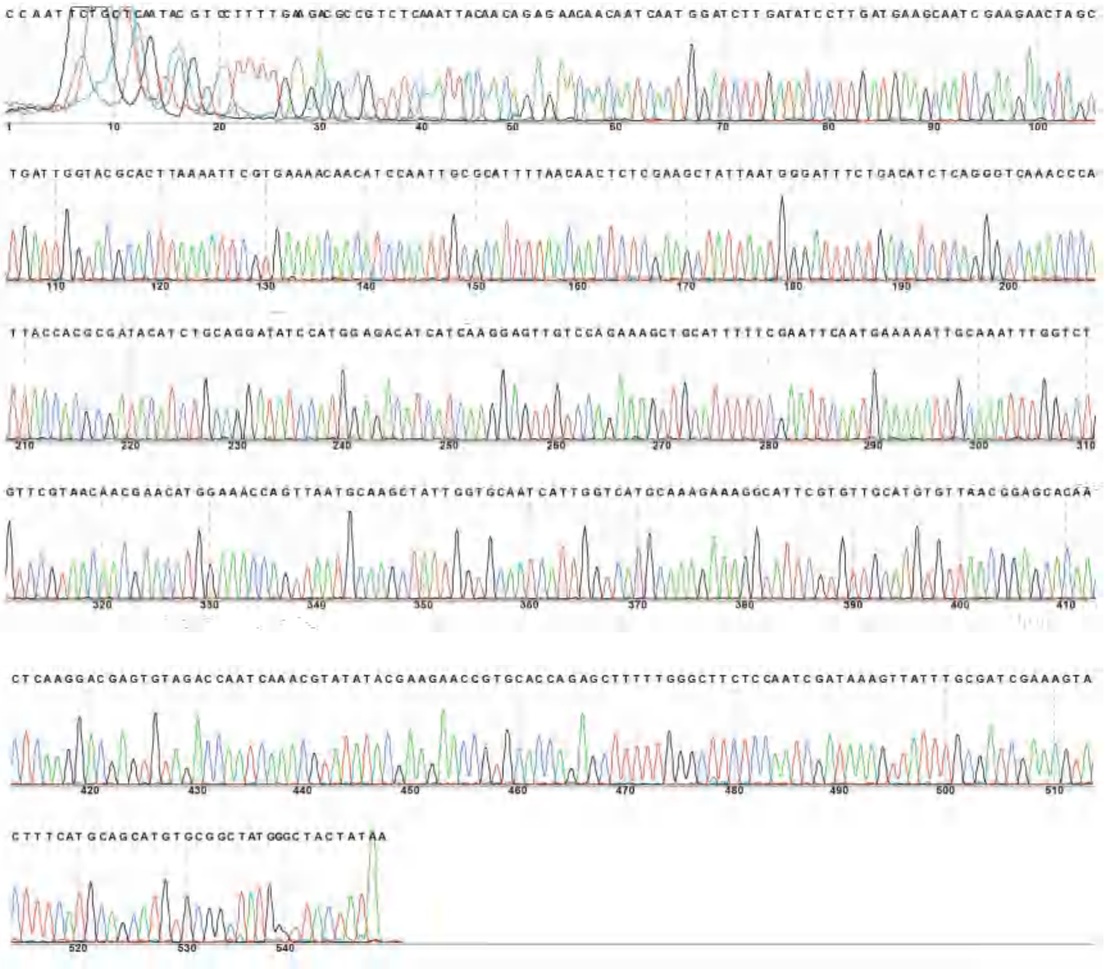

Mr-DH-V1 (421 bp)

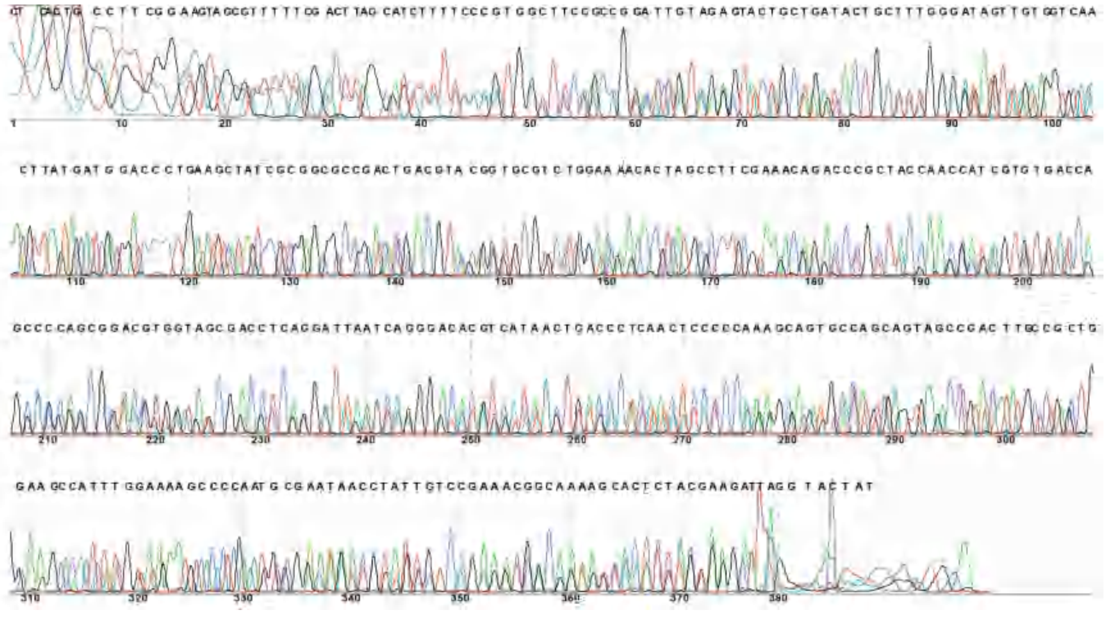

Macrobrachium rosenbergii virus 9, MRV9 (771 bp)

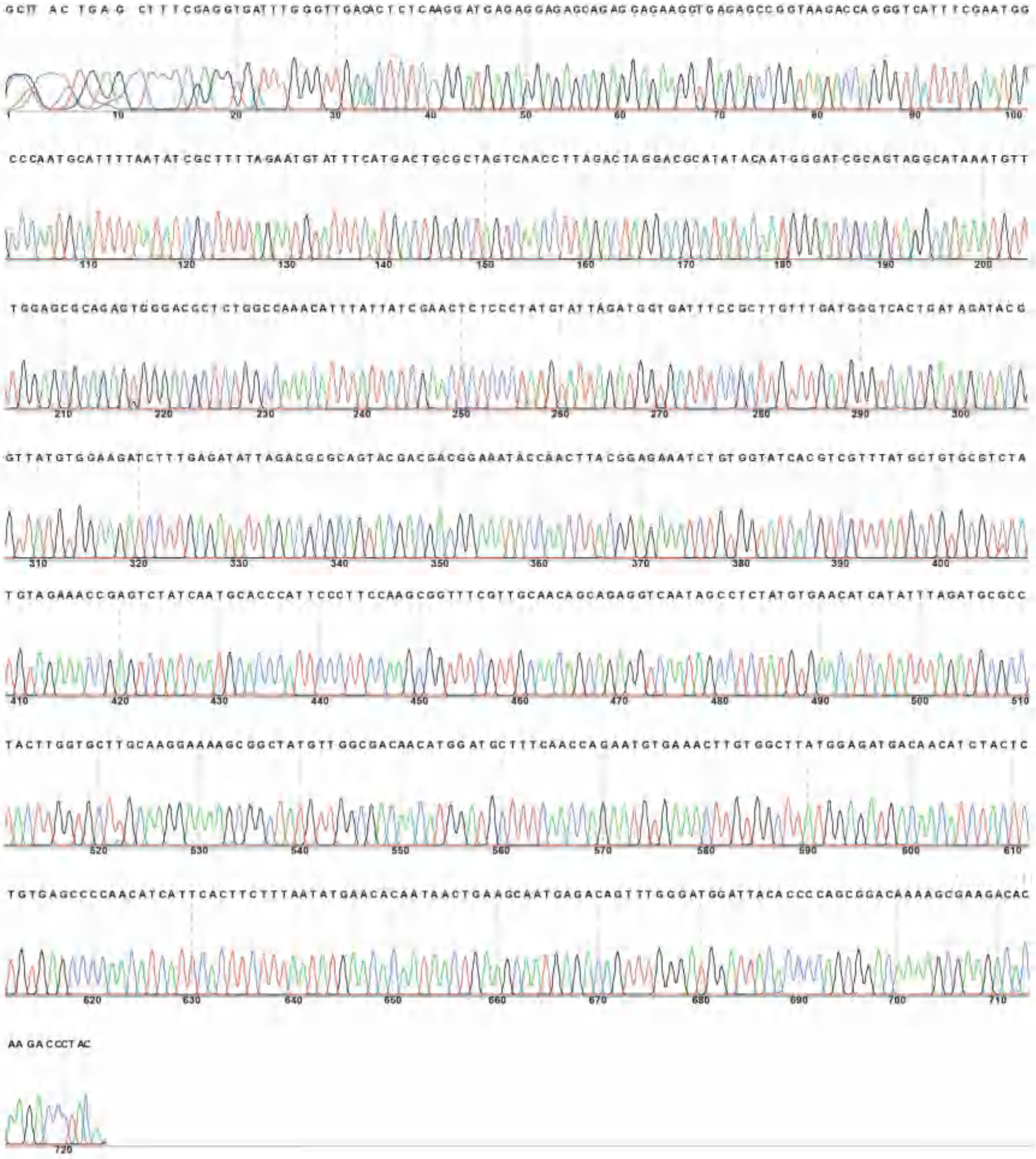

Mr-DH-V5 (382 bp)

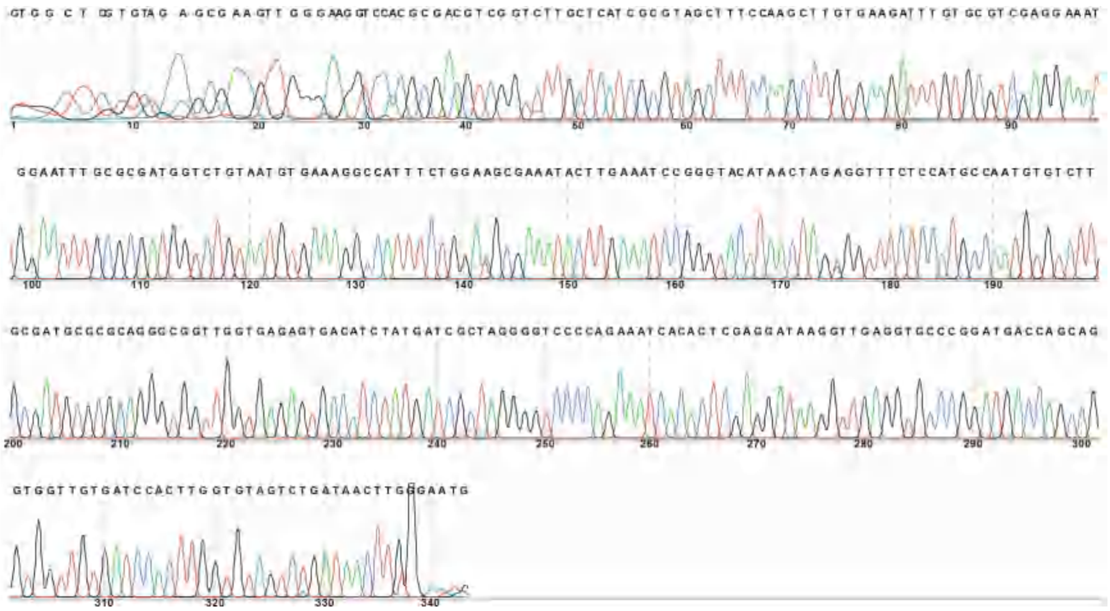

Macrobrachium rosenbergii virus 15, MRV15 (946 bp)

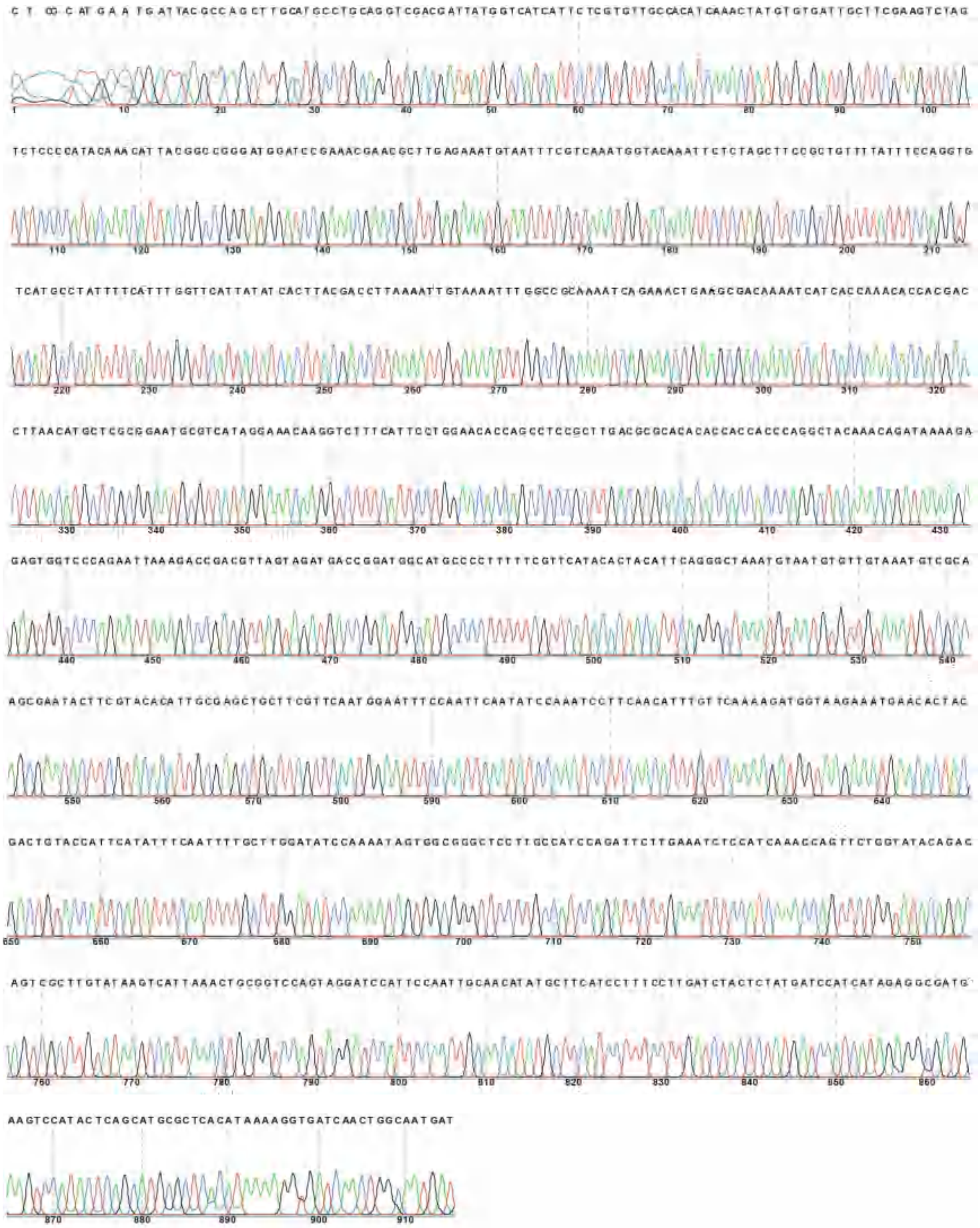

Mr-DH-V11 (195 bp)

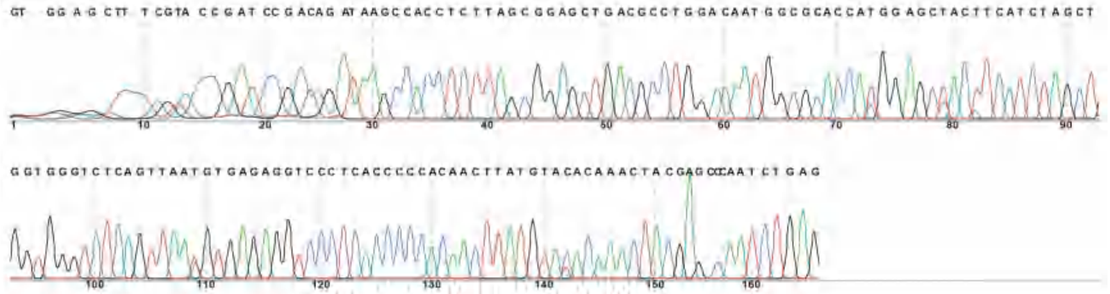

Mr-DH-V12 (481 bp)

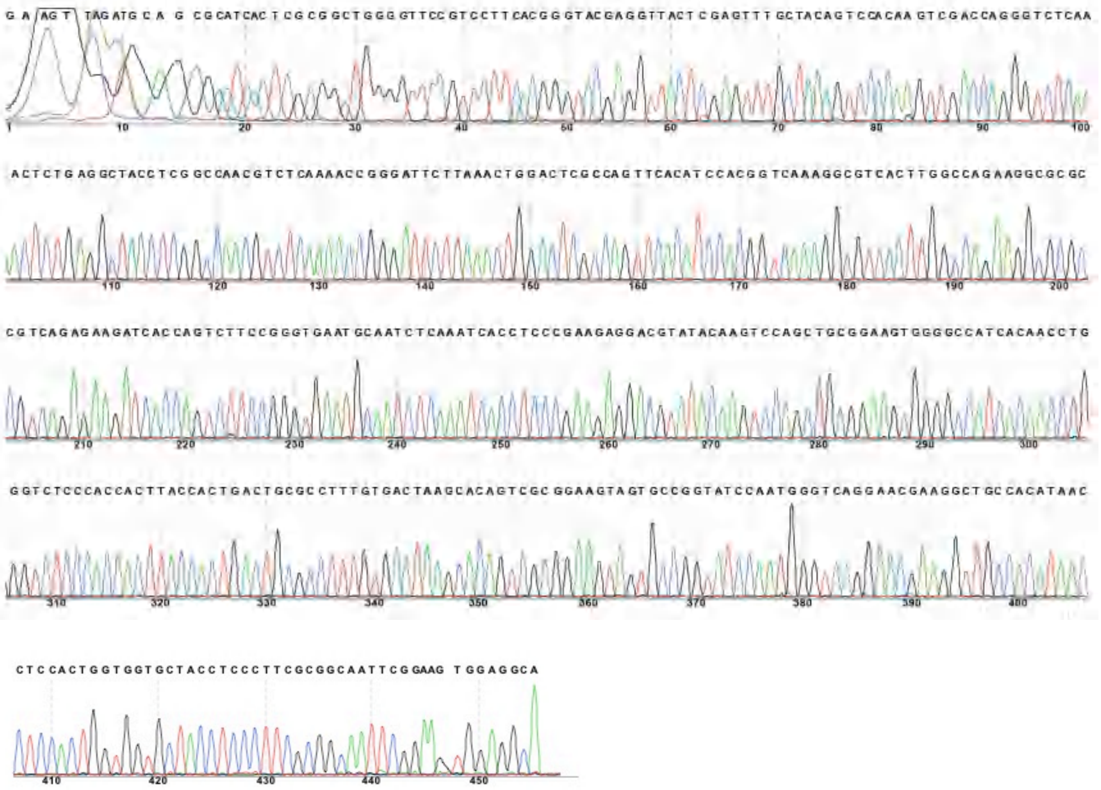

Mr-DH-V14 (369 bp)

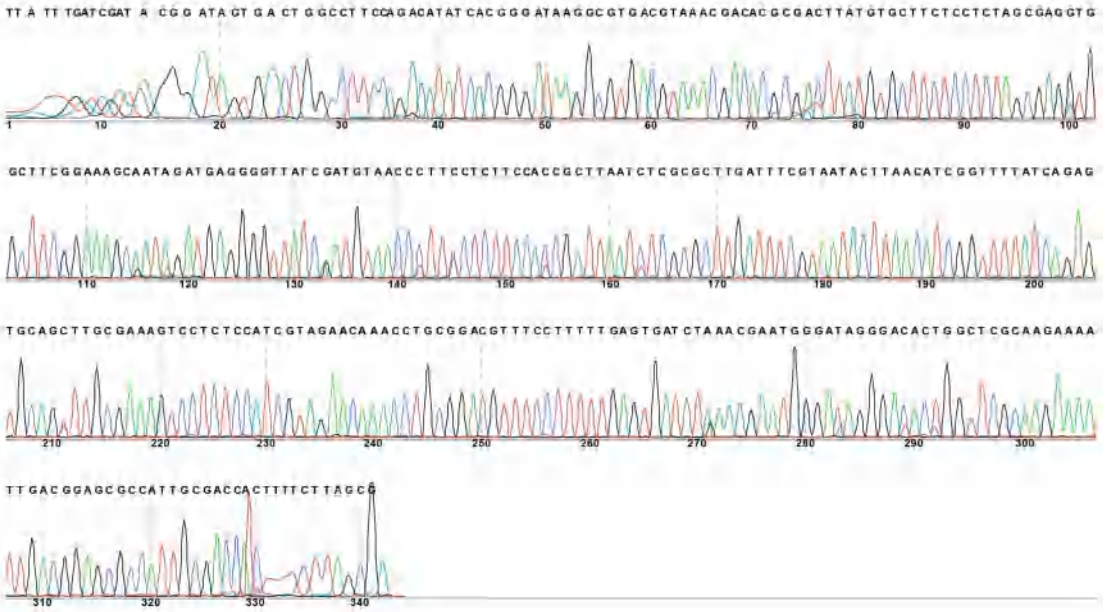

Mr-DH-V15 (369 bp)

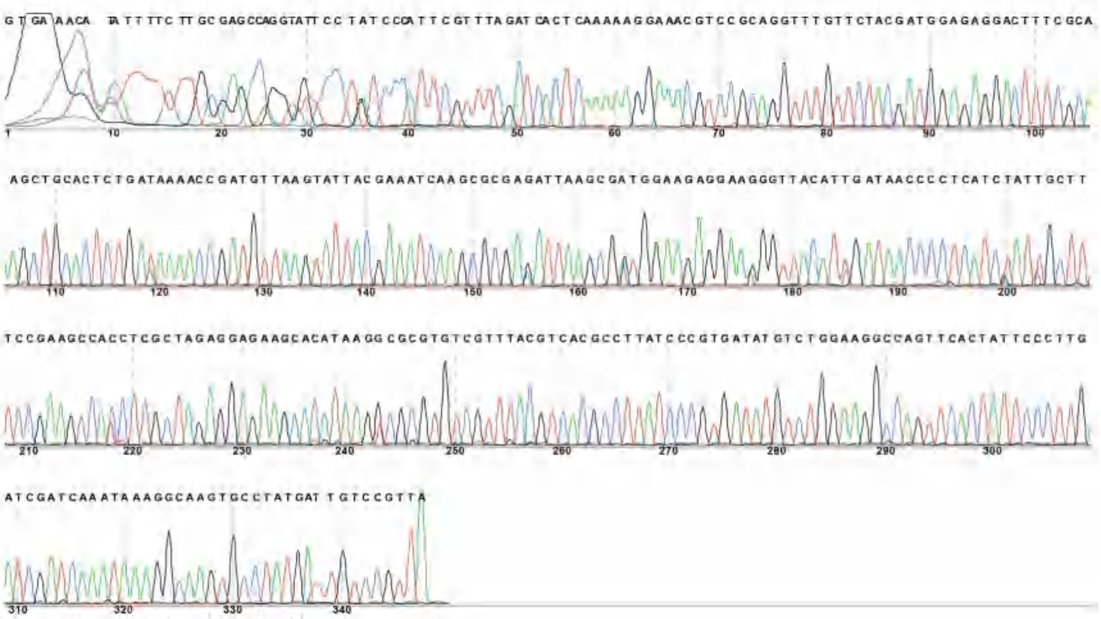

Mr-DH-V16 (320 bp)

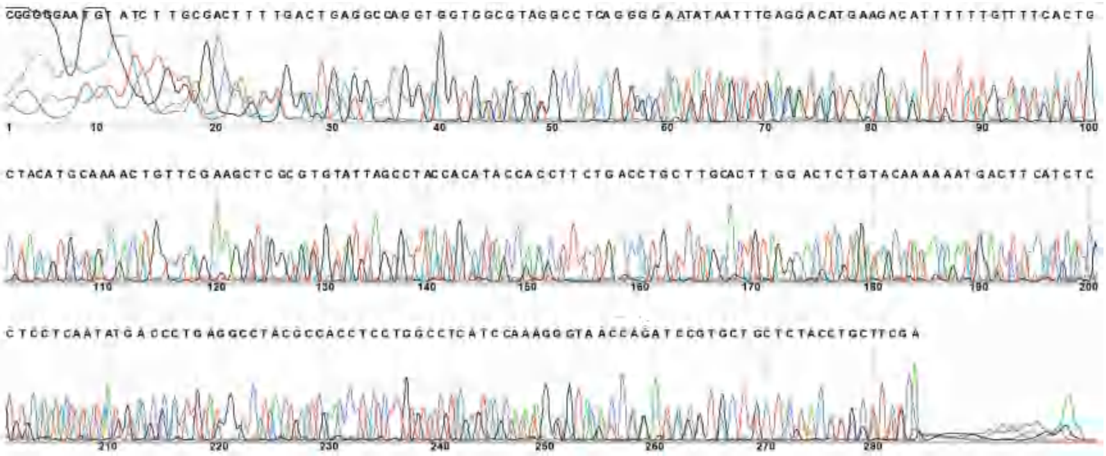

Macrobrachium rosenbergii virus 1, MRV1 (928 bp)

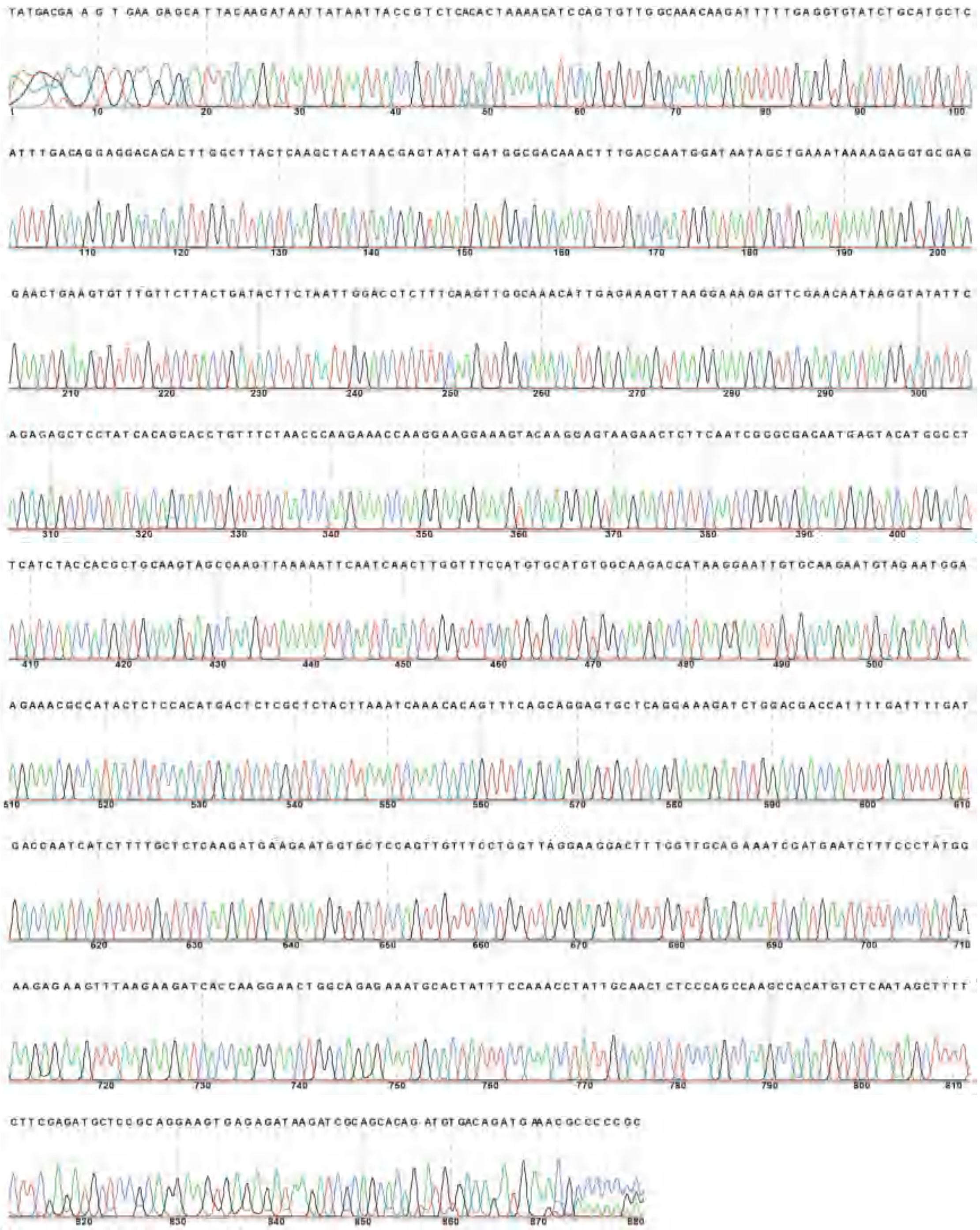

Mr-DH-V18 (447 bp)

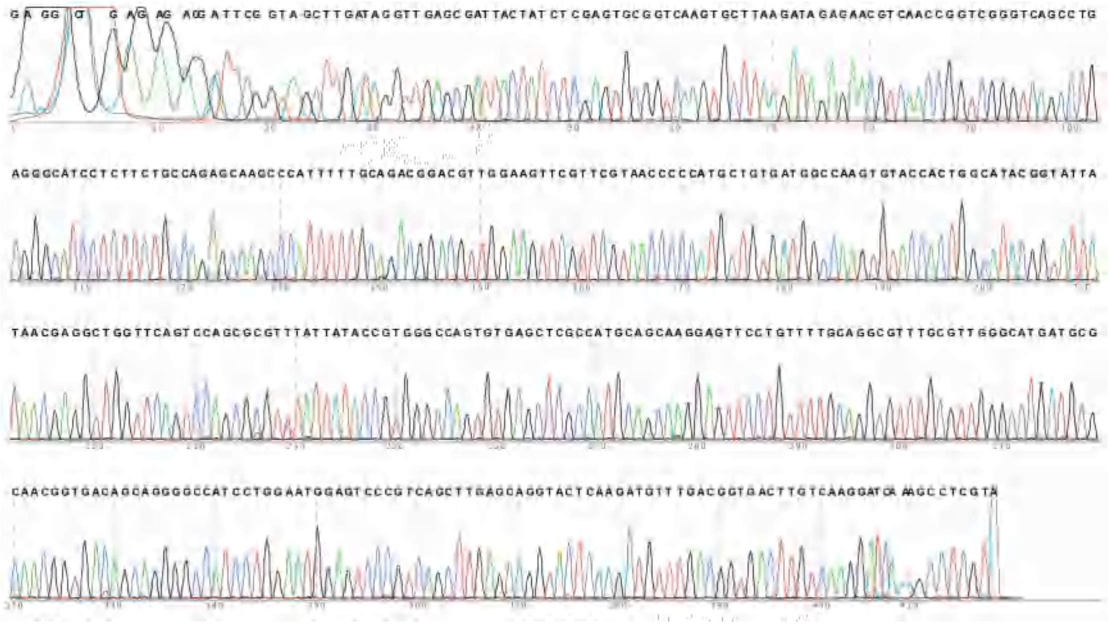

Mr-DH-V20 (415 bp)

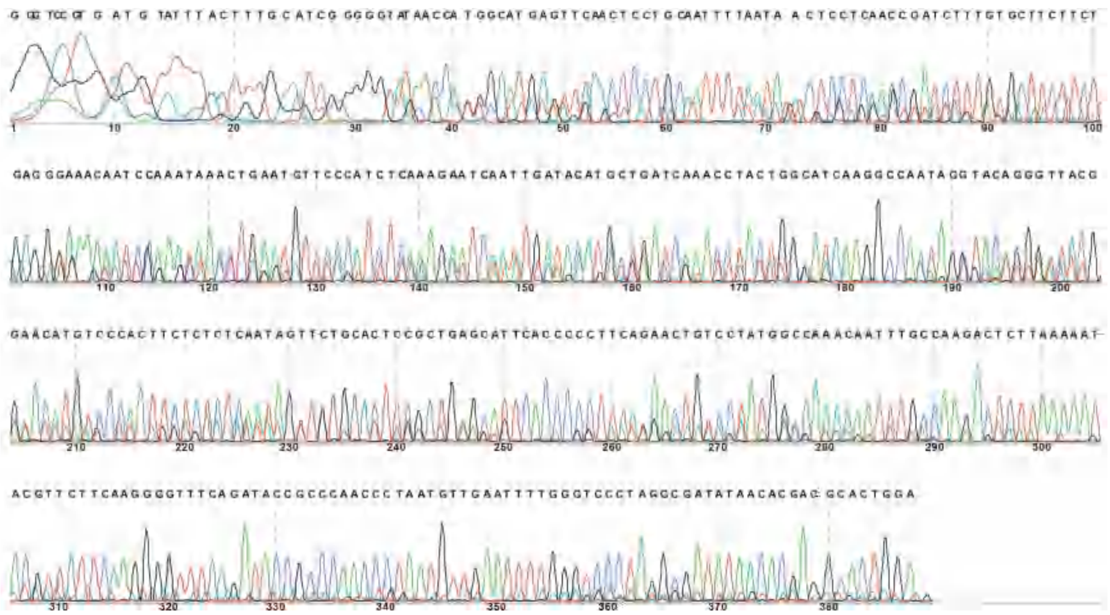

Mr-DH-V21 (613 bp in first PCR step)

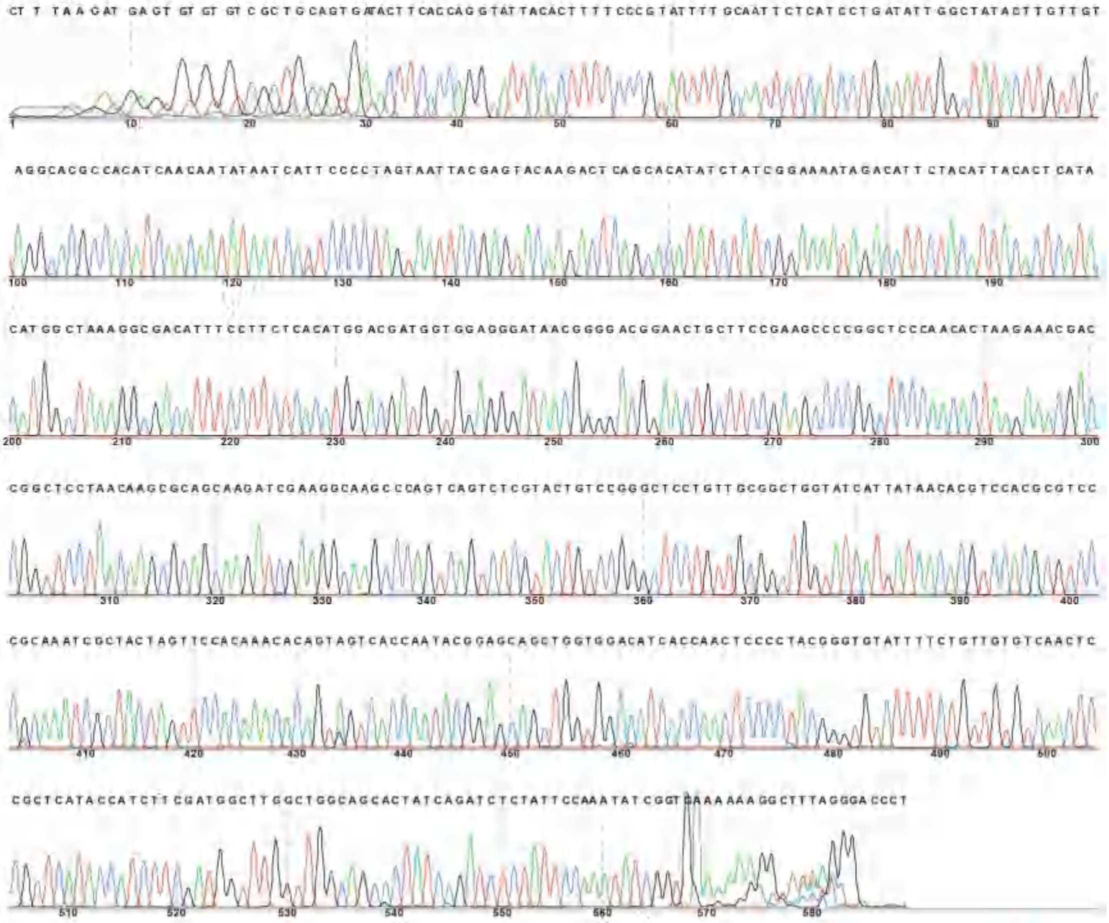

Mr-DH-V21 (412 bp in first PCR step)

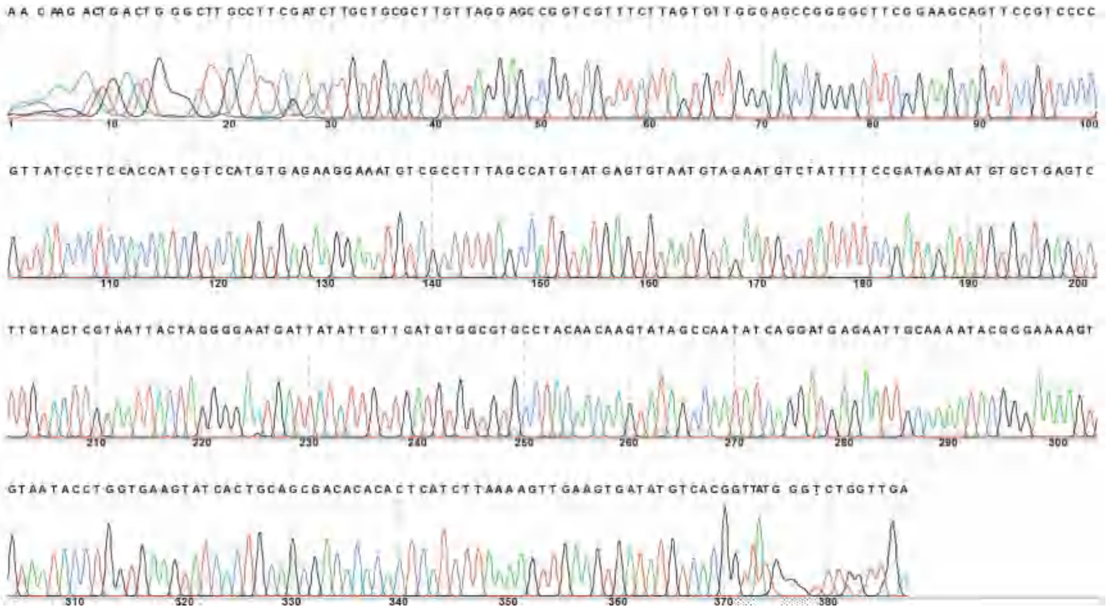

Mr-DH-V22 (579 bp)

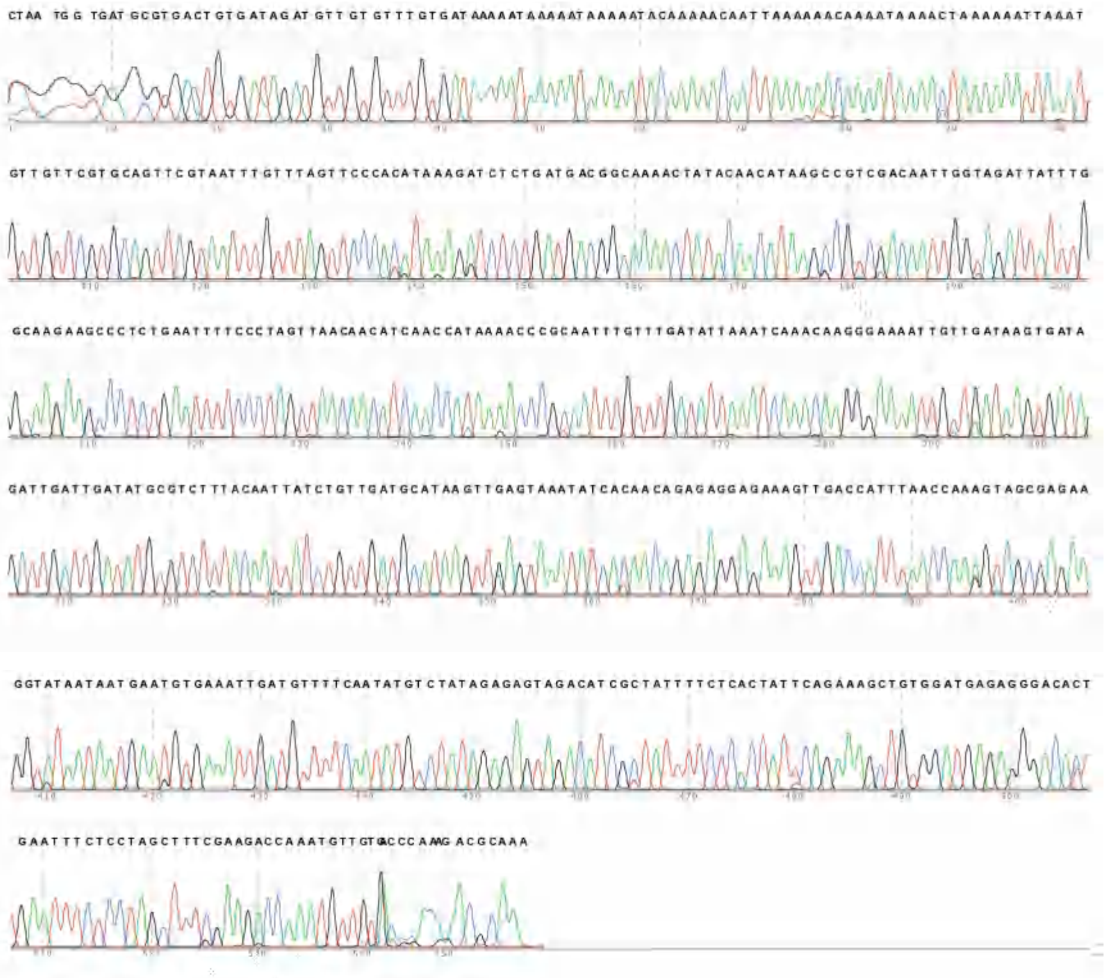

Mr-DH-V25 (532 bp)

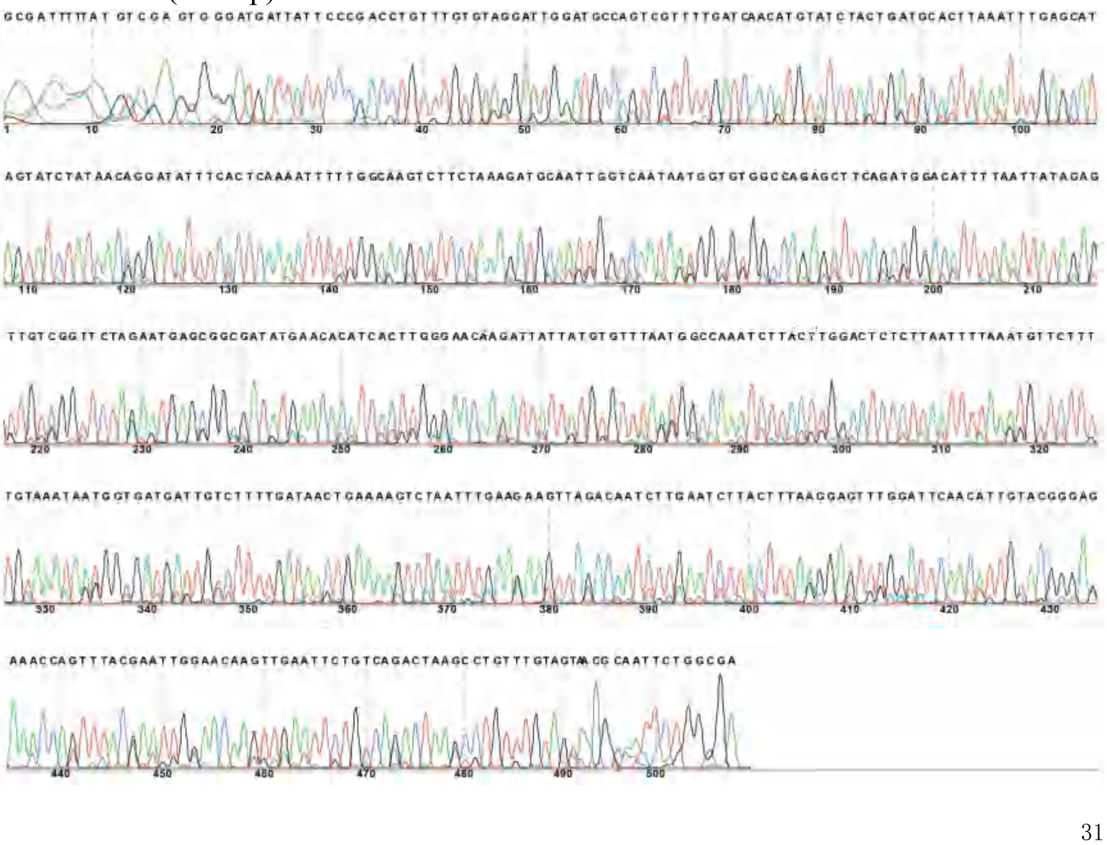

Mr-DH-V28 (456 bp)

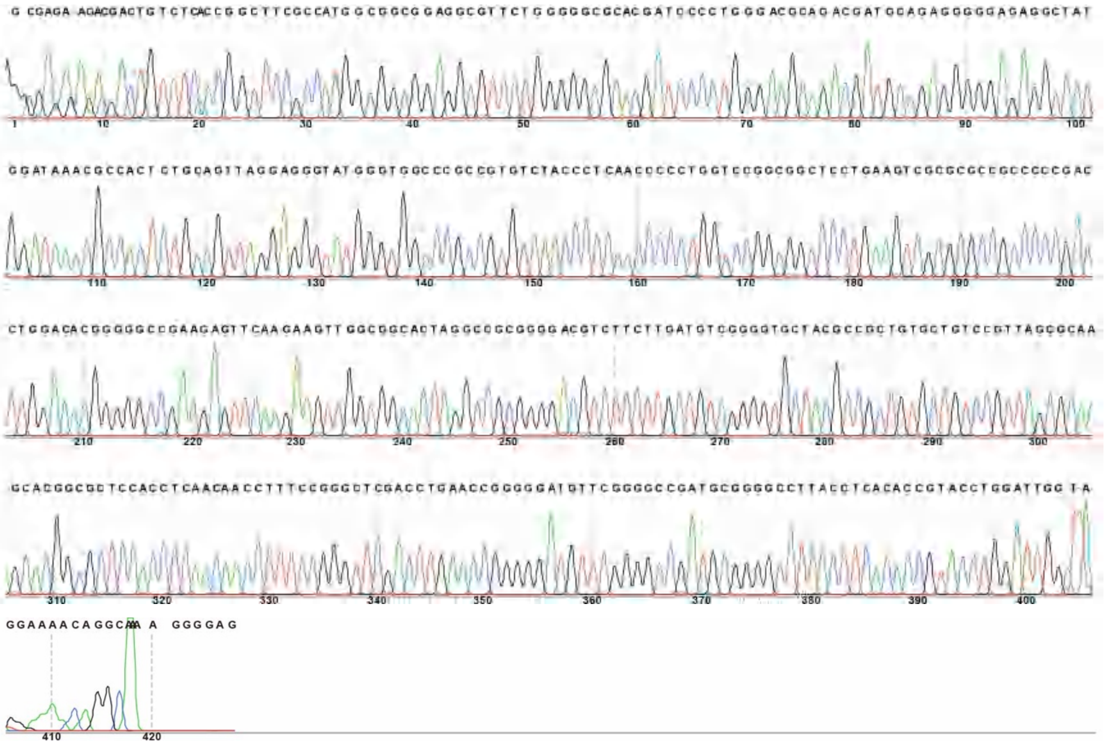

Mr-DH-V30 (424 bp)

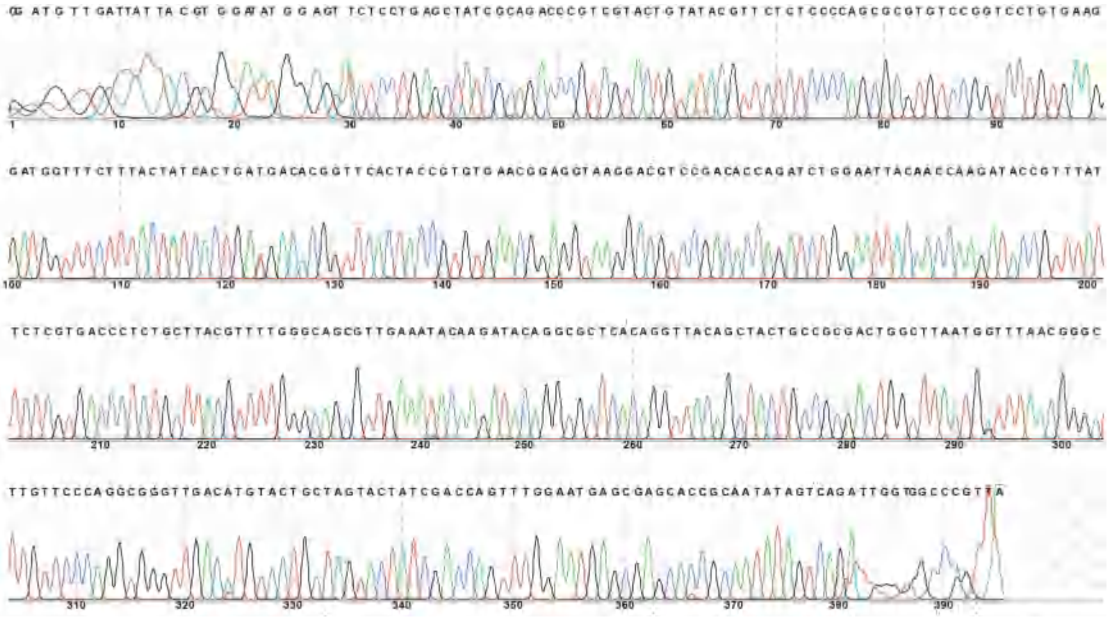

Macrobrachium rosenbergii virus 12, MRV12 (717 bp)

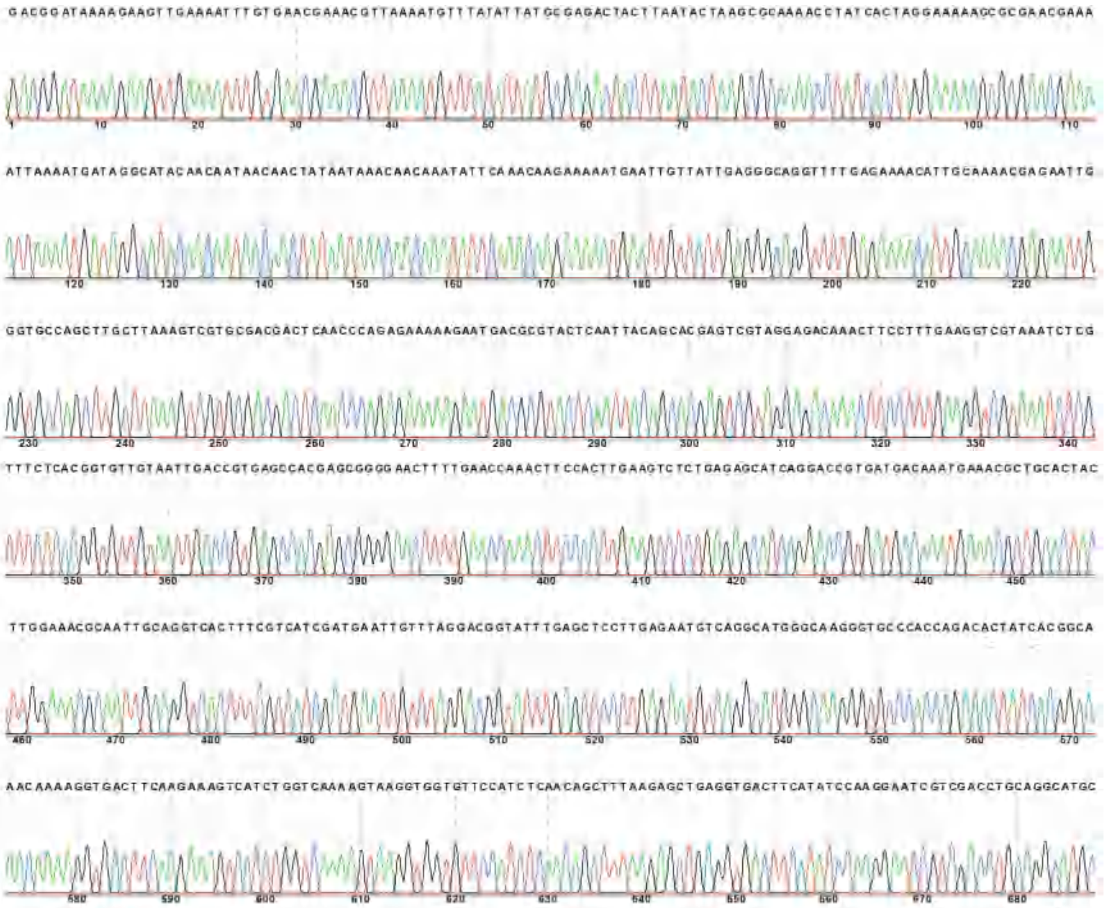

Mr-DH-V38 (522 bp)

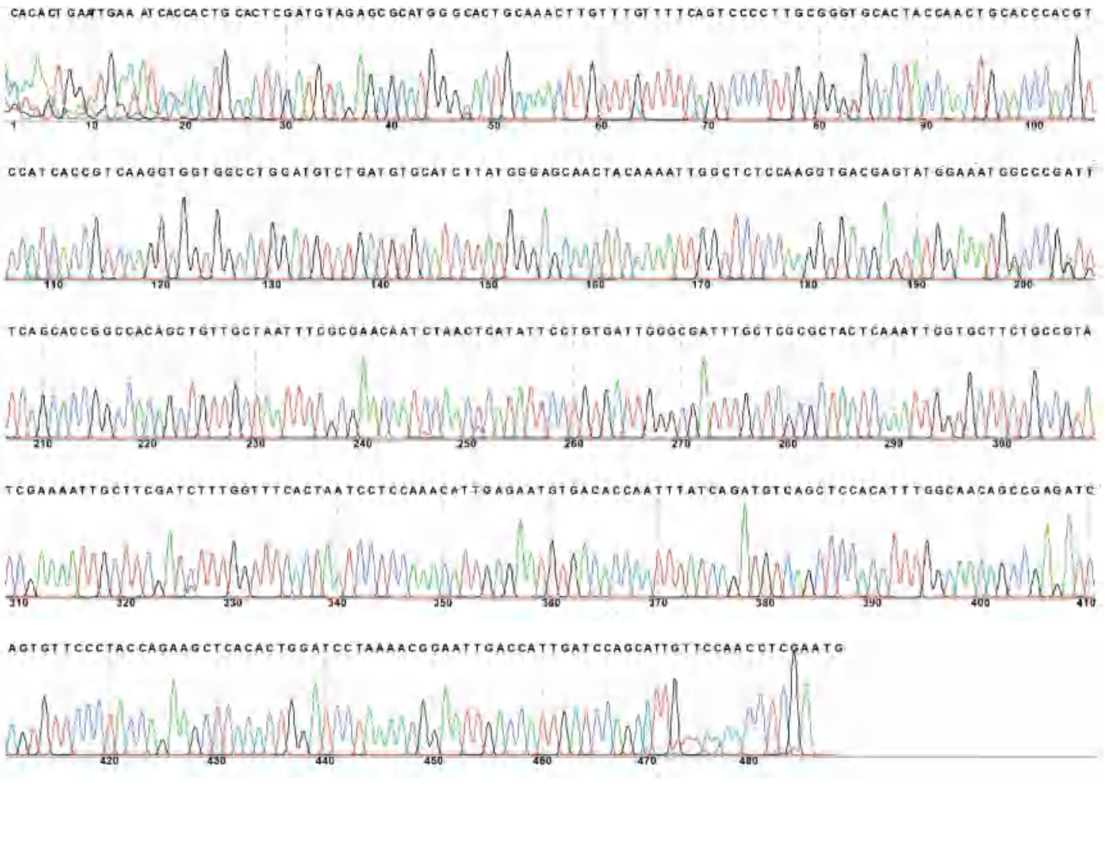

Mr-DH-V40 (502 bp)

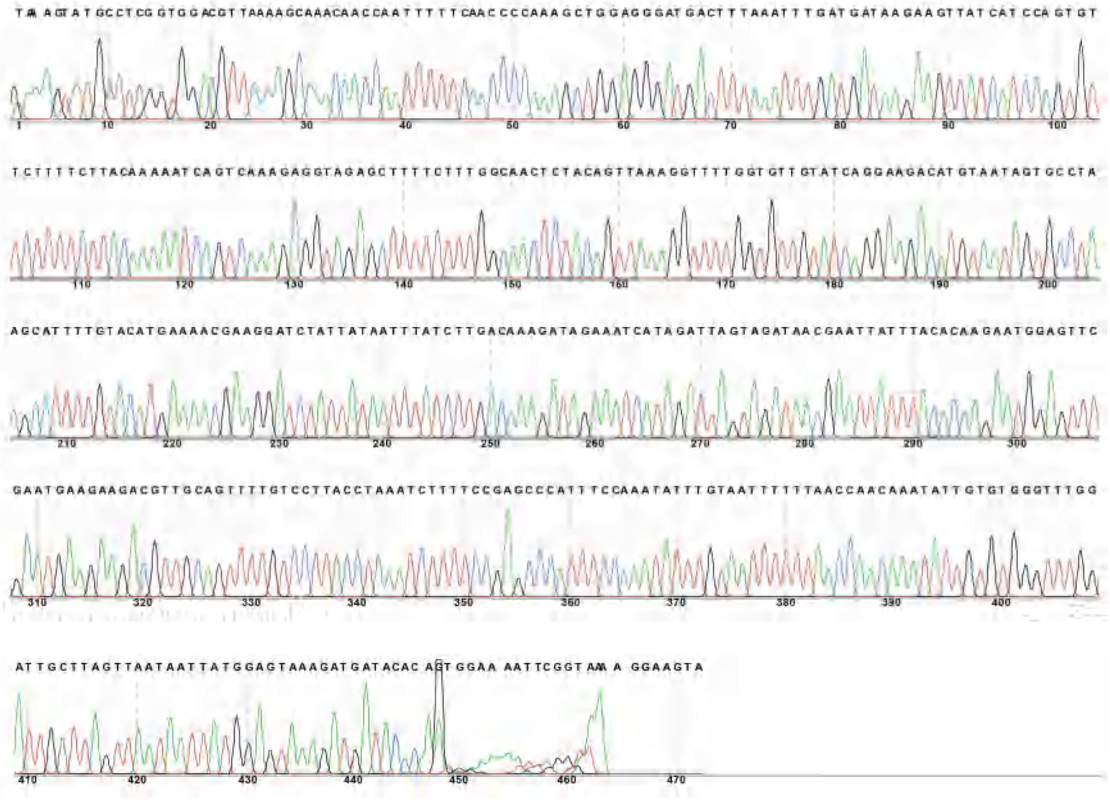

Mr-DH-V42(507bp)

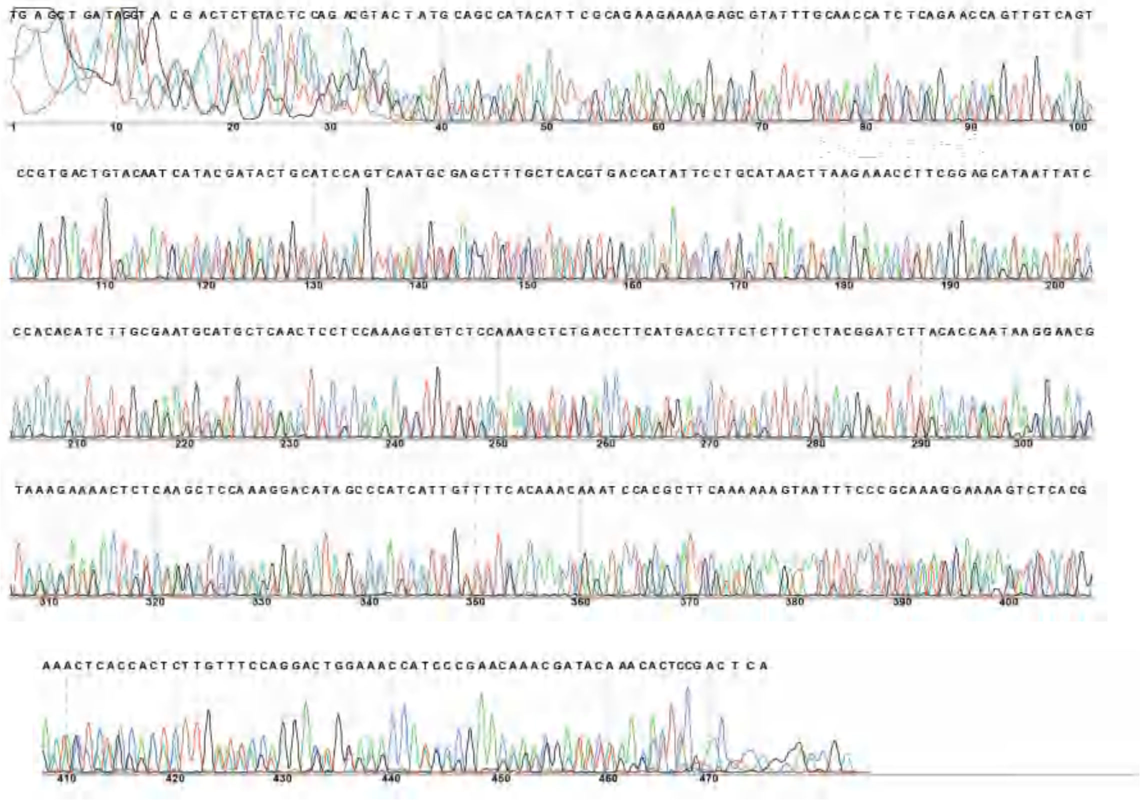

Mr-DH-V50 (386 bp)

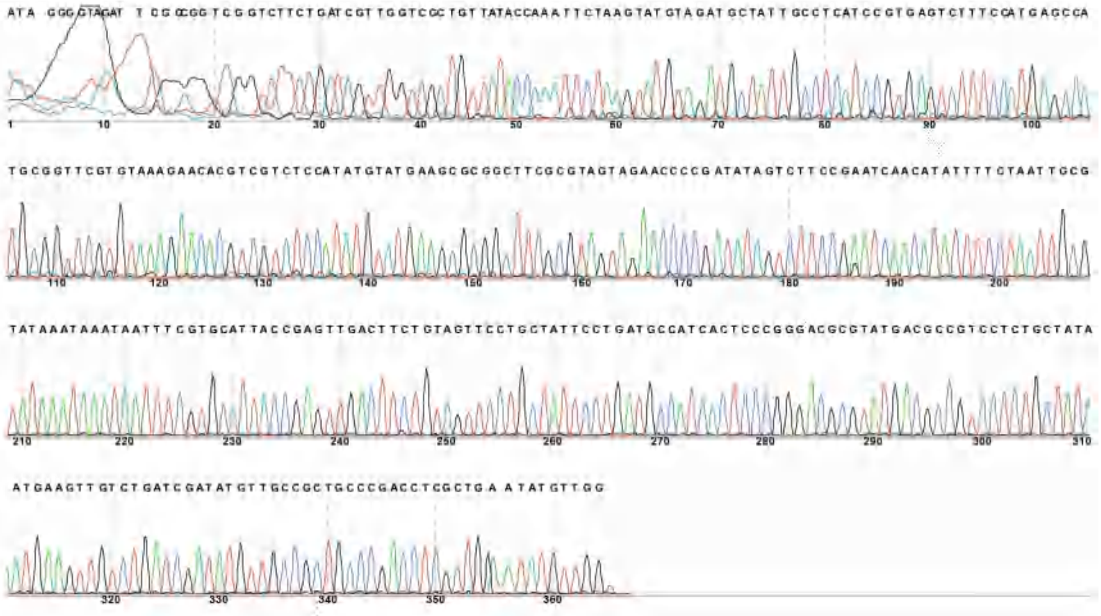

Mr-H-V1 (427 bp)

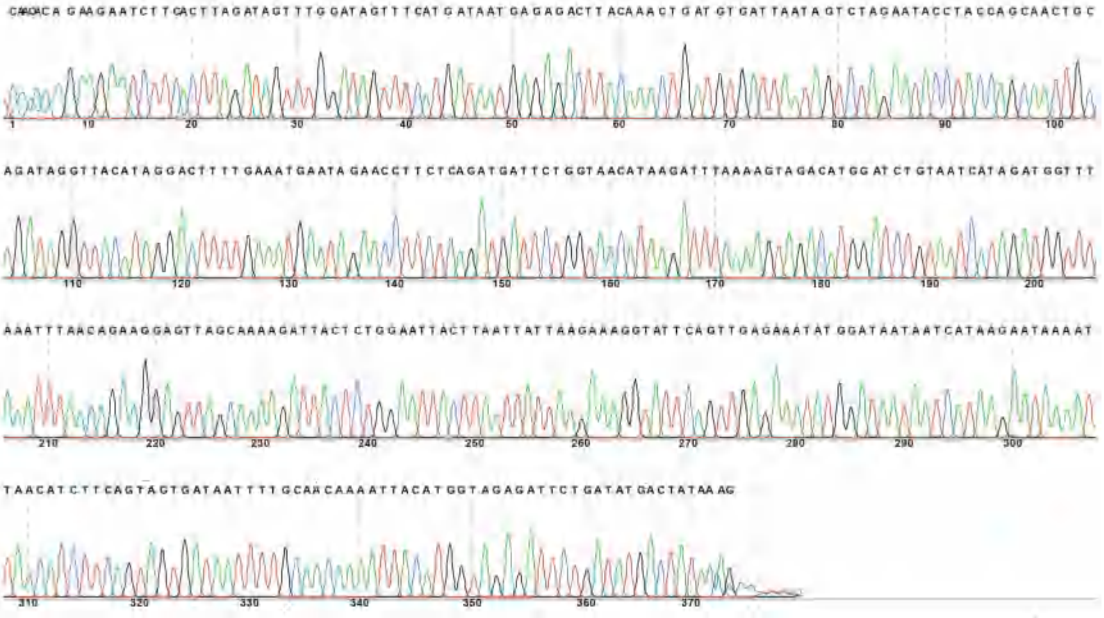

Mr-H-V2 (436 bp)

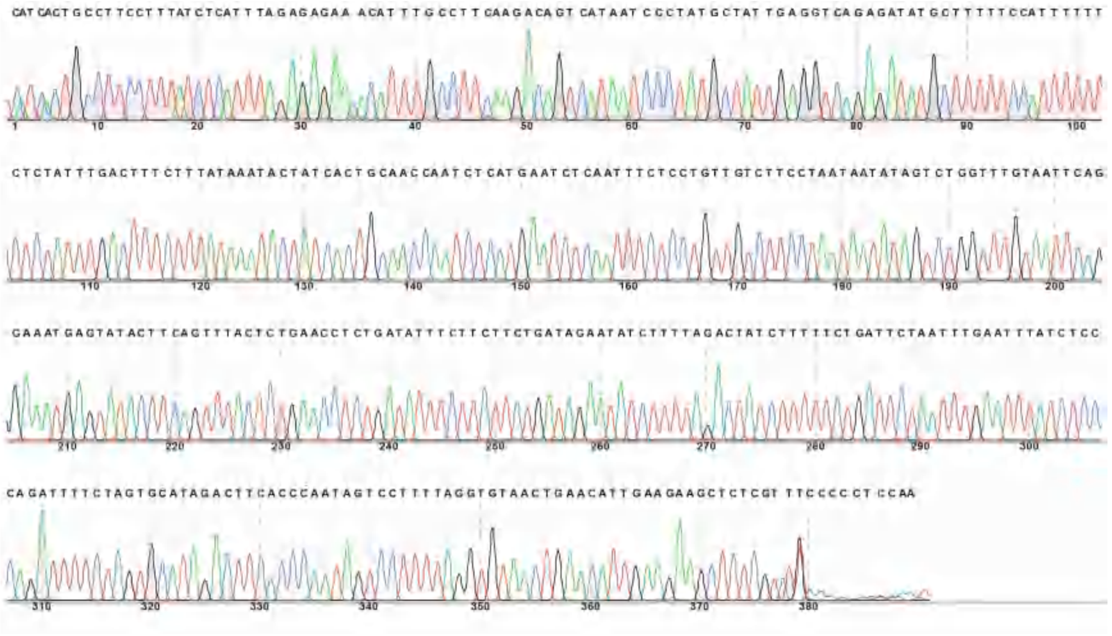

Mr-H-V8 (527 bp)

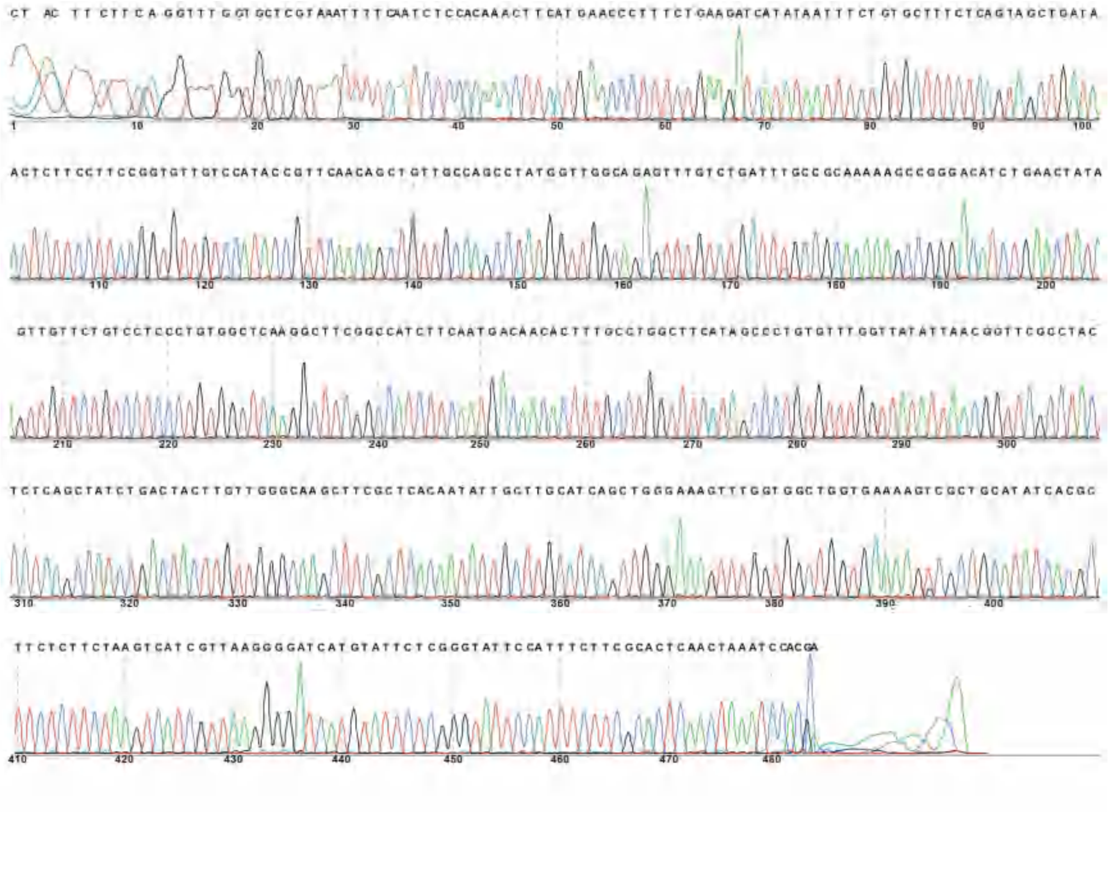

Mr-H-V10 (486 bp)

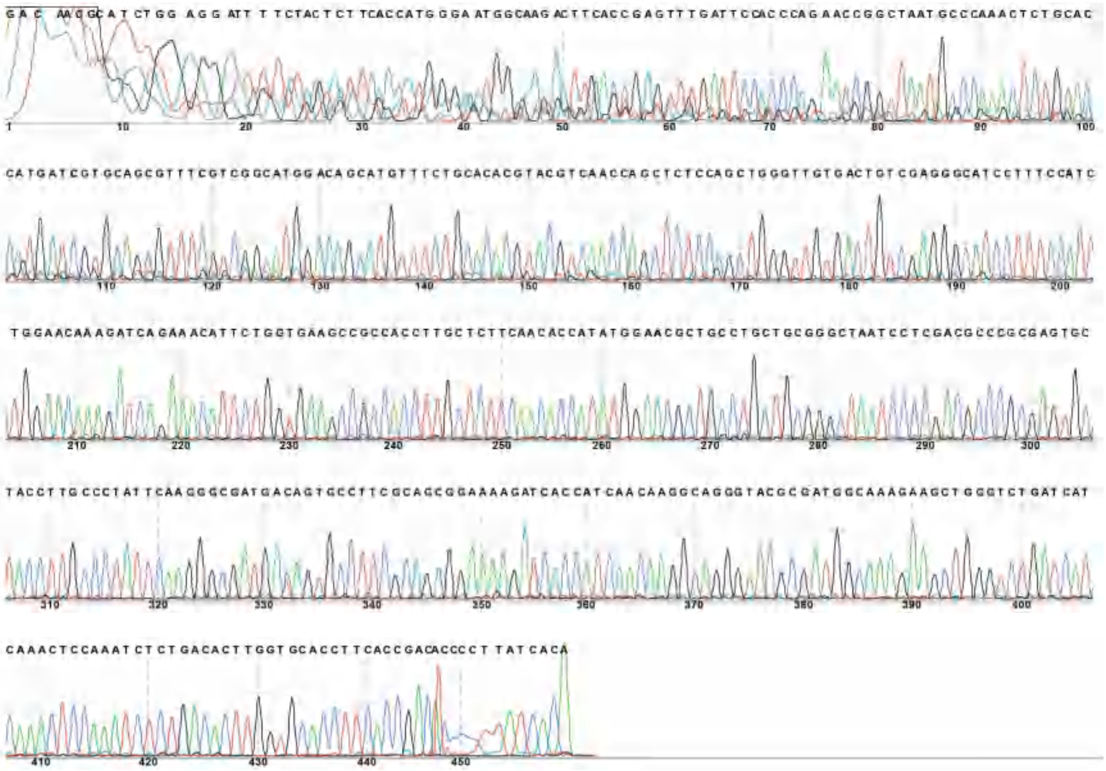

Mr-H-V14 (441 bp)

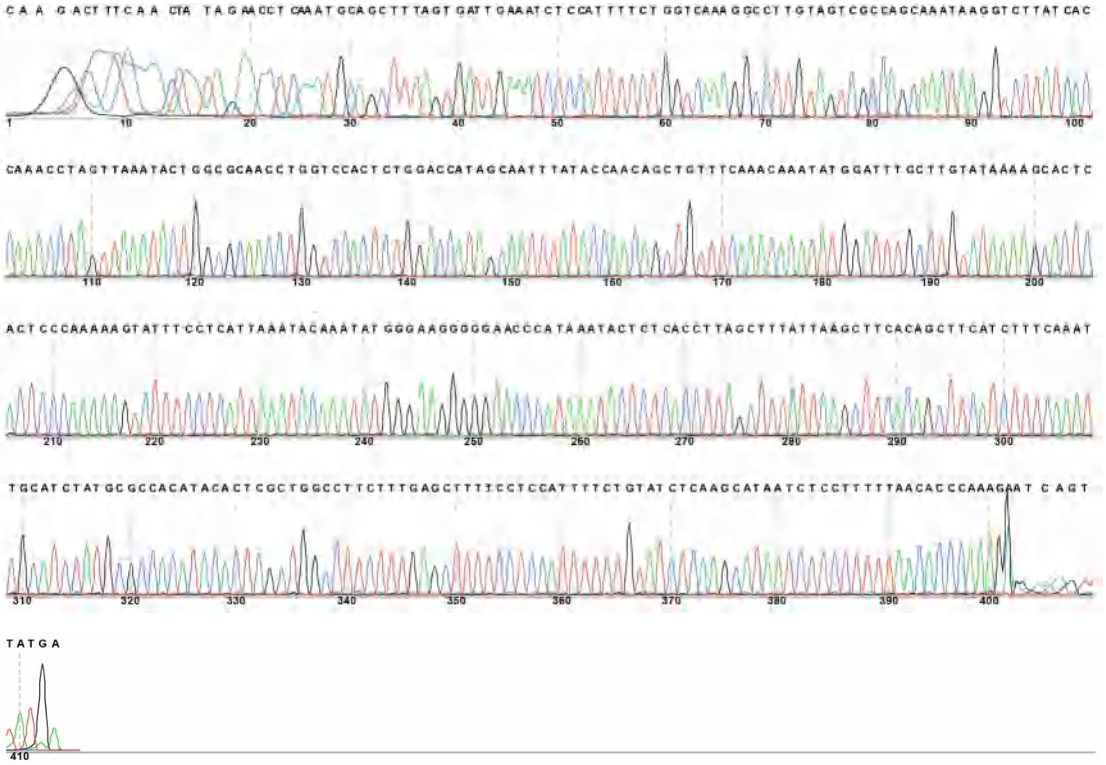

Mr-H-V25 (203 bp)

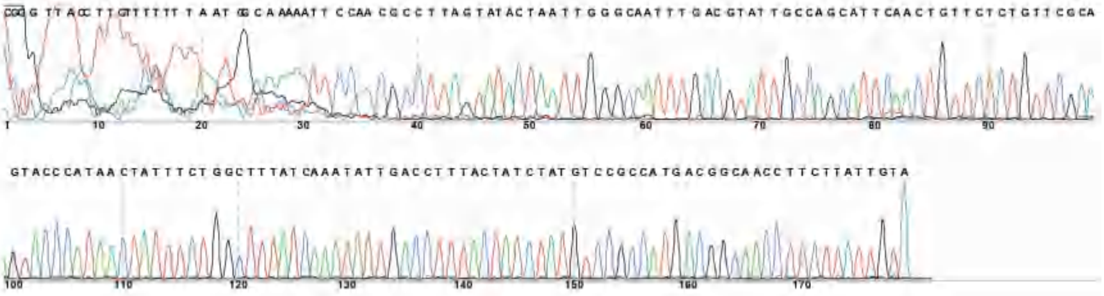

Mr-H-V32 (566 bp)

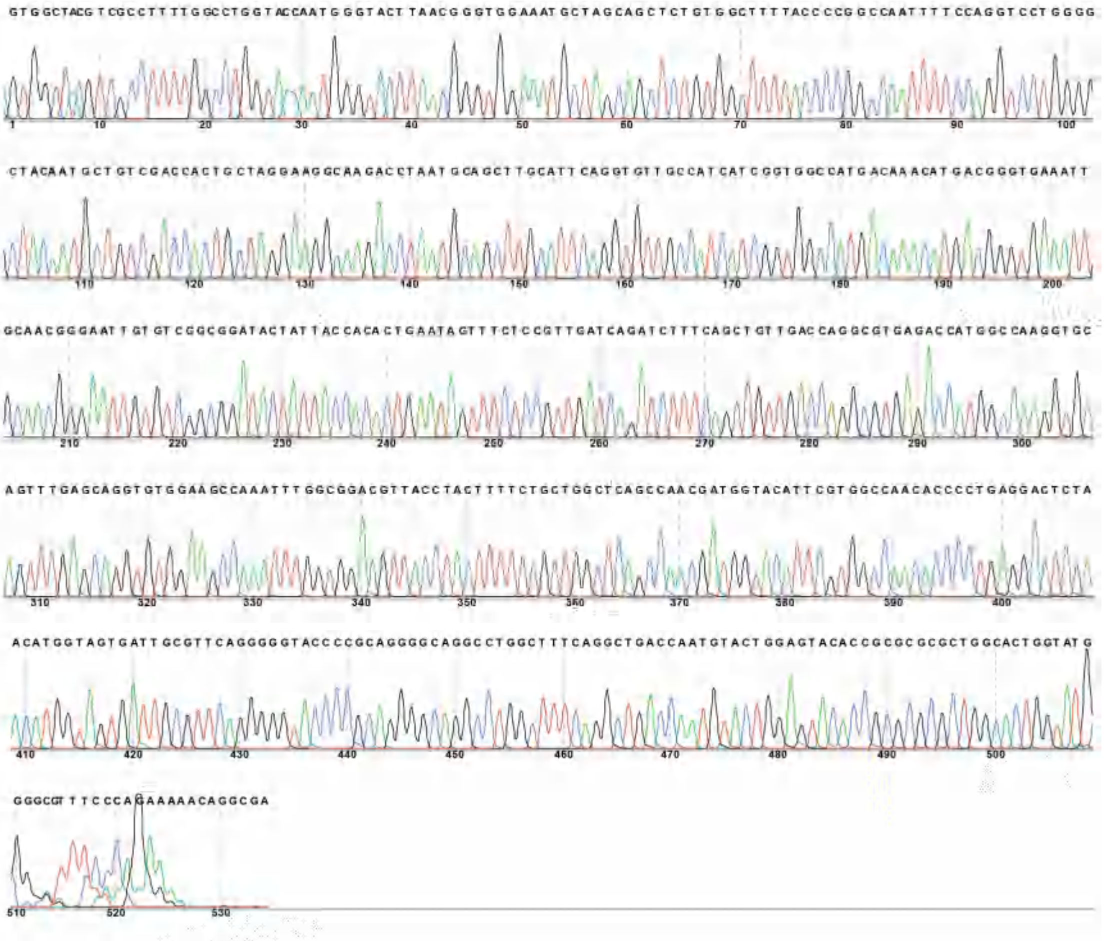

Mr-H-V33 (392 bp)

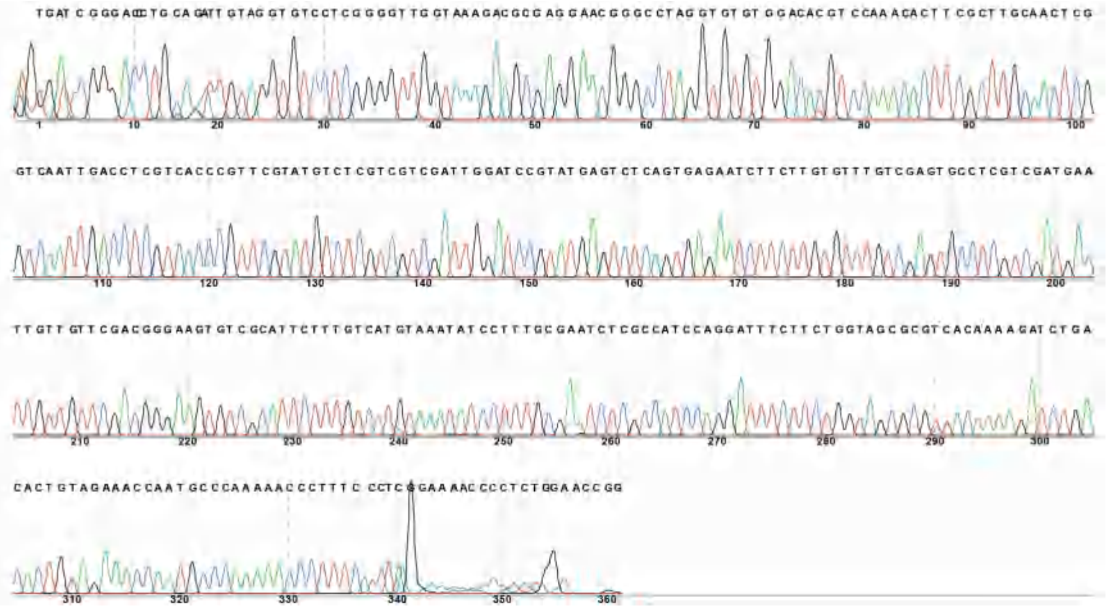

Mr-H-V35(410bp)

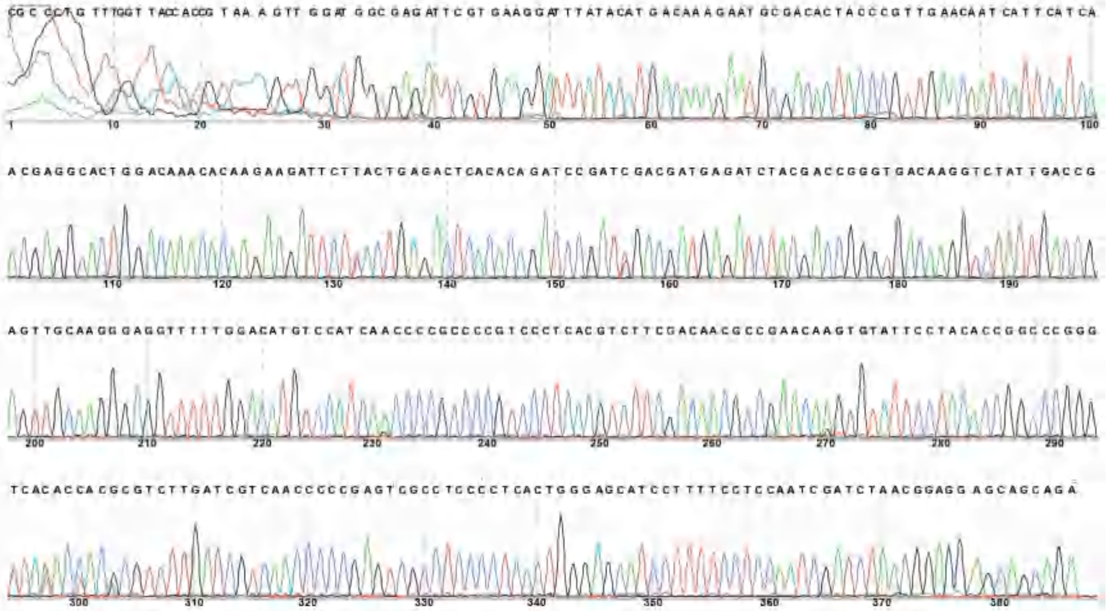

Mr-H-V36 (217 bp)

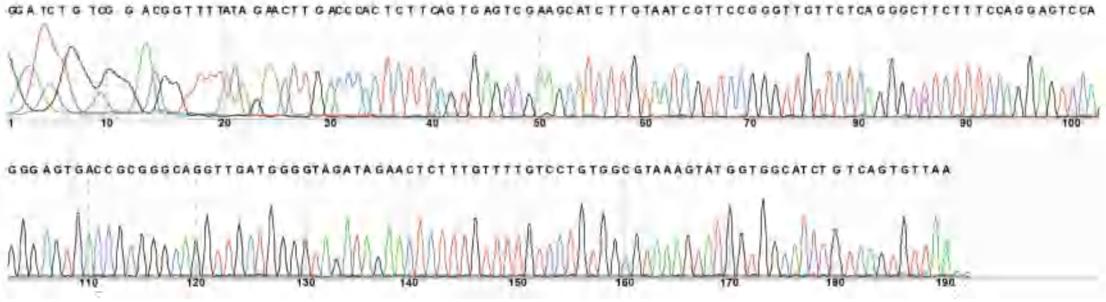

Mr-H-V38(384bp)

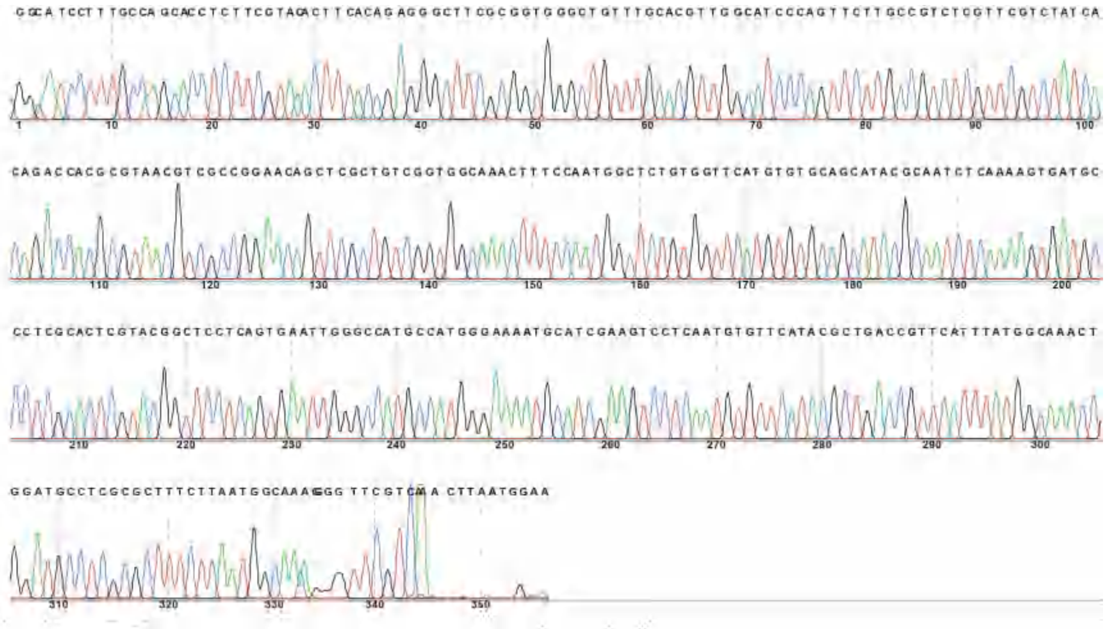

Mr-H-V39 (221 bp)

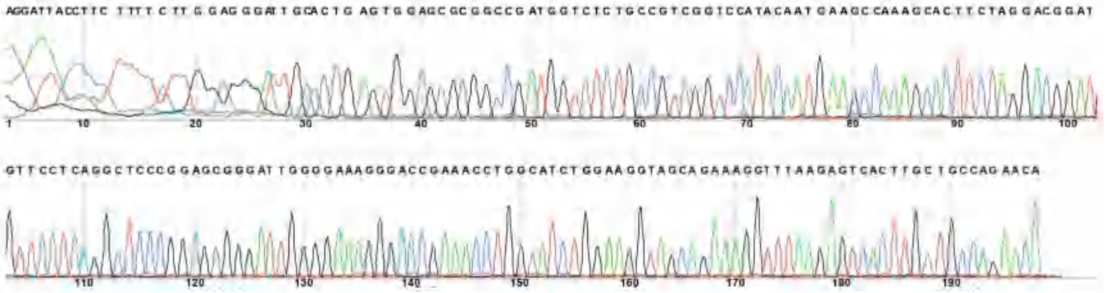

Mr-H-V40 (648 bp in the first PCR step)

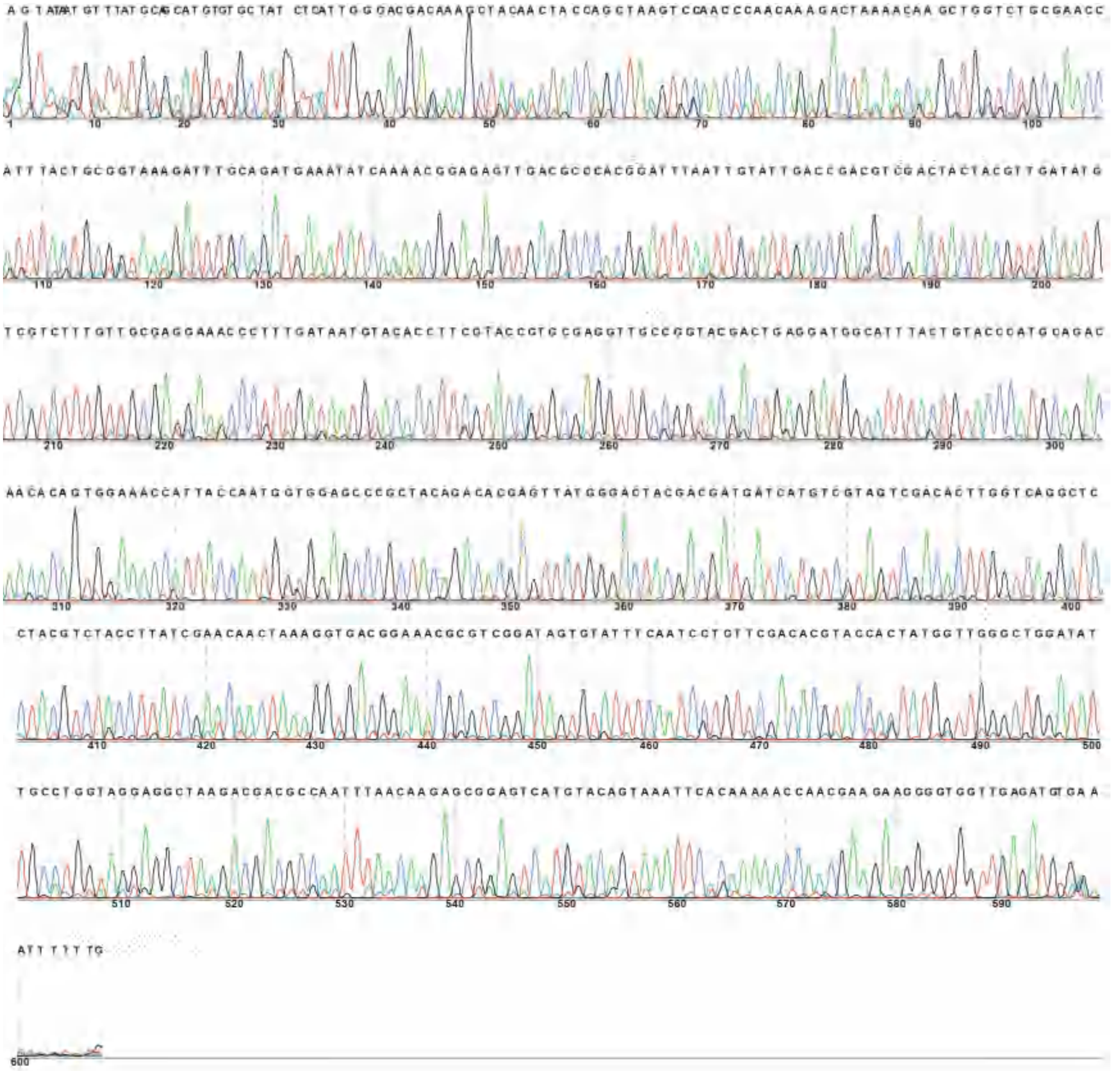

Mr-H-V40 (552 bp in the second PCR step)

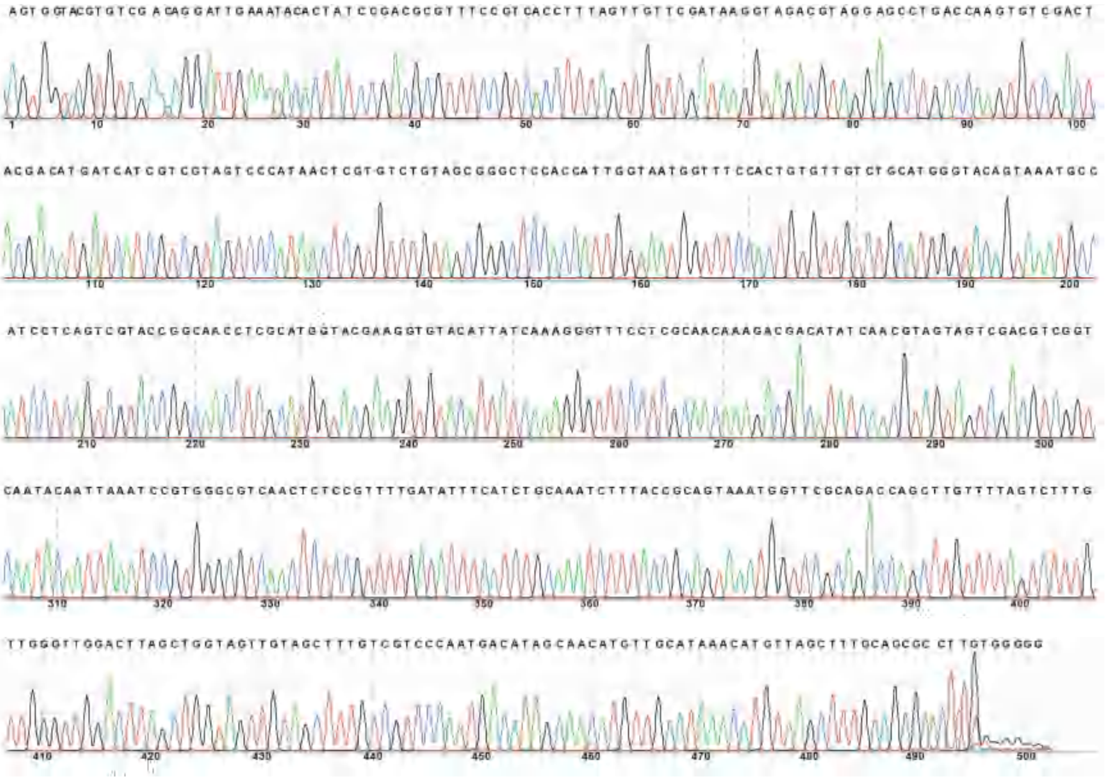

Mr-H-V45 (242 bp)

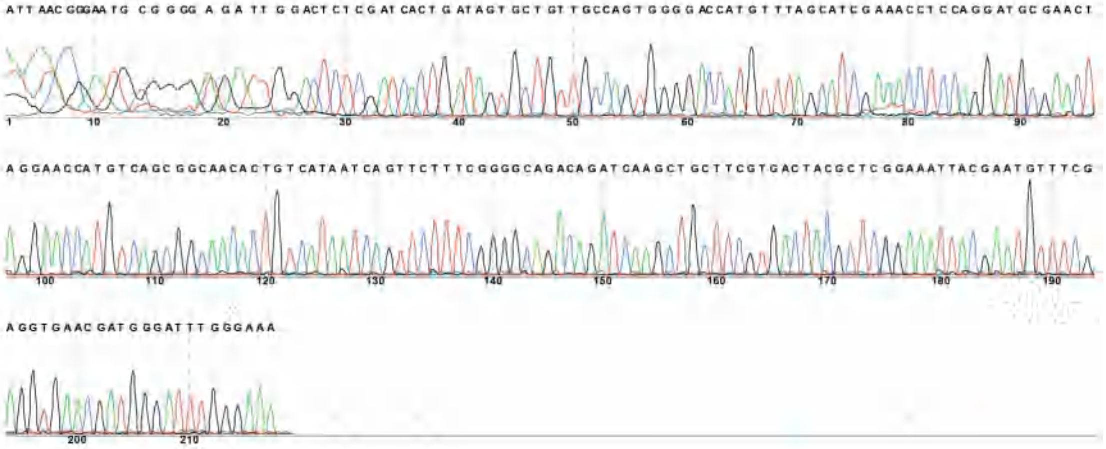

Mr-H-V46 (262 bp)

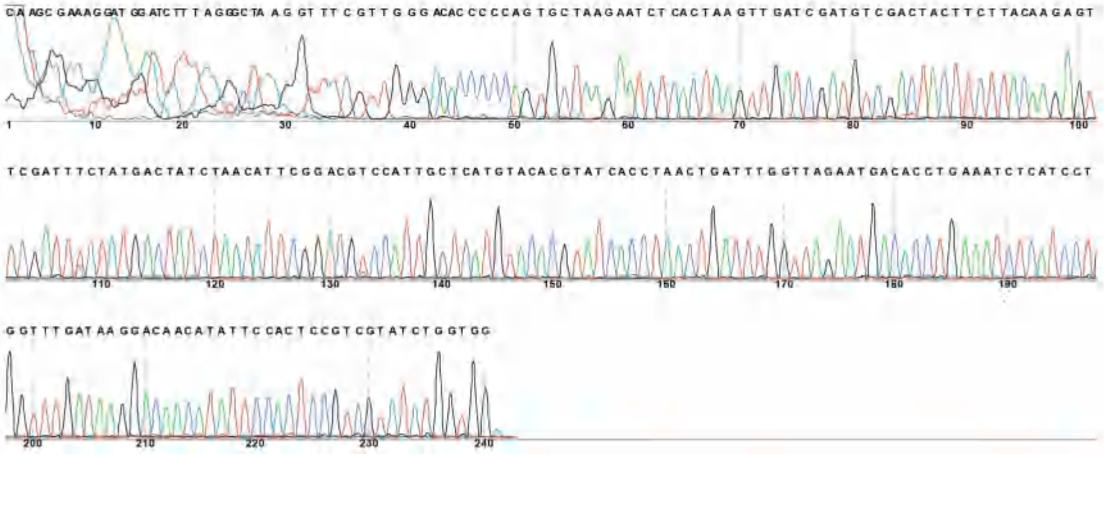

Mr-H-V60 (434 bp)

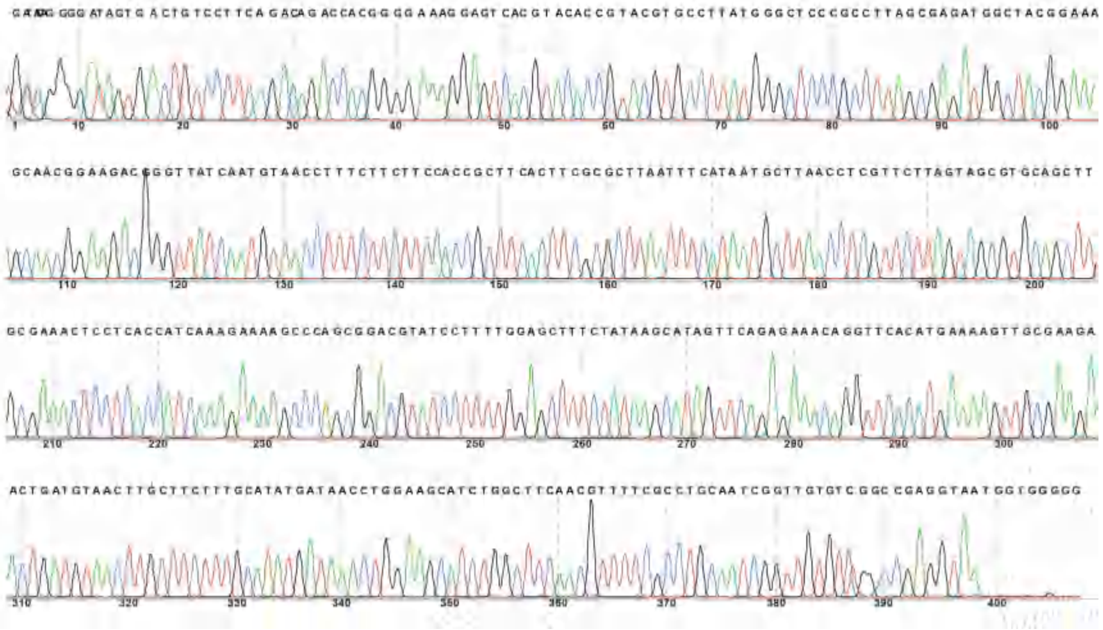

Mr-H-V80 (484 bp)

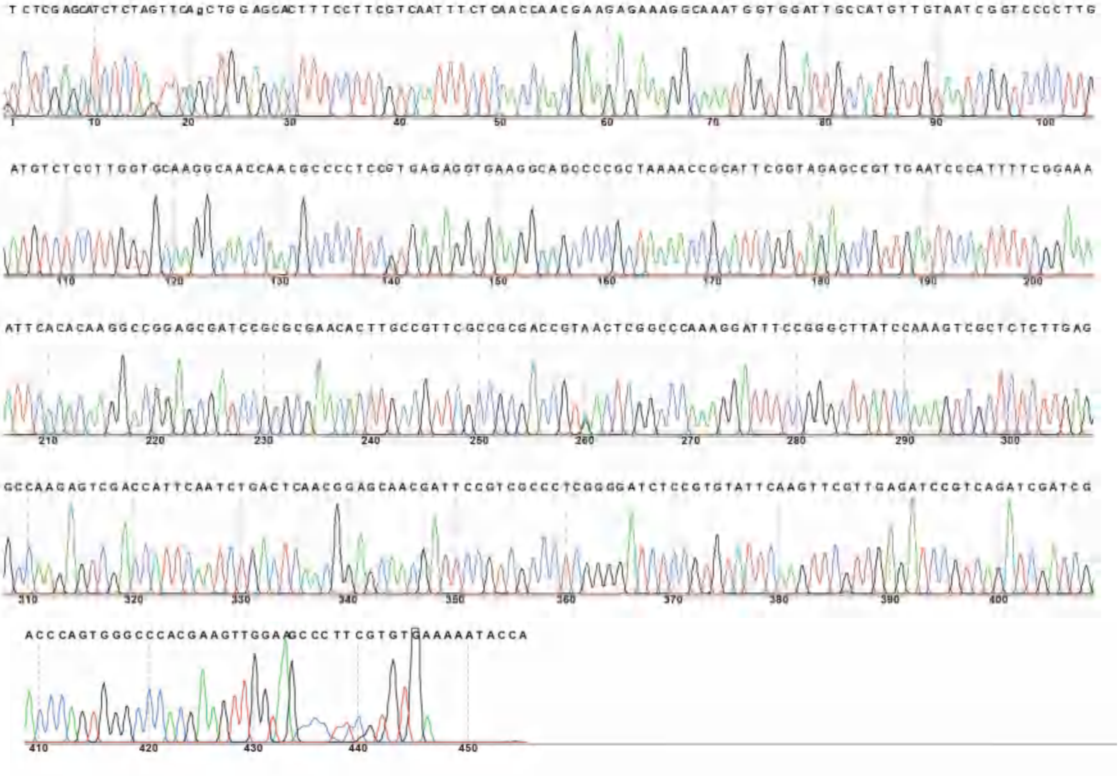

Mr-H-V82 (432 bp)

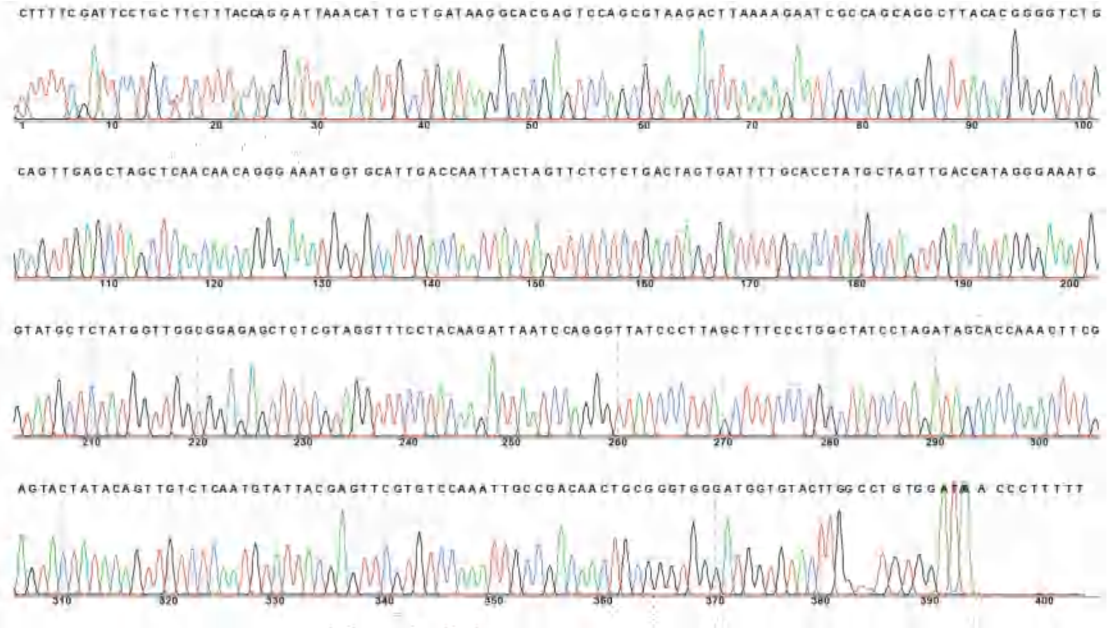

Mr-H-V85 (429 bp)

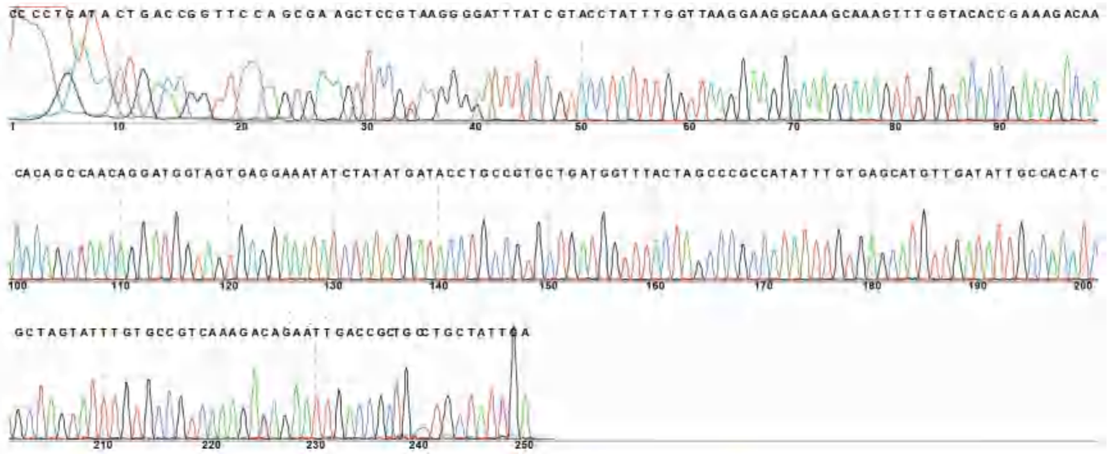

Mr-H-V87 (345 bp)

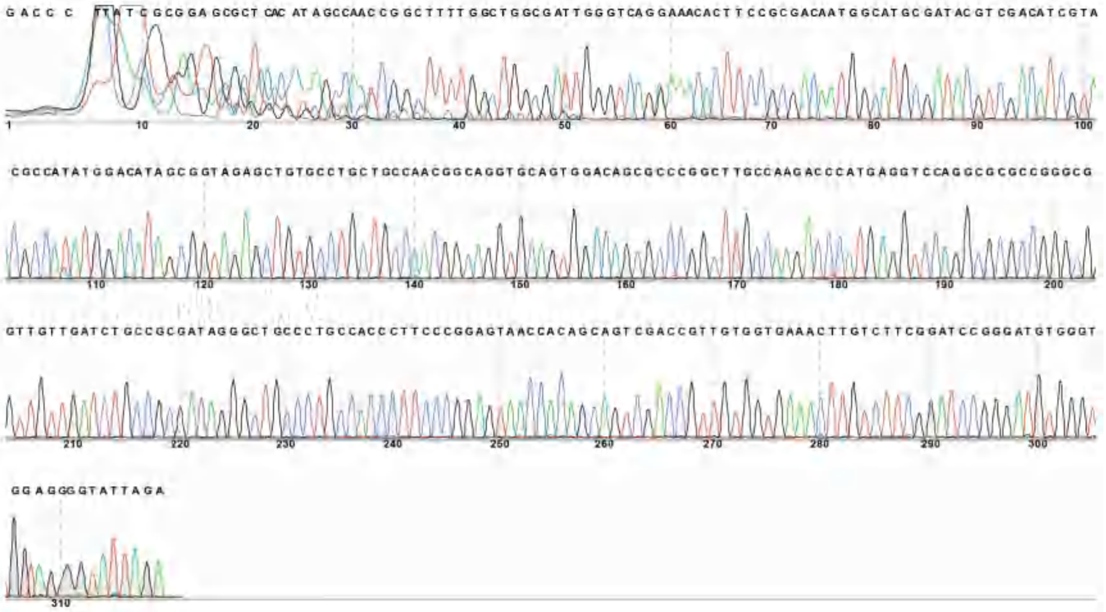

Mr-H-V89 (335 bp)

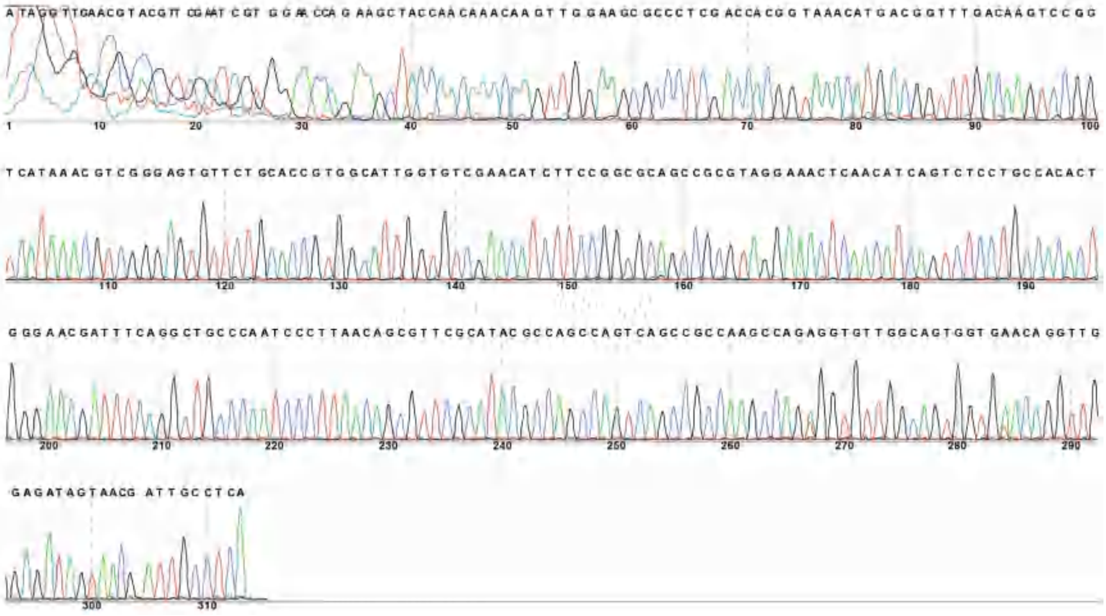

Mr-H-V93 (443 bp)

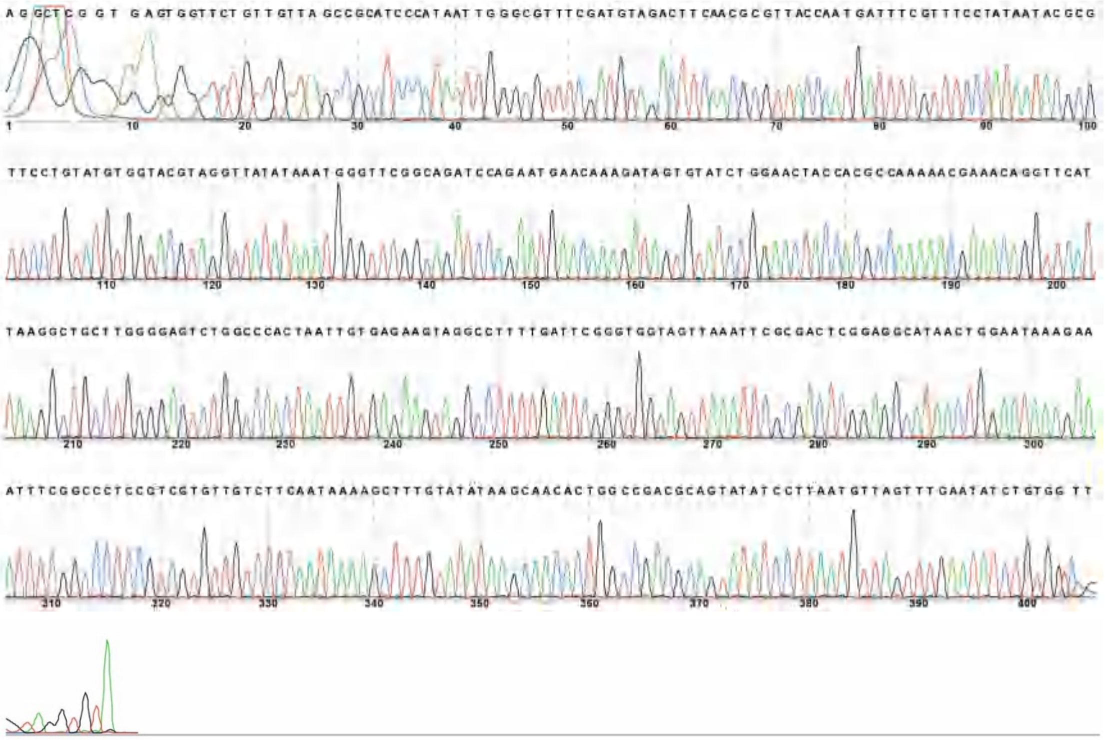

Mr-H-V95 (330 bp)

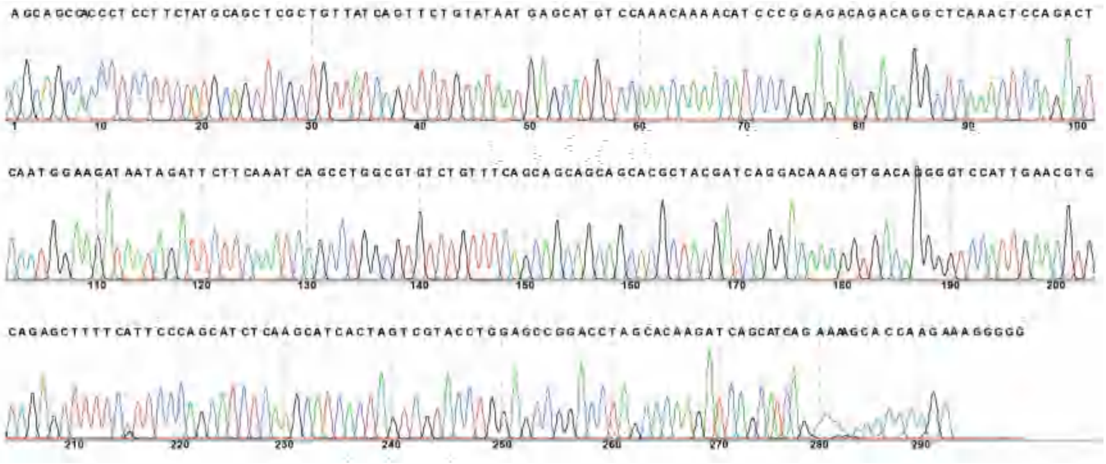

Mr-H-V99 (372 bp)

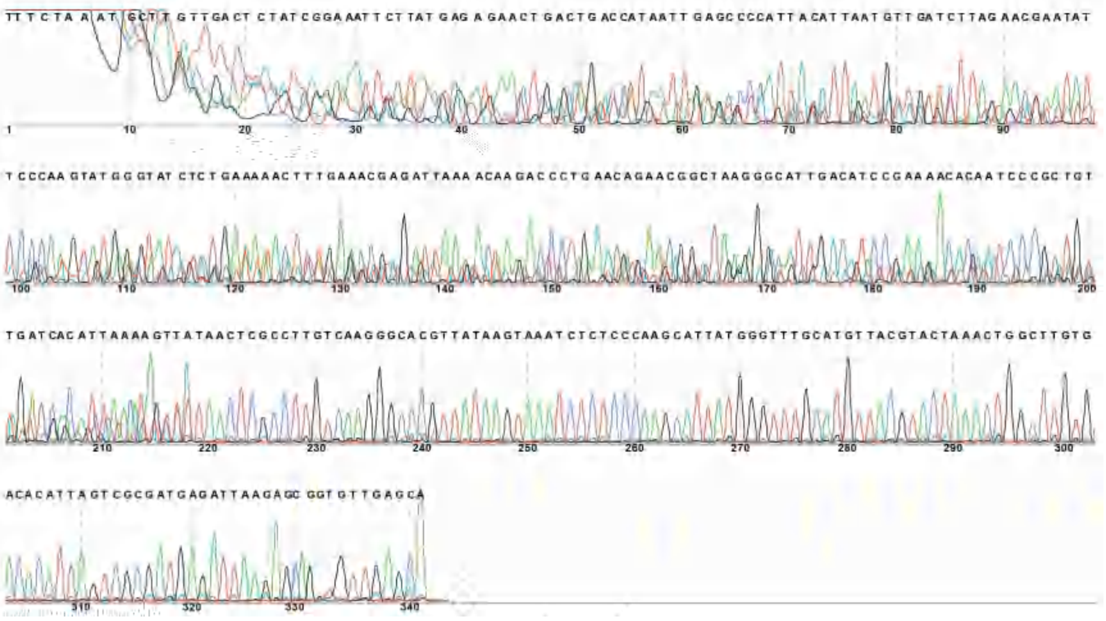

Mr-H-V102 (235 bp)

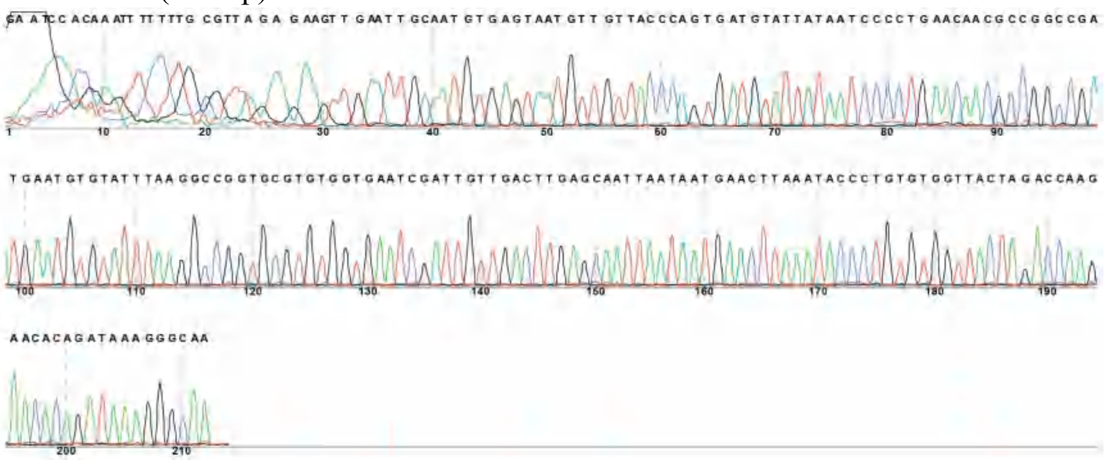

Mr-H-V105 (250 bp)

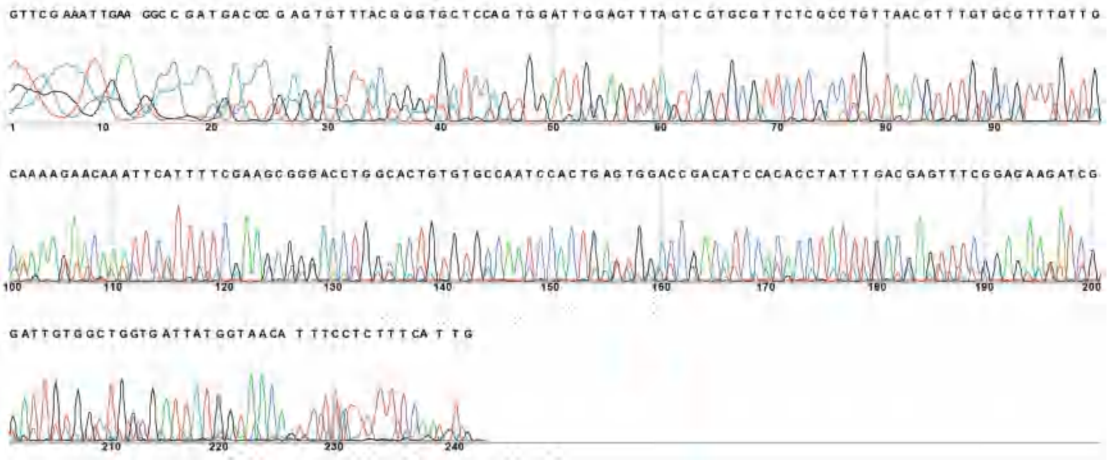

Mr-H-V107 (436 bp)

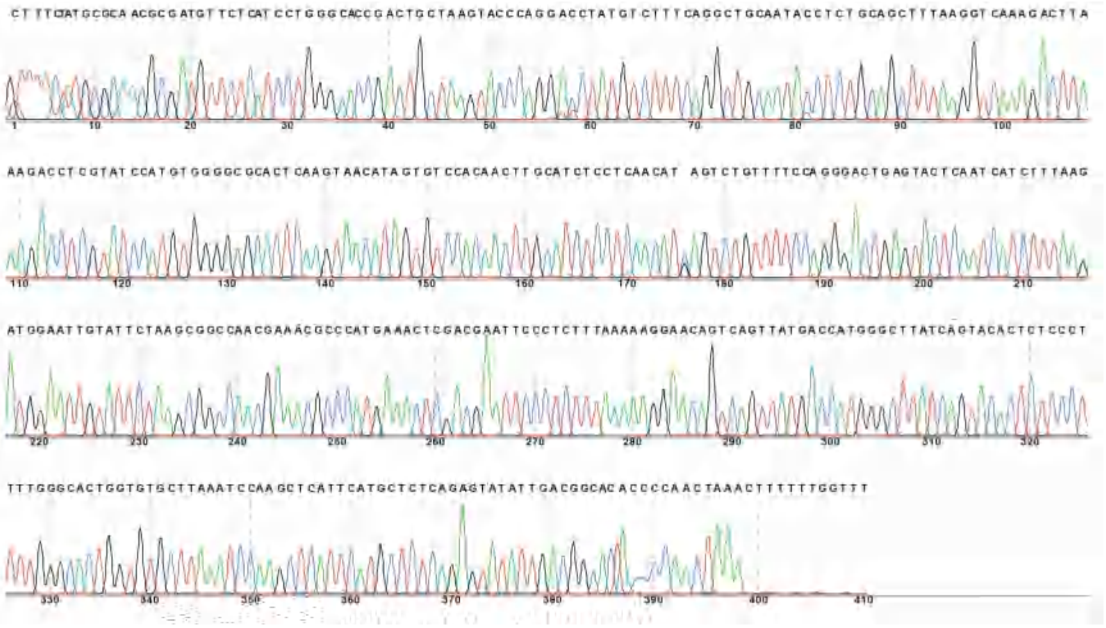

Mr-H-V109 (603 bp in the first PCR step)

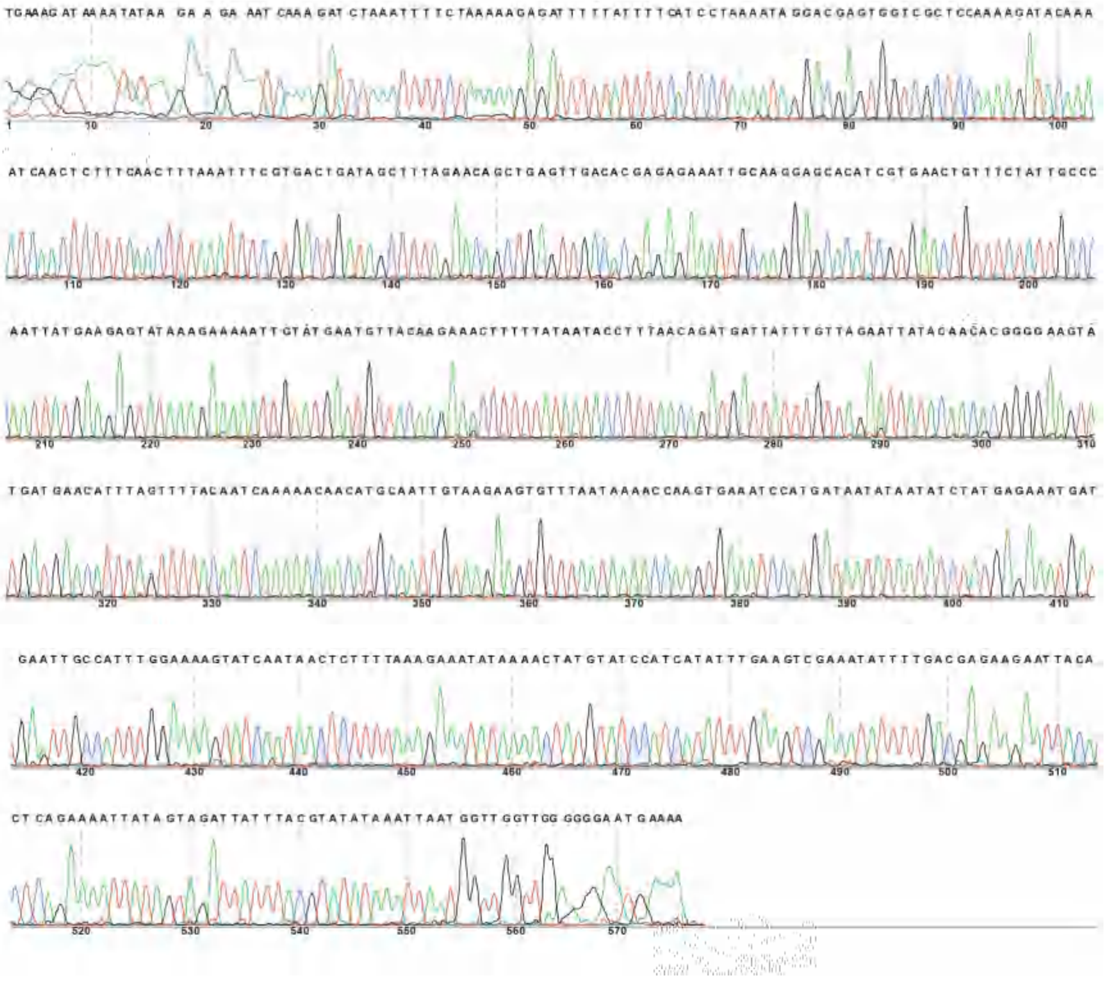

Mr-H-V109 (598 bp in the second PCR step)

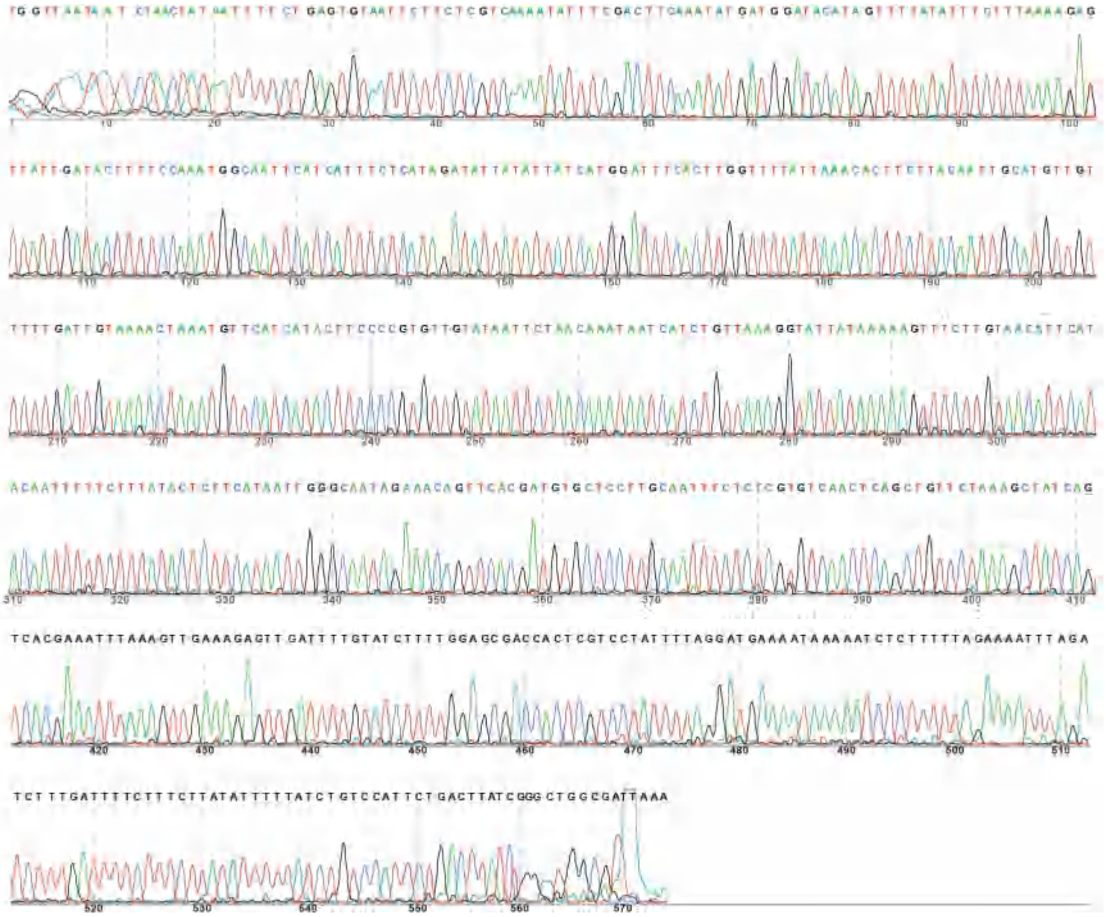

Mr-H-V110 (475 bp)

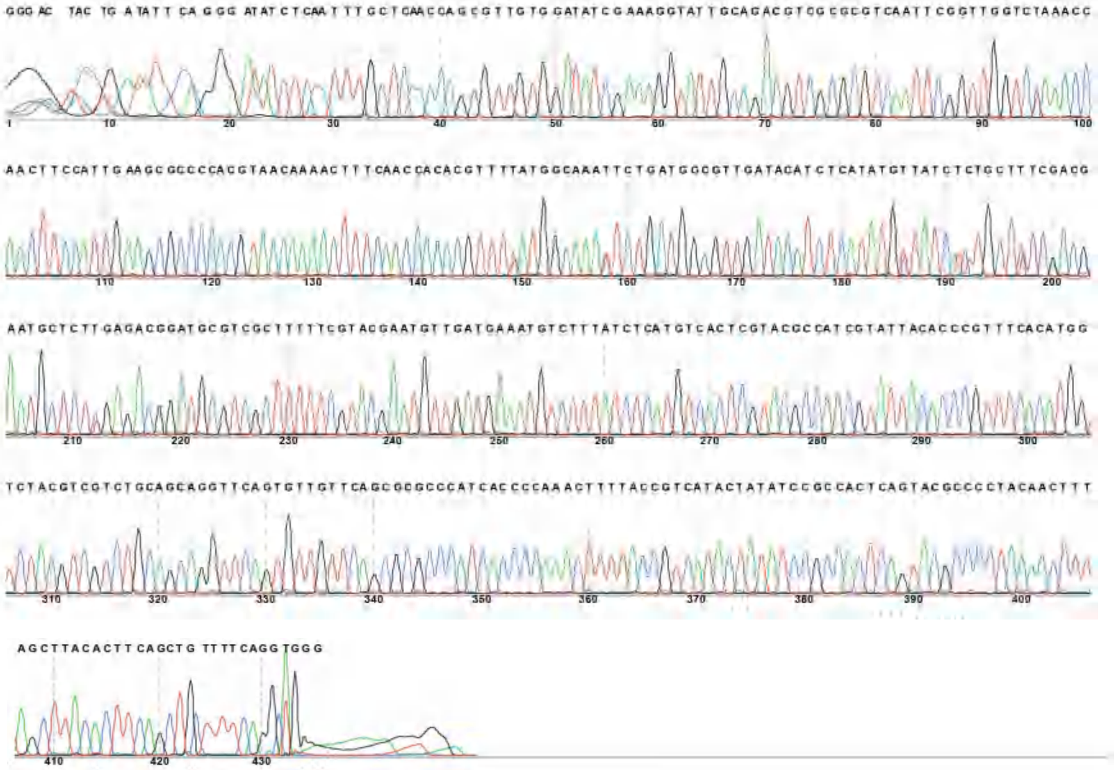

Mr-H-V111 (405 bp)

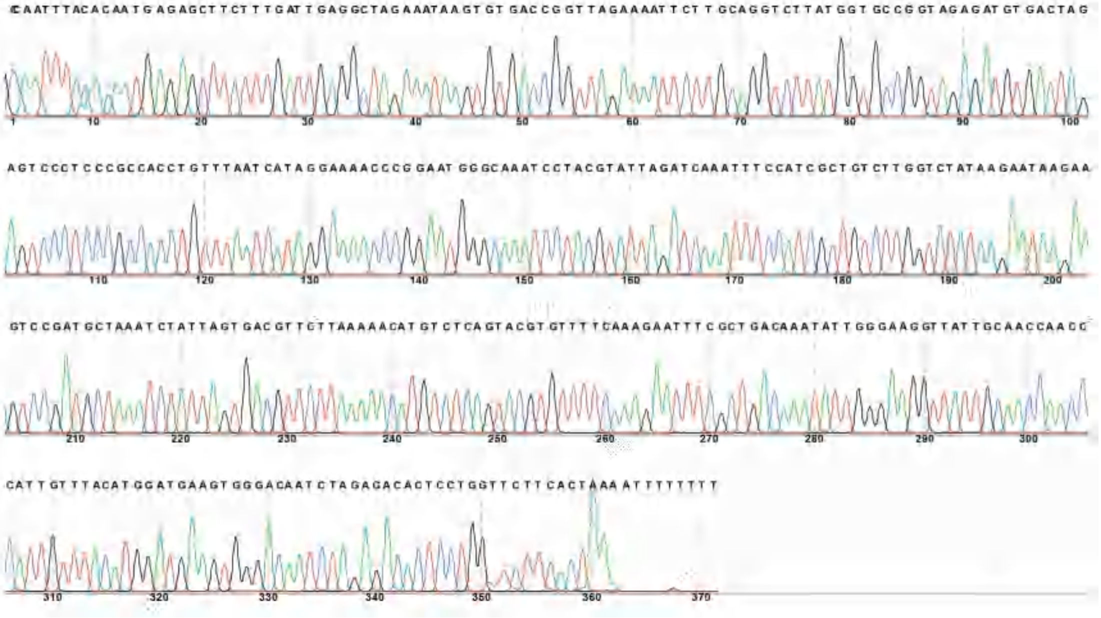

Mr-H-V113 (455 bp)

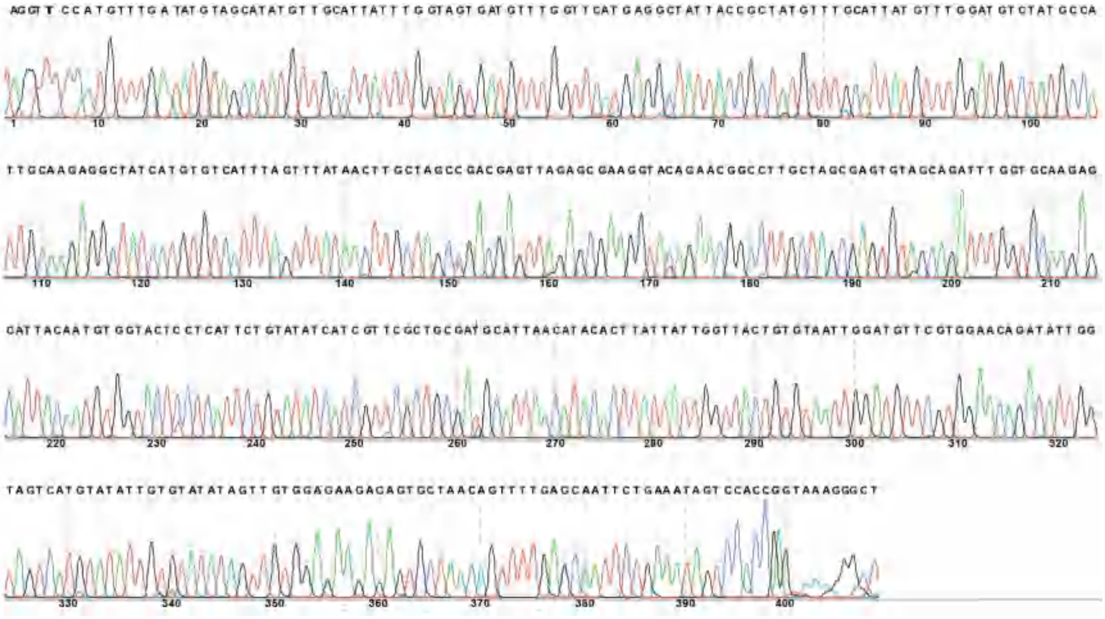

Mr-H-V114 (468 bp)

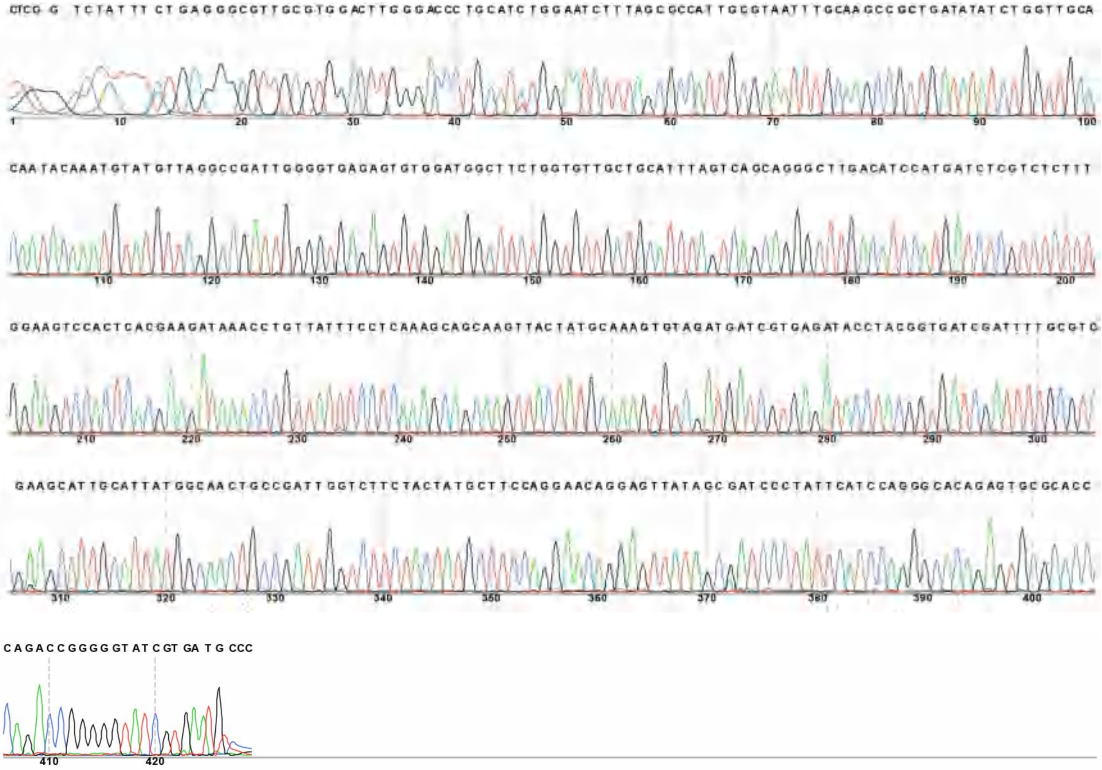

Mr-H-V116 (547 bp)

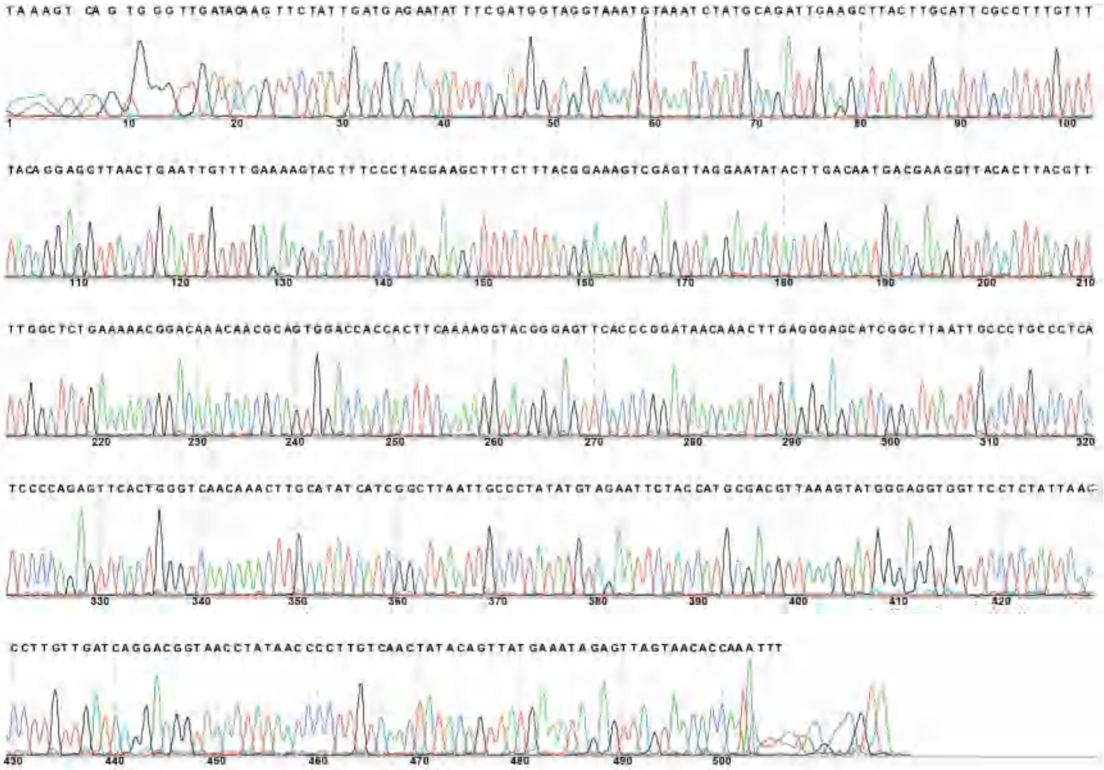

Mr-H-V119 (460 bp)

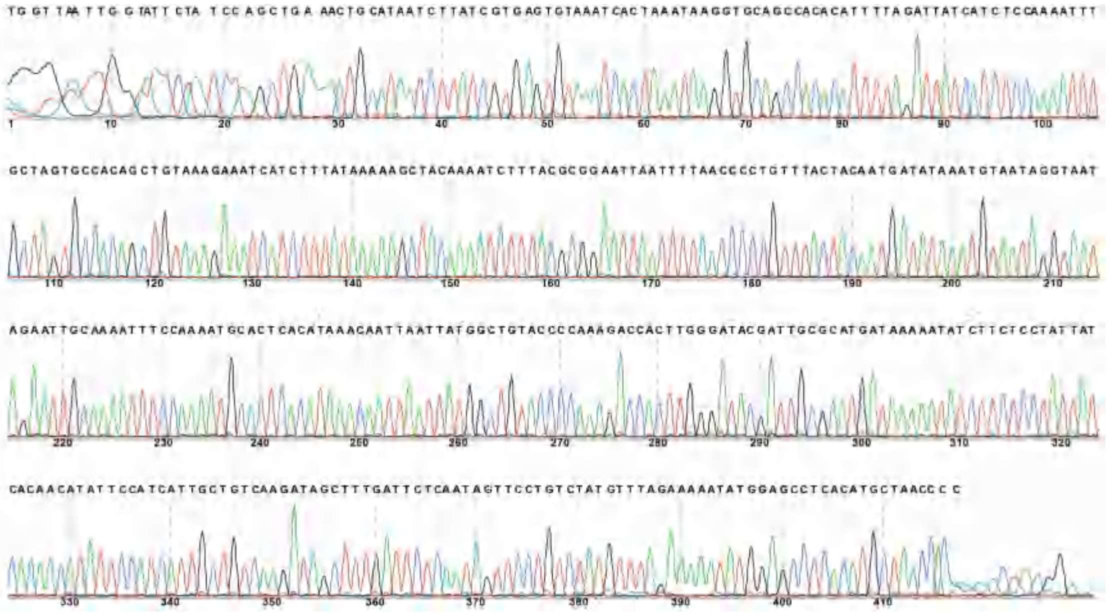

Mr-H-V120 (570 bp)

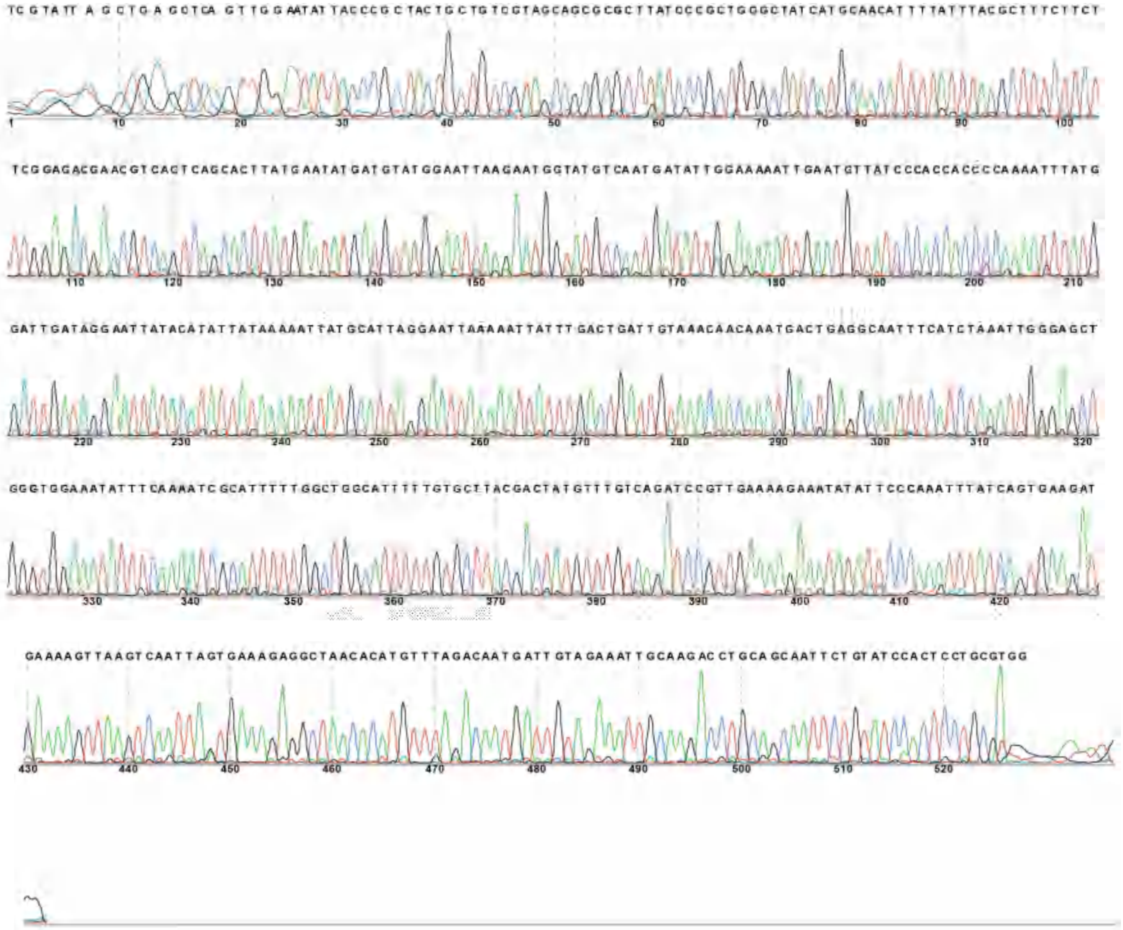

Mr-H-V122 (432 bp)

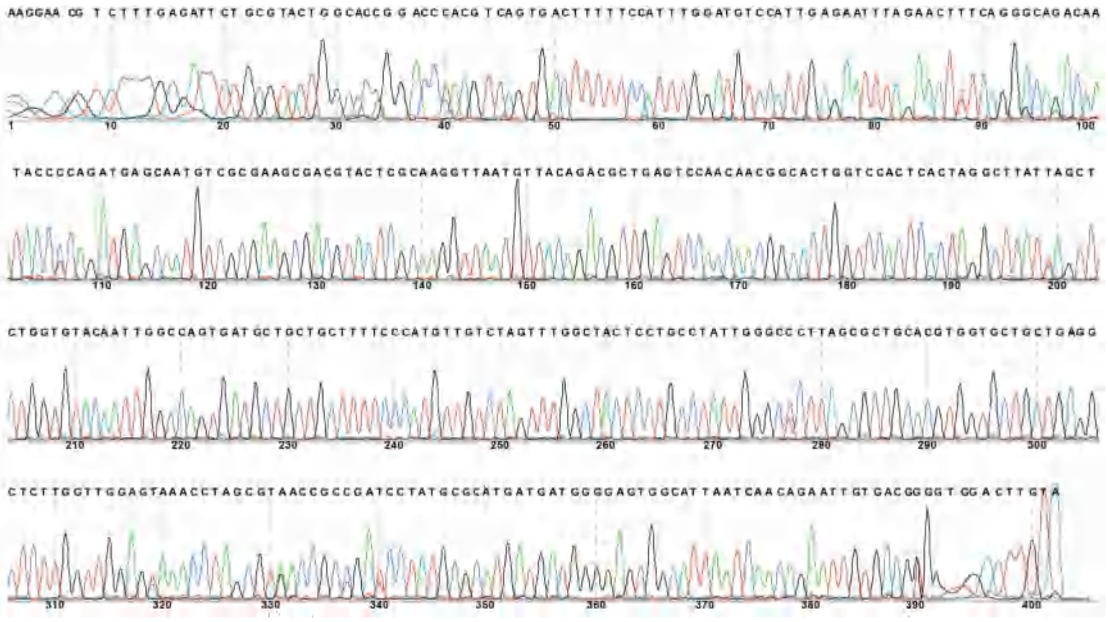

Mr-H-V123 (302 bp)

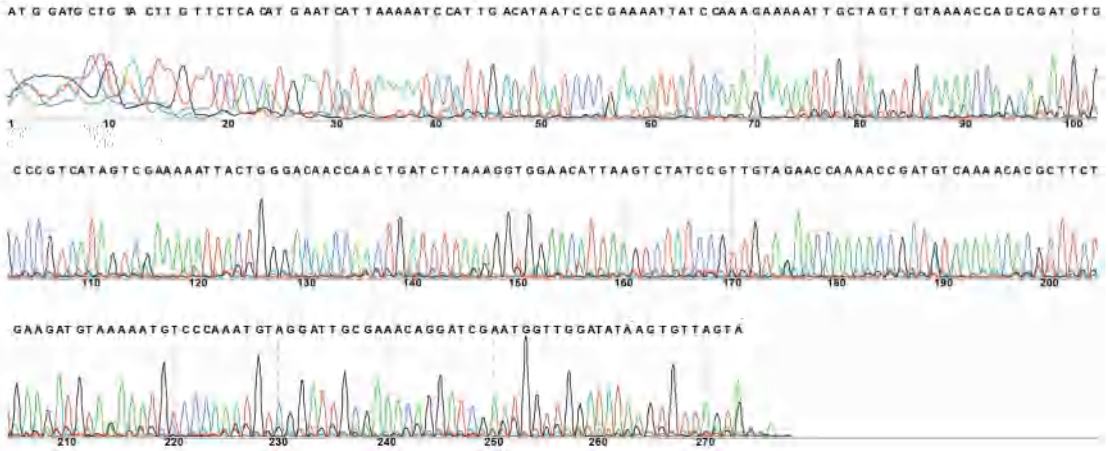

Mr-H-V130 (316 bp)

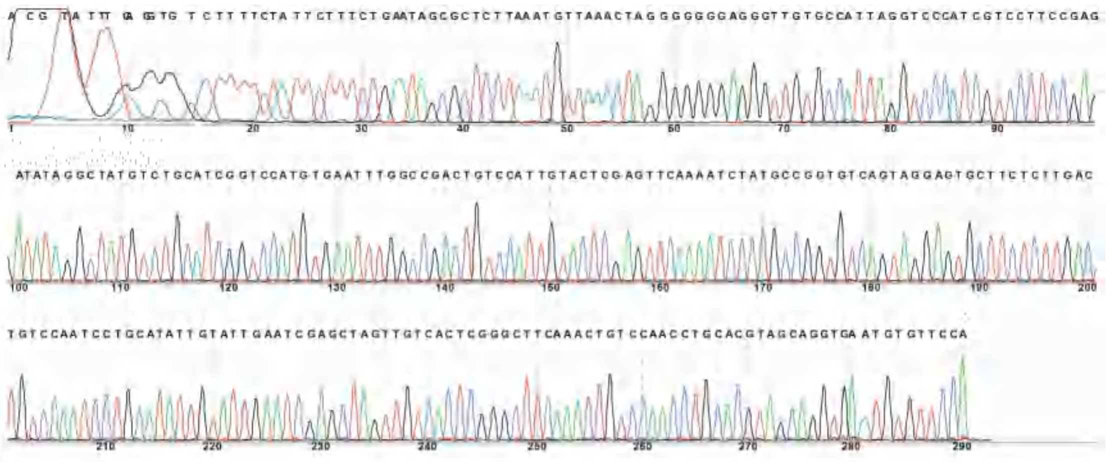

Mr-H-V131 (415 bp)

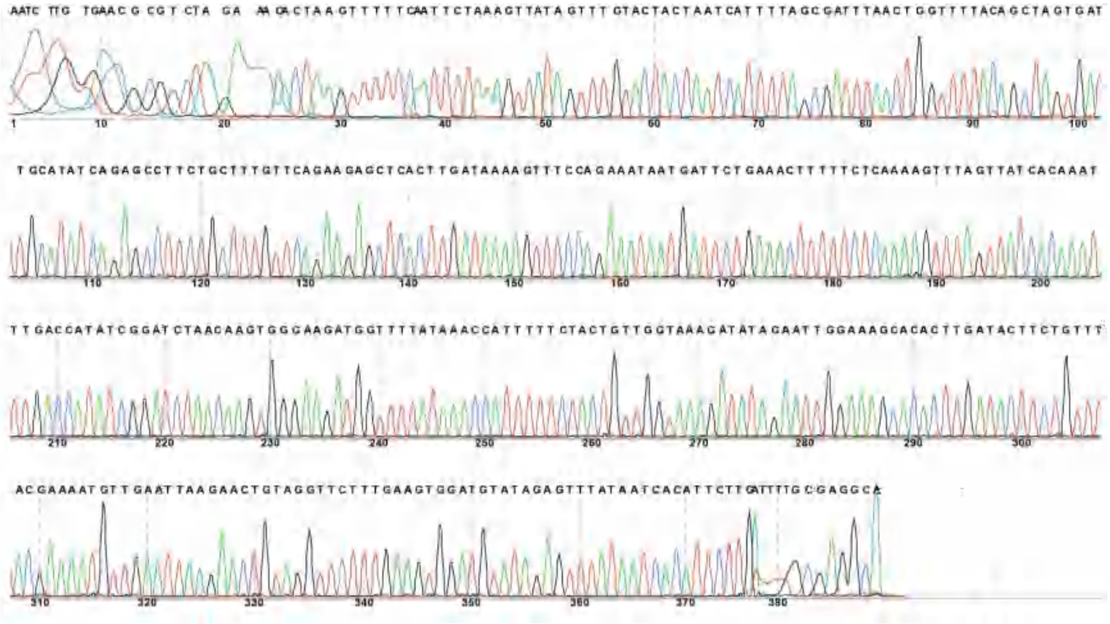

Mr-H-V132 (439 bp)

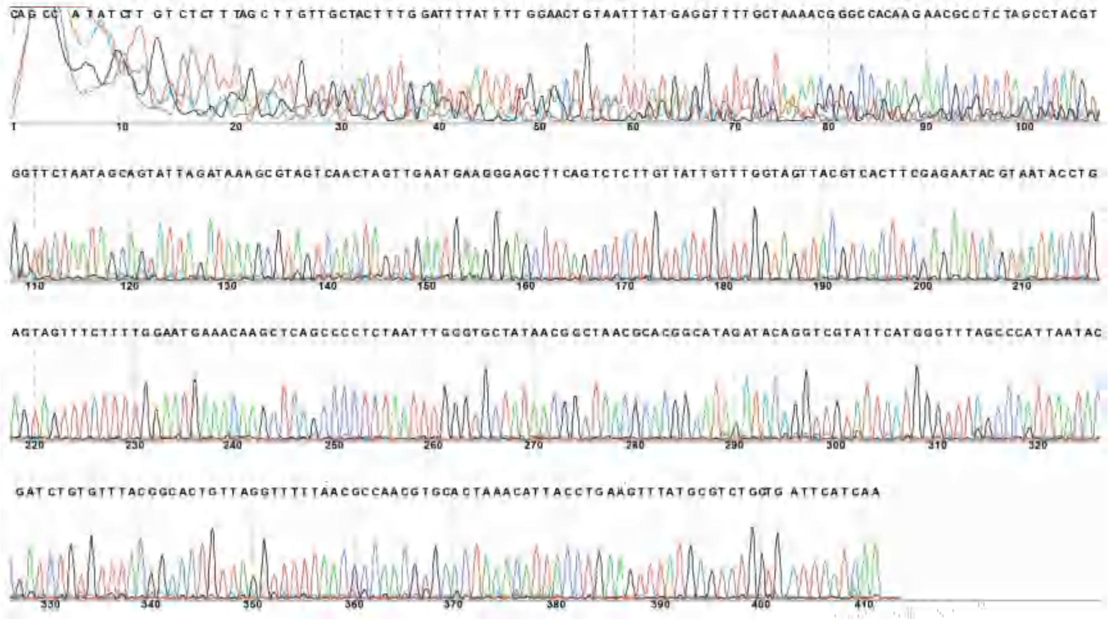

Mr-H-V136 (556 bp)

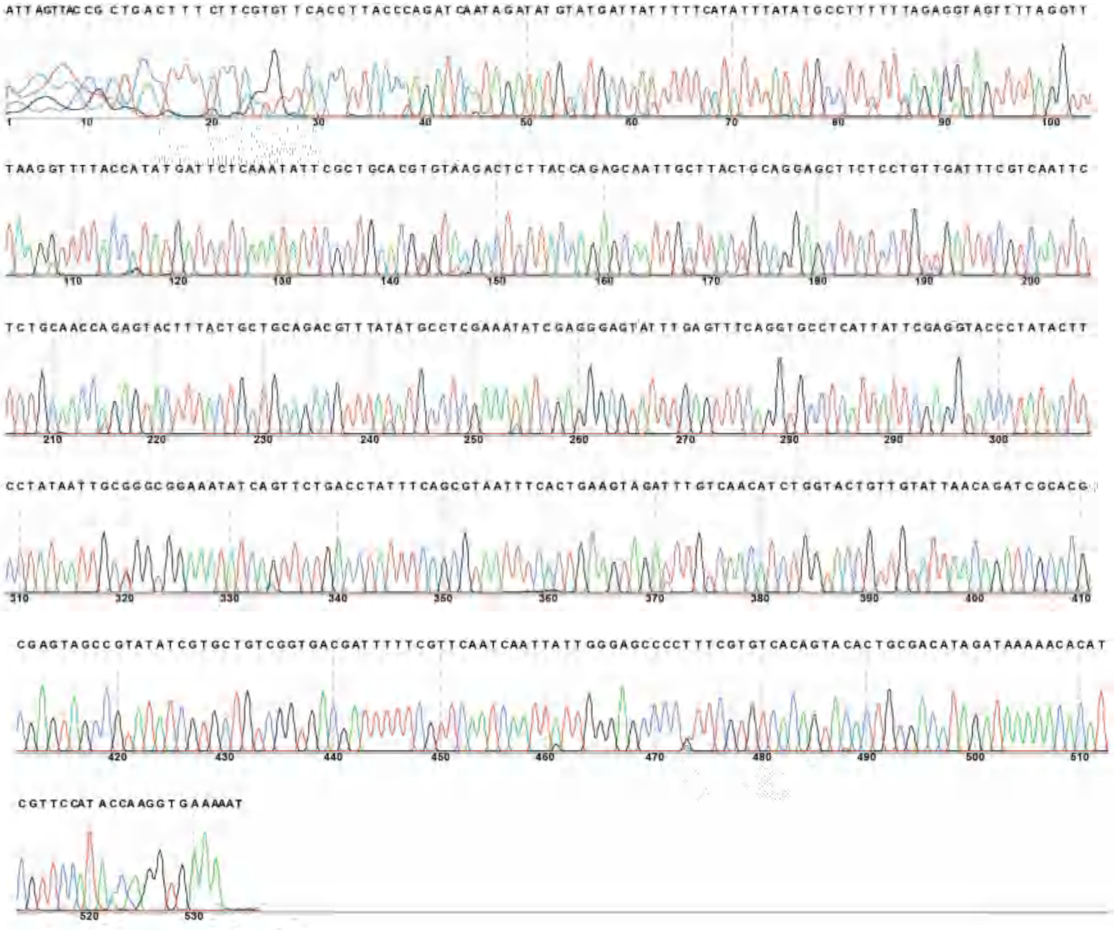

Mr-H-V139 (464 bp)

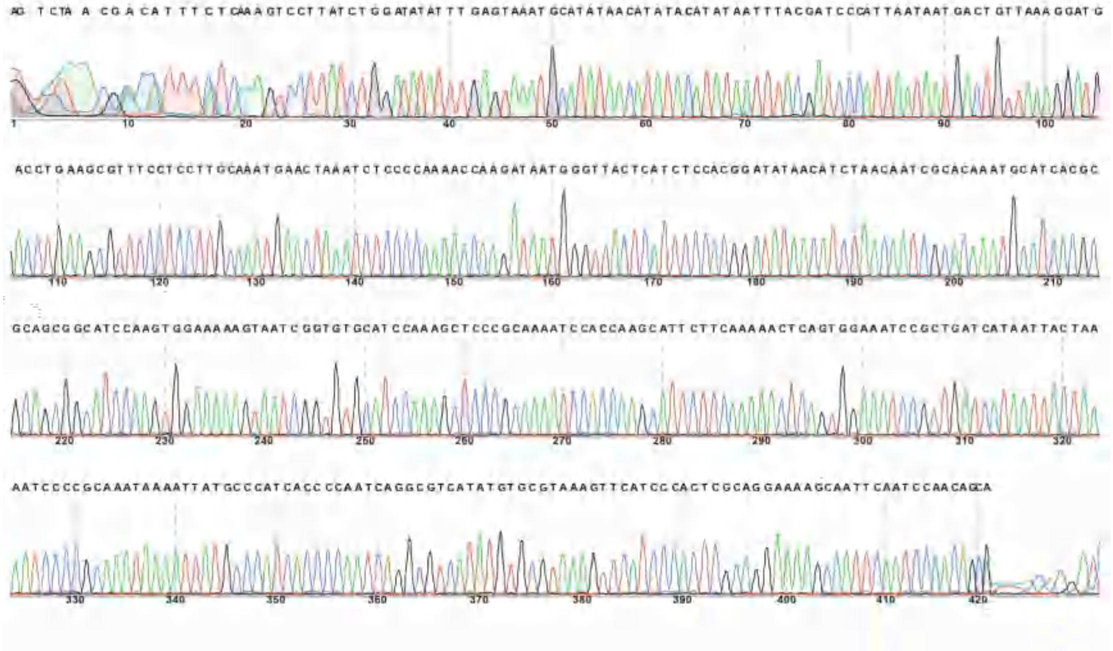

Mr-H-V140 (400 bp)

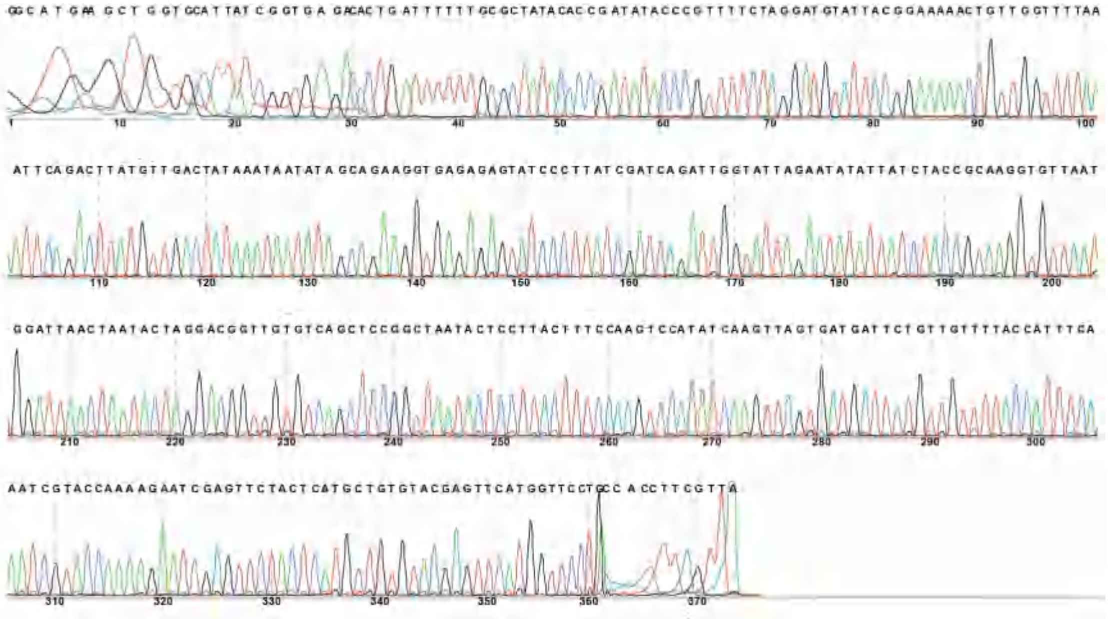

Mr-H-V143 (522 bp)

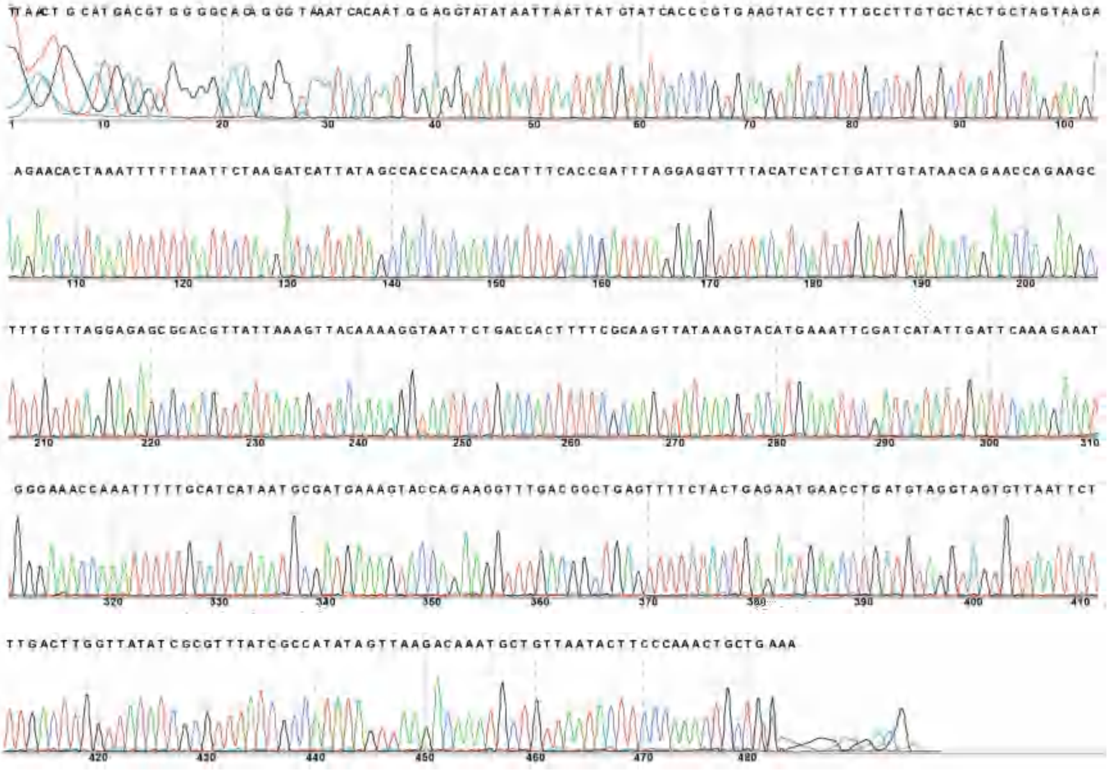

Mr-H-V147 (359 bp)

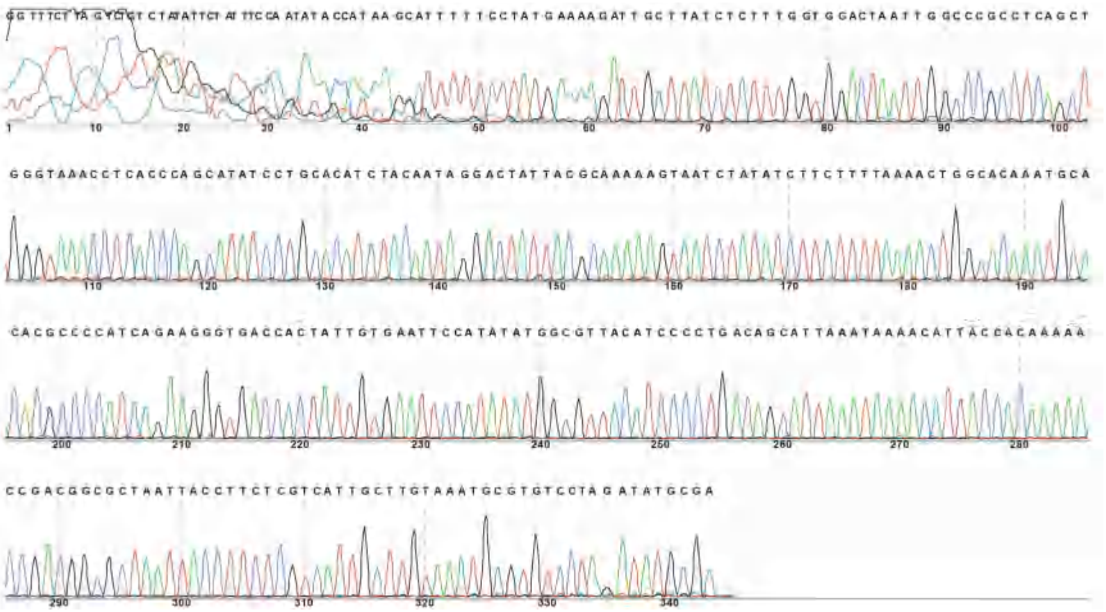

Mr-H-V150 (484 bp)

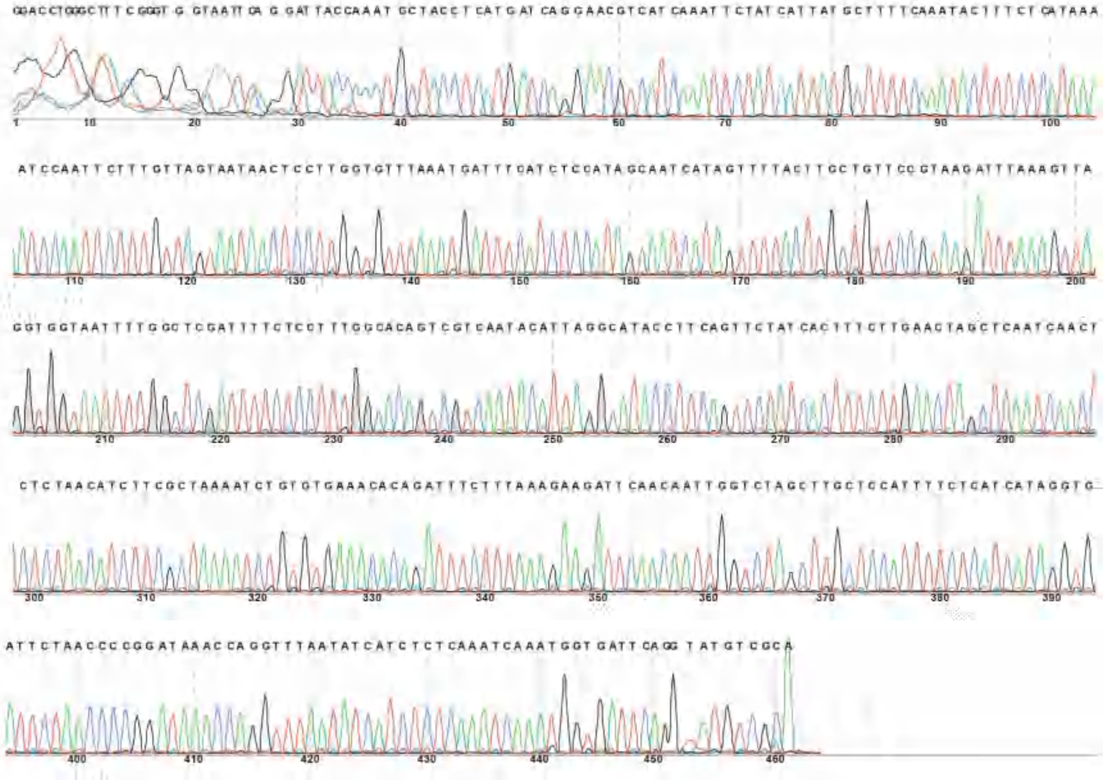

Mr-H-V151 (468 bp)

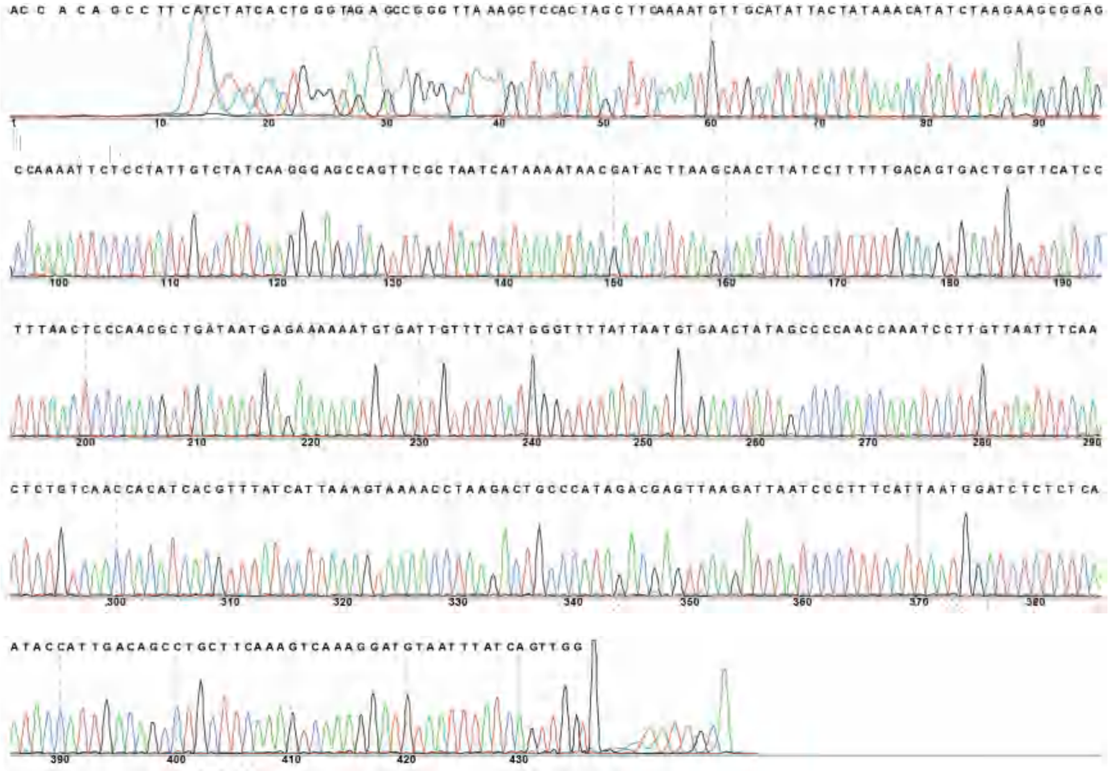

Mr-H-V152 (574 bp)

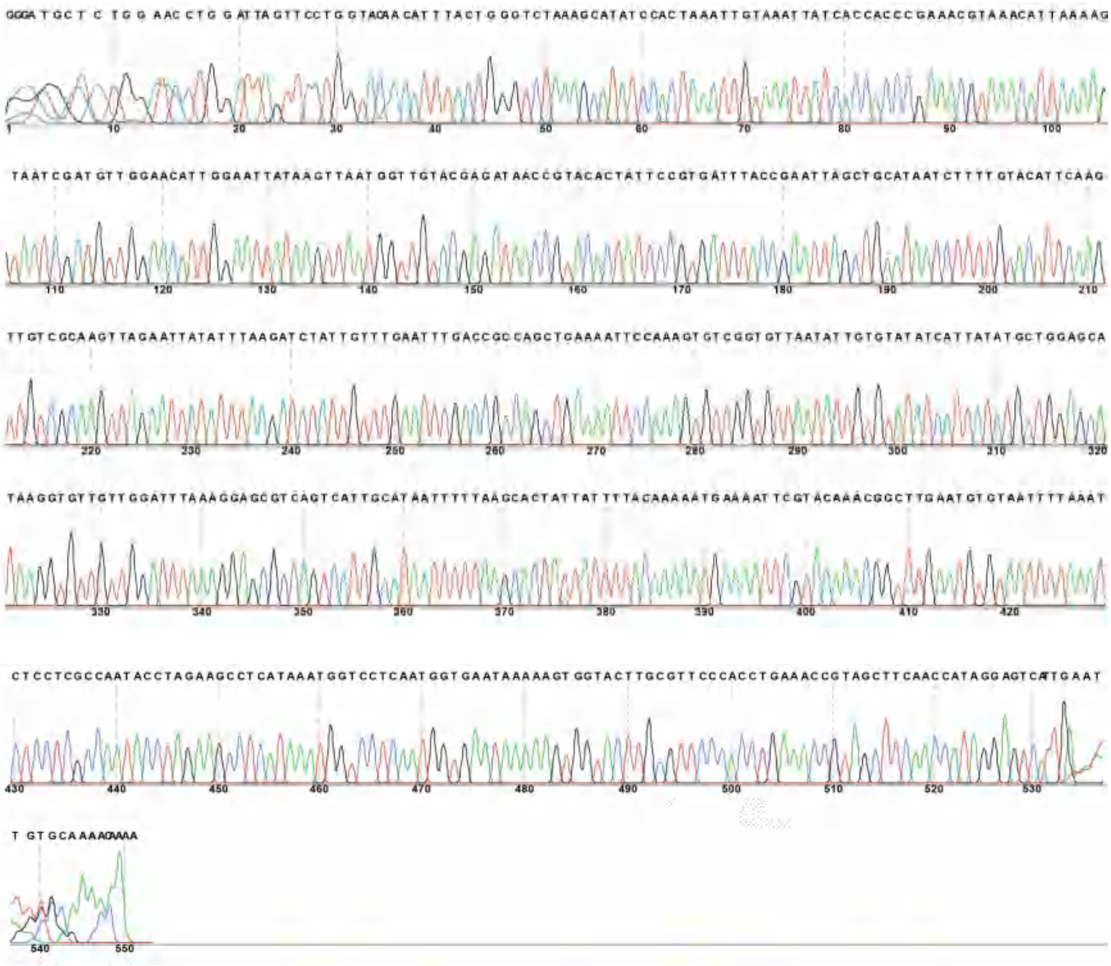

Mr-H-V157 (495 bp)

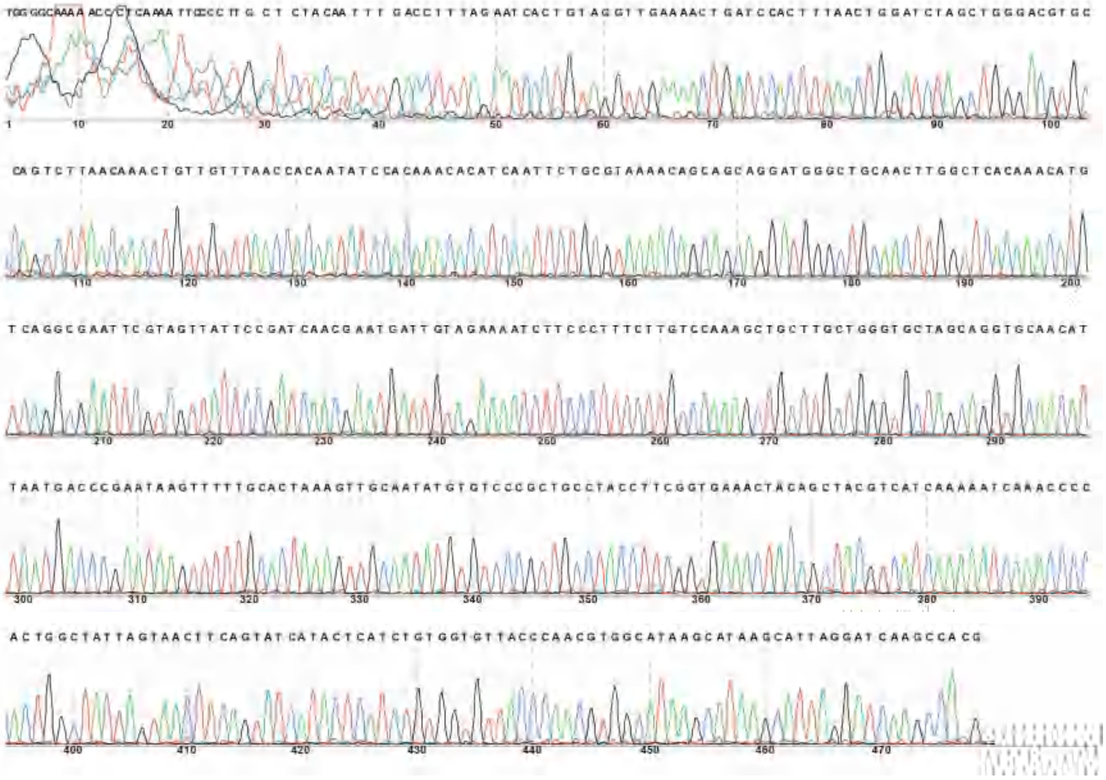

Mr-H-V159 (302 bp)

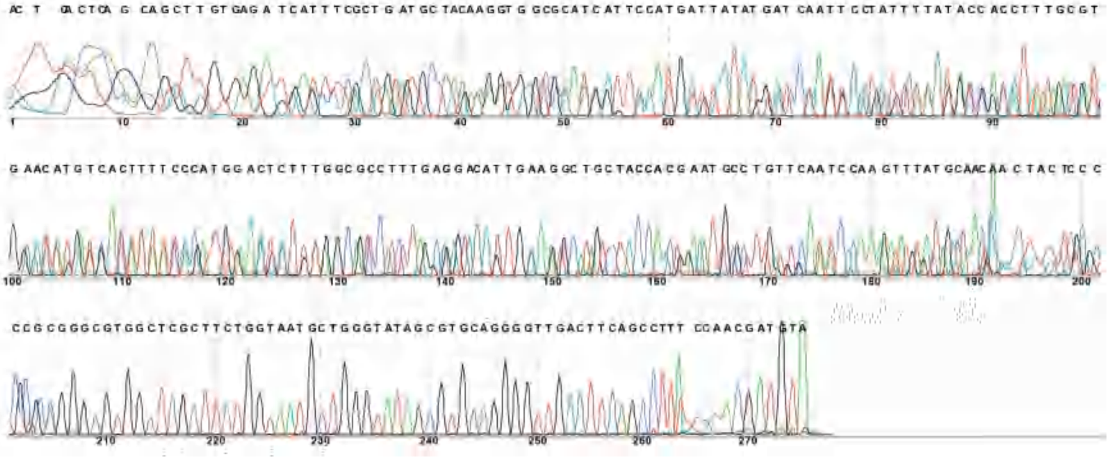

Mr-H-V164 (263 bp)

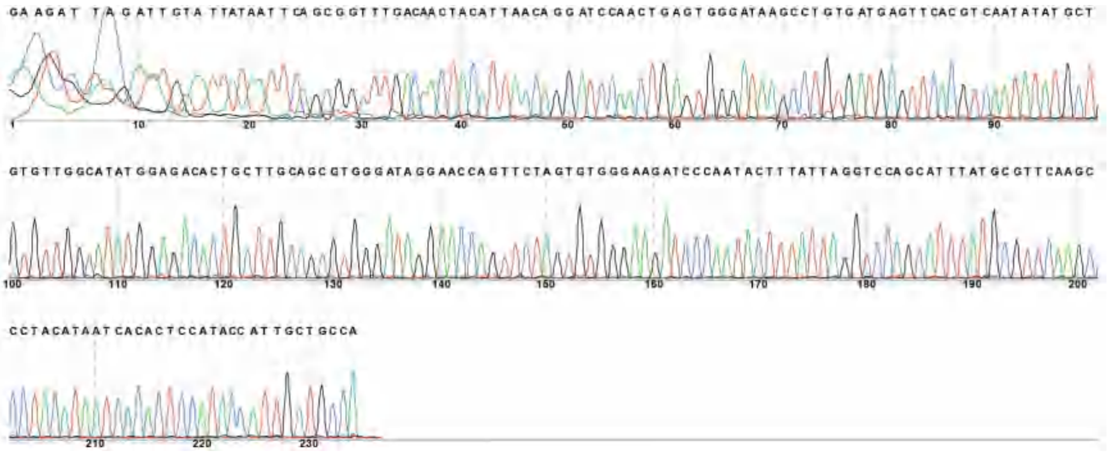

Mr-H-V165 (558 bp)

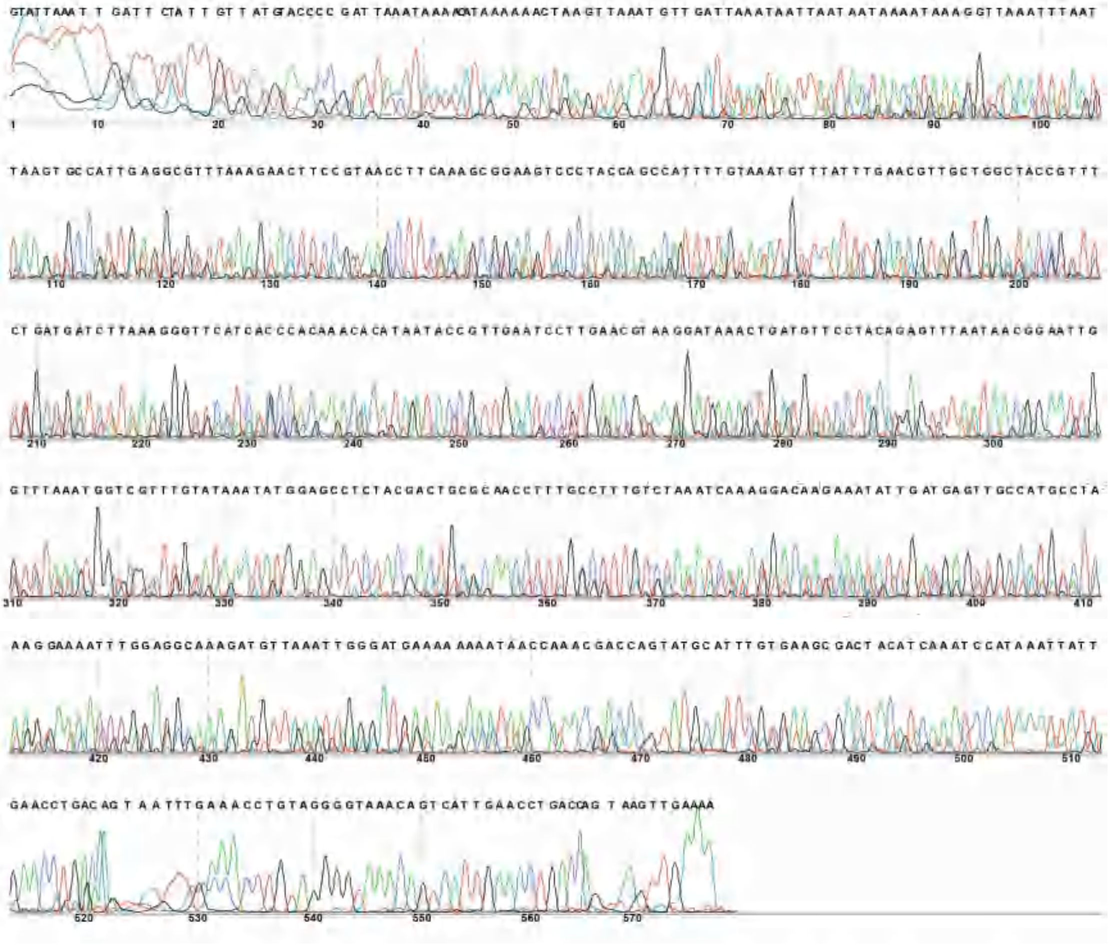

Mr-H-V166 (374 bp)

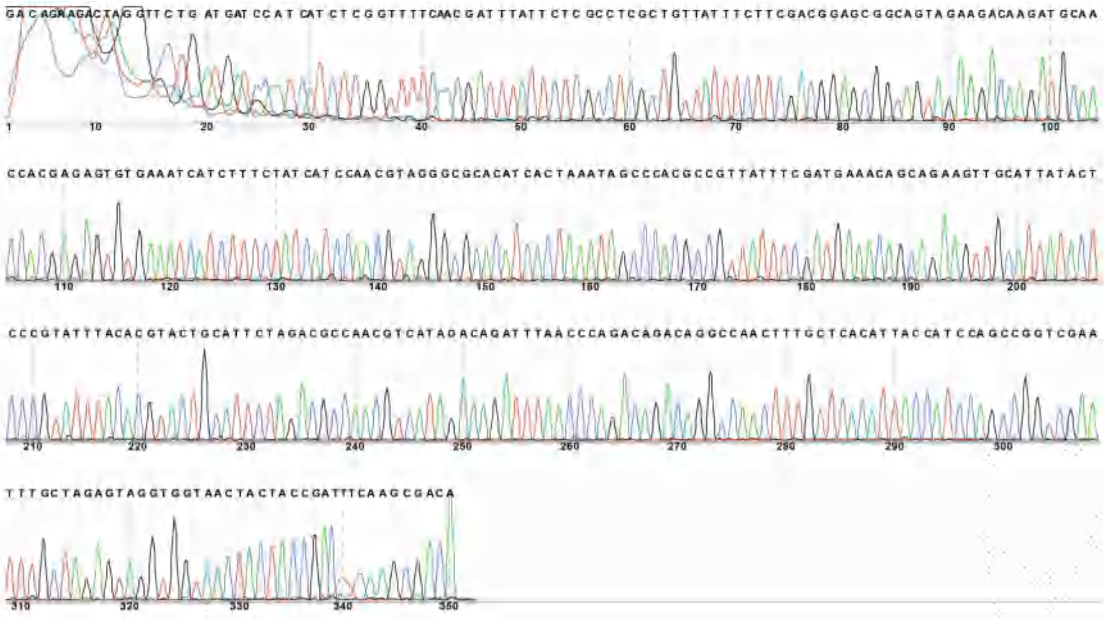

Mr-H-V175 (445 bp)

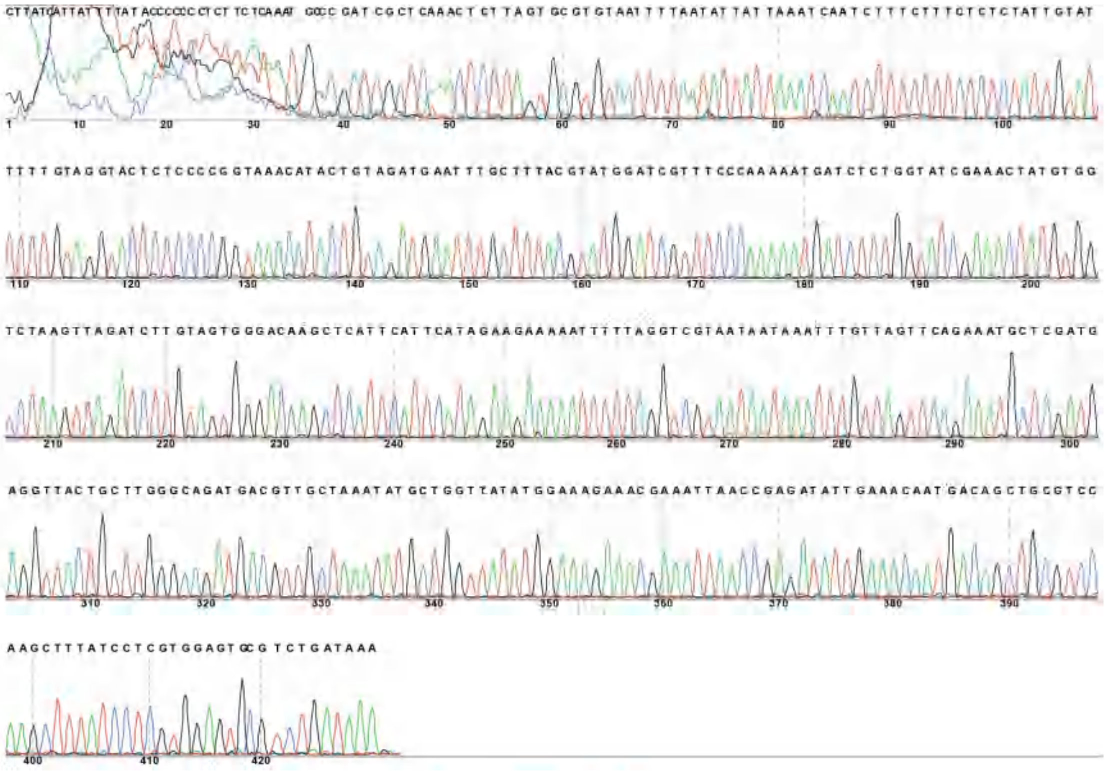

Mr-H-V176 (307 bp)

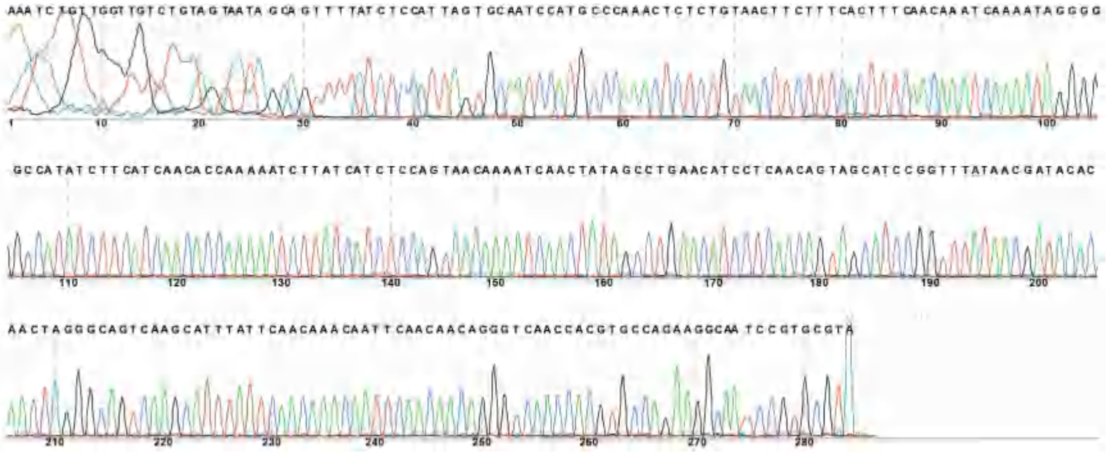

Mr-H-V178 (587 bp)

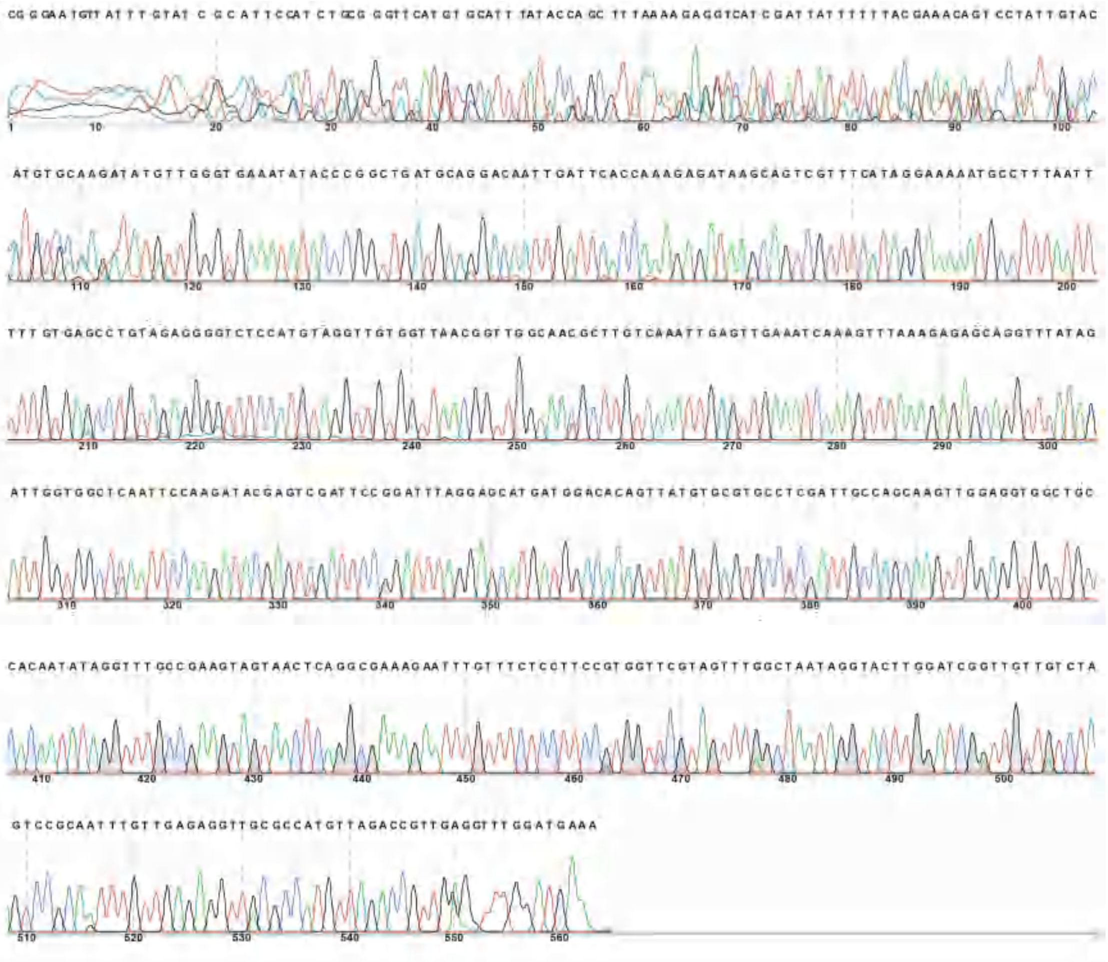

Mr-H-V183 (302 bp)

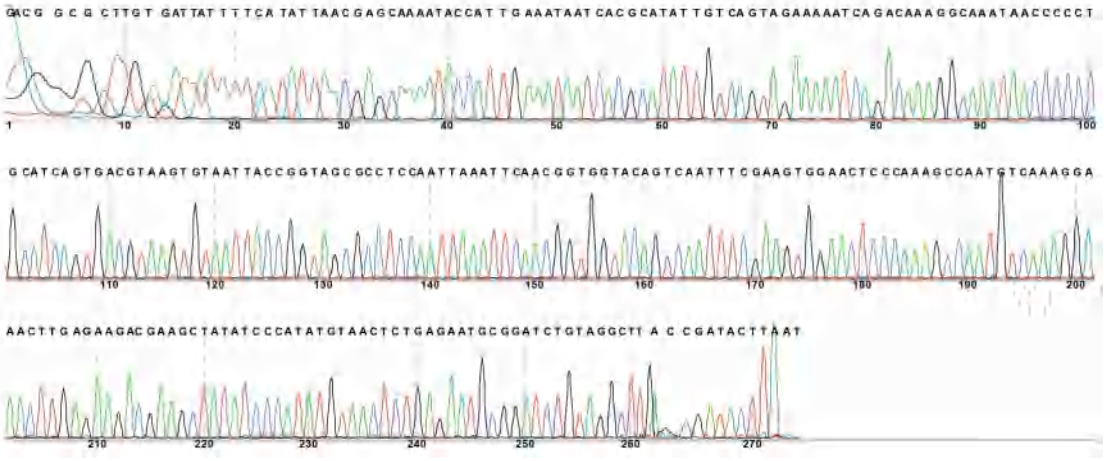

Mr-H-V185 (422 bp)

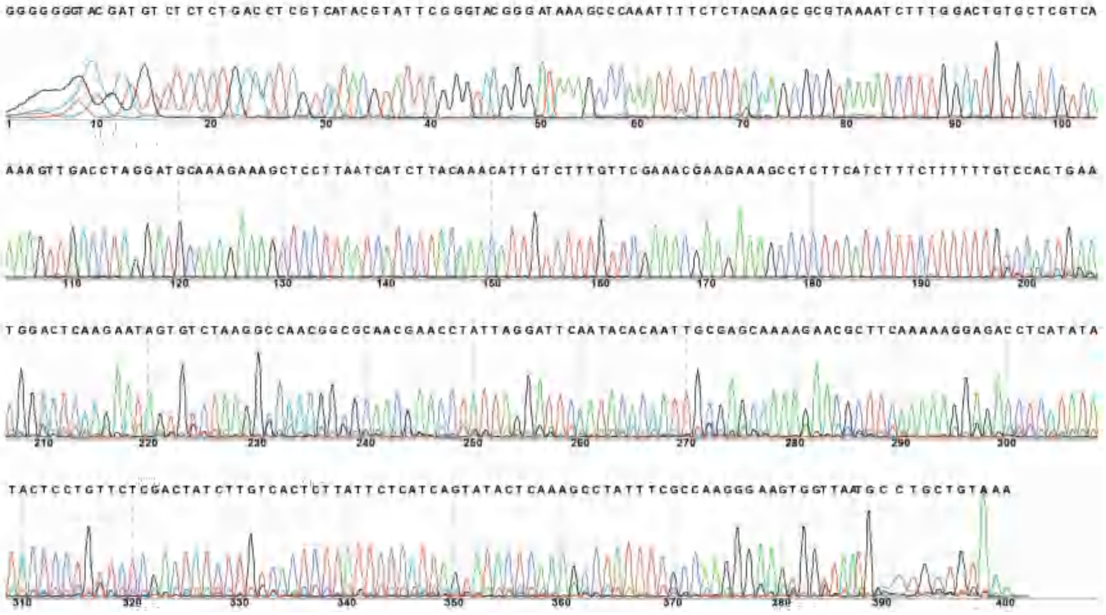

Mr-H-V187 (371 bp)

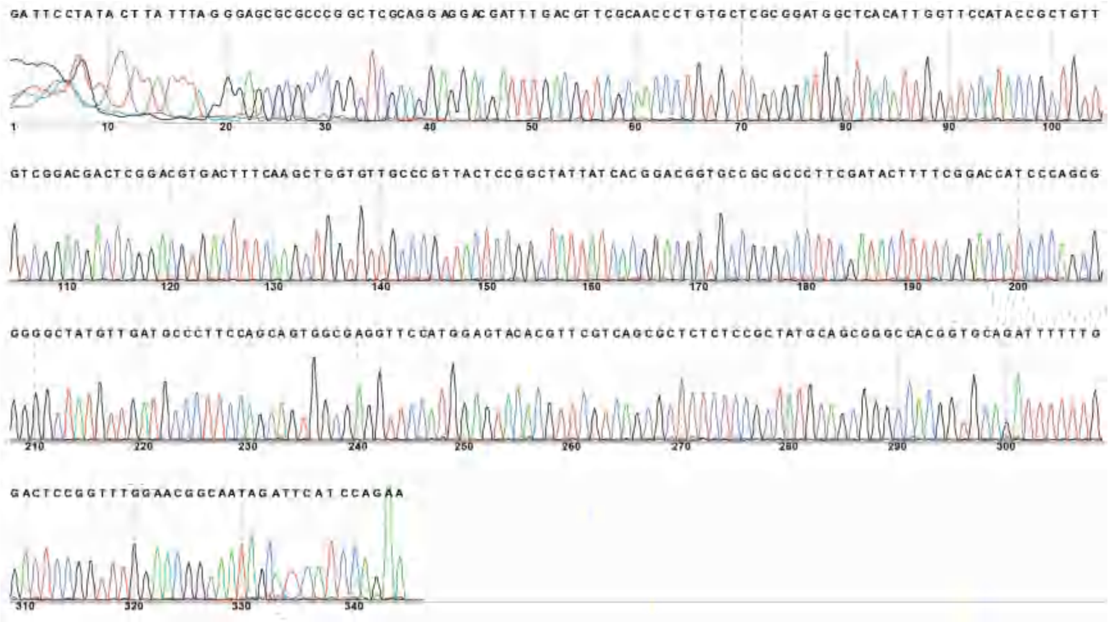

Mr-H-V190 (316 bp)

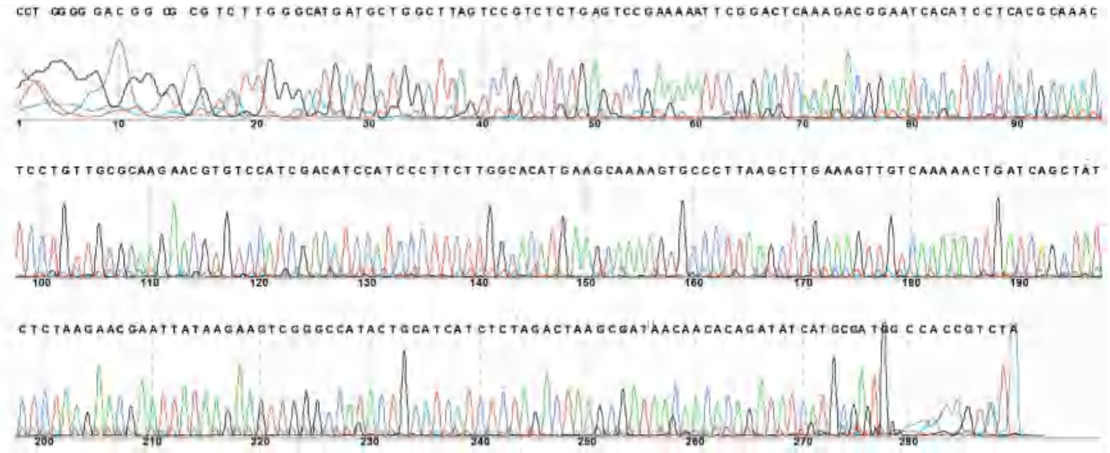

Mr-H-V192 (261 bp)

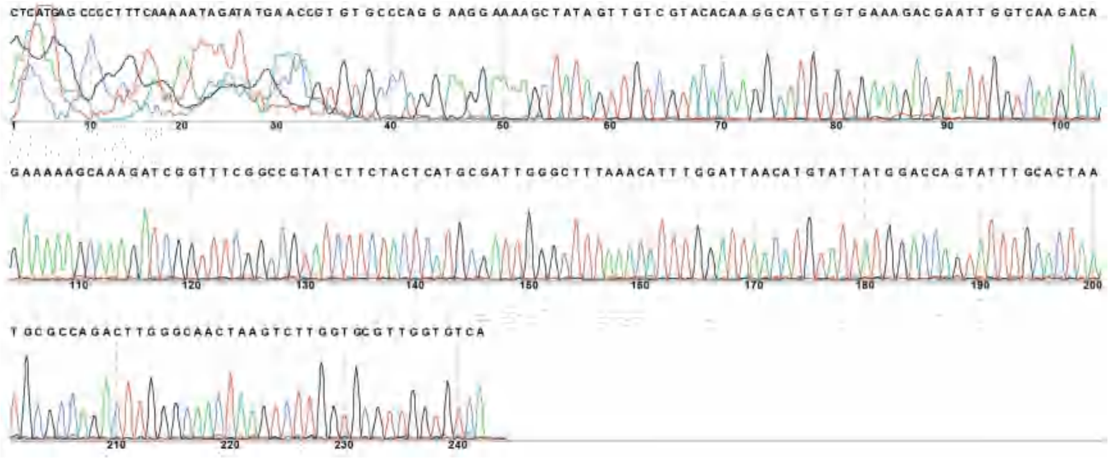

Mr-H-V193 (313 bp)

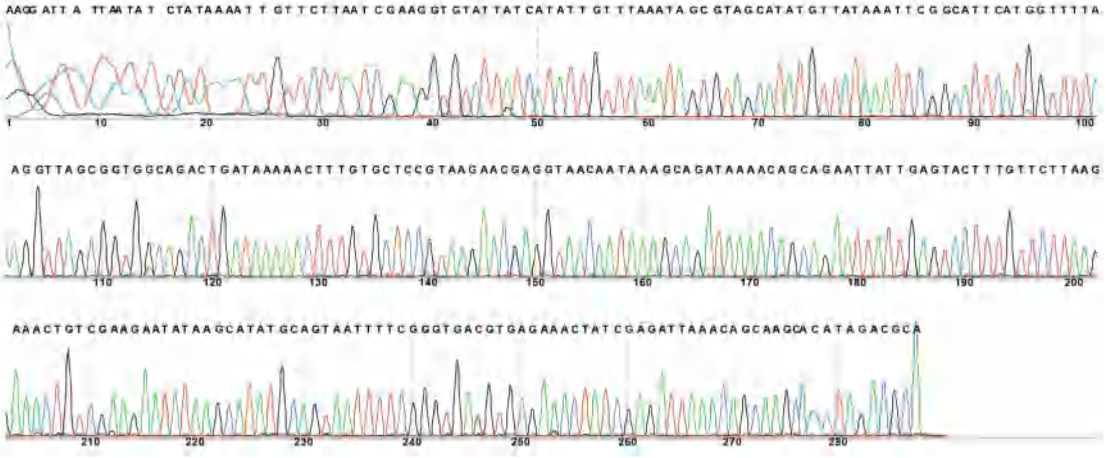

Mr-H-V196 (247 bp)

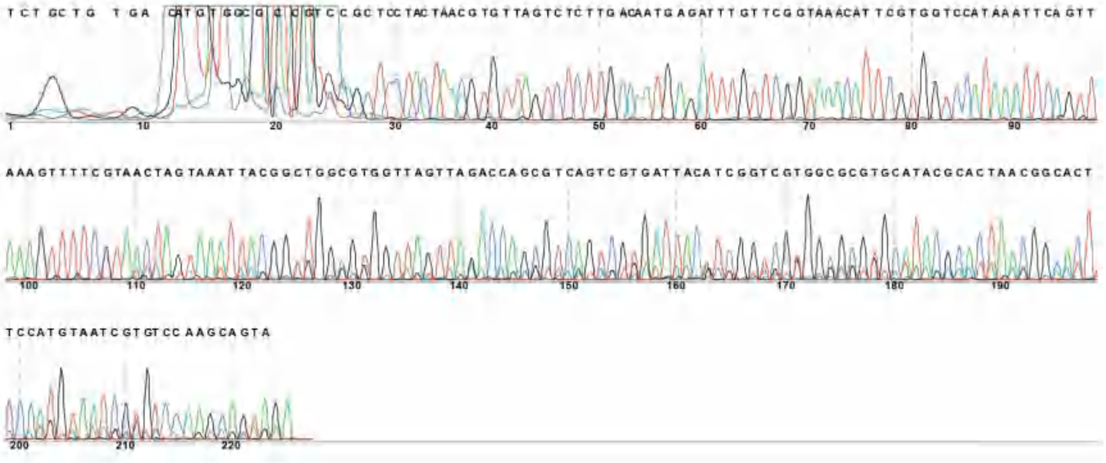

Mr-H-V209 (369 bp)

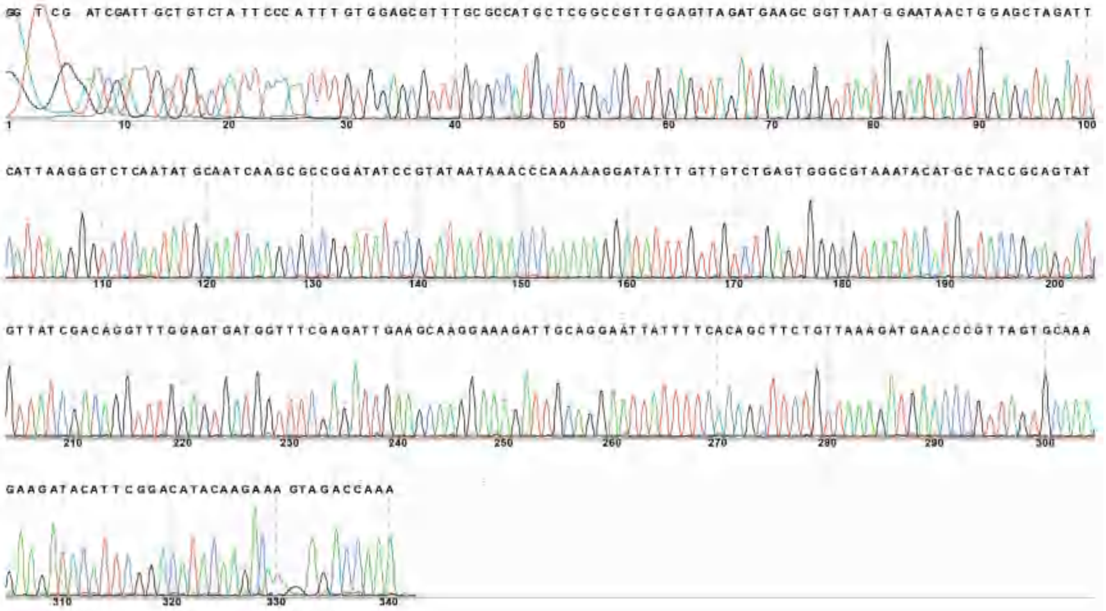

Mr-H-V210 (272 bp)

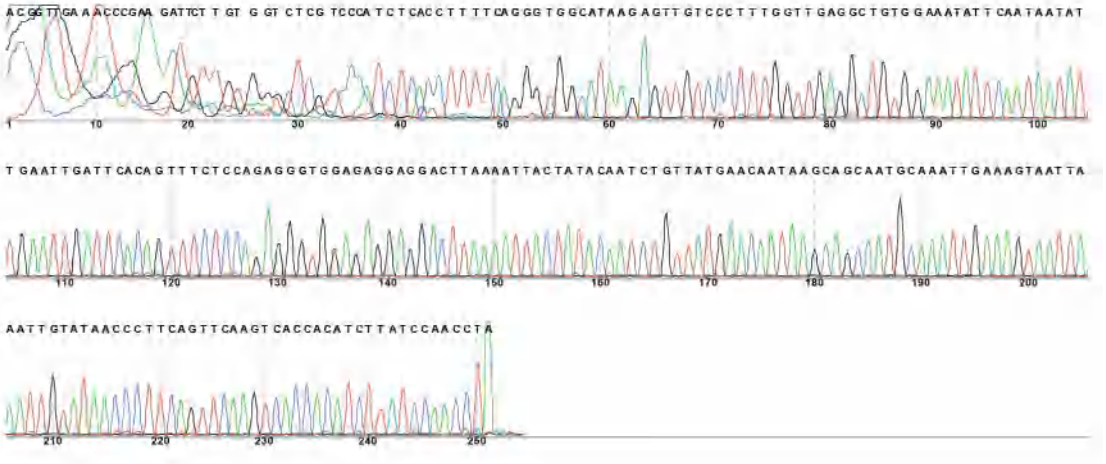

Mr-H-V211 (382 bp)

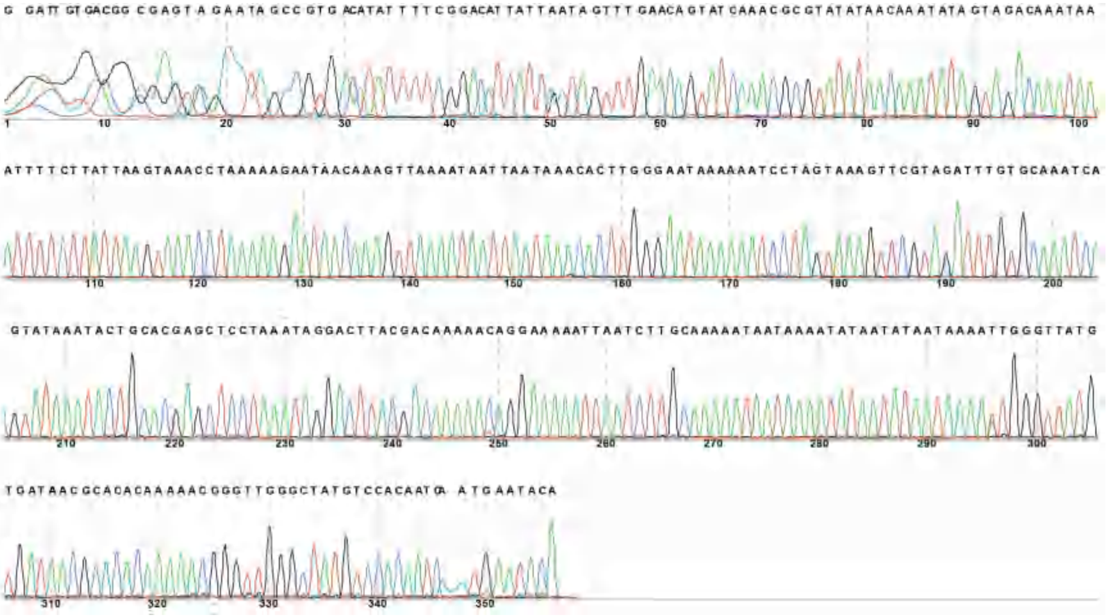

Mr-H-V214 (330 bp)

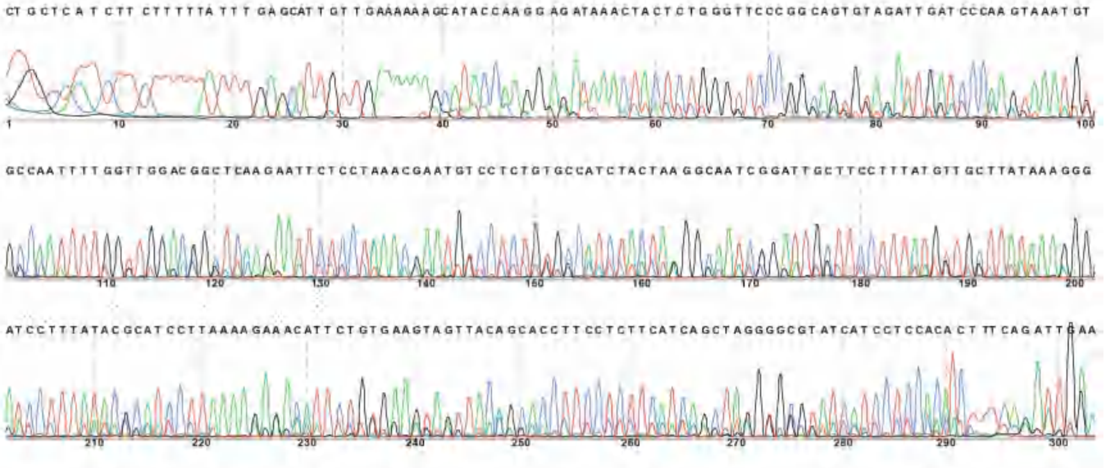

Mr-H-V218 (433 bp)

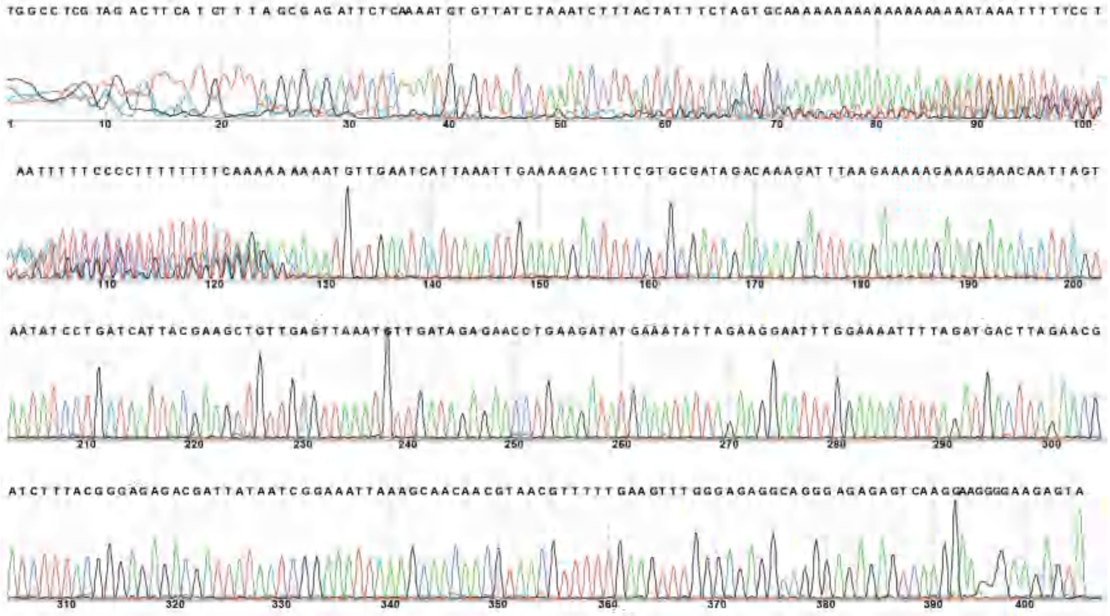

FIG S4 The diagrams of RT-PCR products sequenced by the Sanger sequencing.

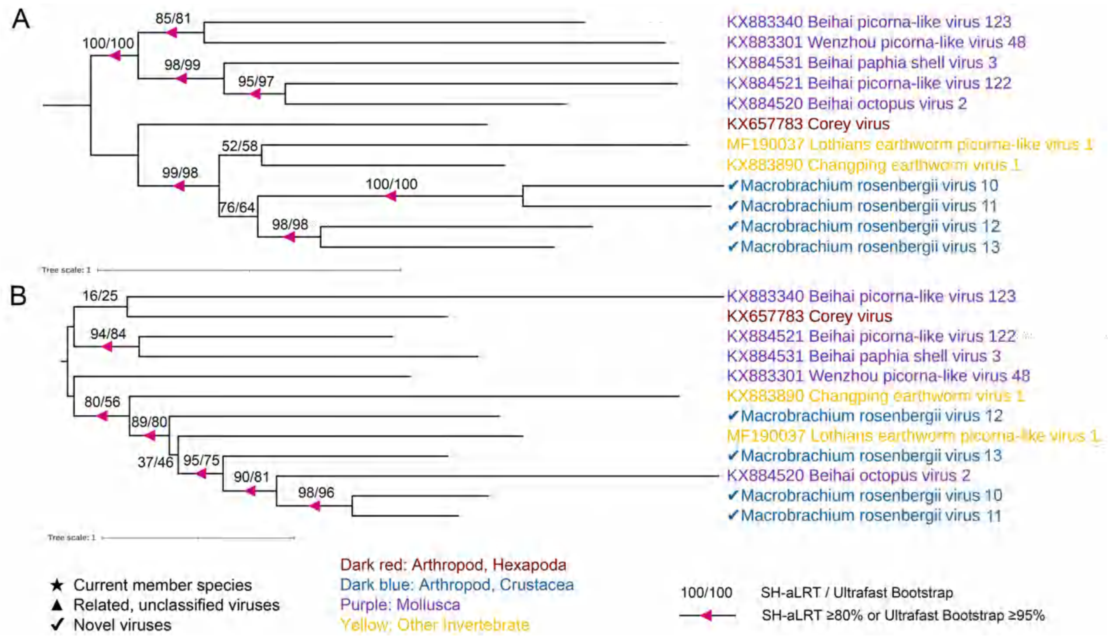

FIG S5 Mid-point rooted phylogenetic tree of members of new clade 1 in *Picornavirales*. The tree was based on (a) the amino acid sequence of the Hel-Pro-Pol replication module and neighboring conserved domains, and (b) the amino acid sequence of capsid proteins and neighboring conserved domains. The trees were inferred using the maximum likelihood approach implemented in IQ-TREE version 1.6.12. Branch support was evaluated with the SH-aLRT and UFBoot. The values are indicated at the nodes when SH-aLRT and UFBoot, and values are shown at the nodes when SH-aLRT ≥ 80% or UFBoot ≥ 95%. The names of the viruses are marked with different colors based on their host taxonomy. The star symbol signifies the current member species. The triangle symbol represents related unclassified viruses of families. The tick symbol indicates novel picorna-like viruses.
